# Characterization and therapeutic potential of phage vB_Eco_ZCEC08 against multidrug-resistant uropathogenic *Escherichia coli*

**DOI:** 10.1186/s12866-025-03903-x

**Published:** 2025-04-16

**Authors:** Assmaa H. Hussein, Salsabil Makky, Raghda Hager, Ian F. Connerton, Ayman El-Shibiny

**Affiliations:** 1 Center for Microbiology and Phage Therapy, Zewail City of Science and Technology, Giza, 12578 Egypt; 2 Basic Medical Science Department, Faculty of Medicine, King Salman International University, Eltor, South Sinai Egypt; 3 School of Biosciences, University of Nottingham, Loughborough, UK; 4 Faculty of Environmental Agricultural Sciences, Arish University, Arish, 45511 Egypt

**Keywords:** Urinary tract infections (UTIs), Uropathogenic *E. coli* (UPEC), Antibiotic resistance, Phage therapy, Molecular characterization, Artificial human urine

## Abstract

**Background:**

Urinary tract infections (UTIs) caused by antibiotic-resistant bacteria have become a significant public health concern. The increasing ineffectiveness of antibiotics has led to a renewed focus on investigating other strategies, such as bacteriophages, to target specific pathogenic bacteria and prevent future resistance.

**Results:**

This study reports the isolation and characterization of bacteriophage vB_Eco_ZCEC08 targeting uropathogenic *Escherichia coli* (UPEC). Phage vB_Eco_ZCEC08 is morphologically a non-contractile tailed phage that exhibits strong lytic activity against UPEC with a short latent period of less than 15 min and a lysis time of 20 min to produce a high burst of around 900 phage particles per host cell. vB_Eco_ZCEC08 phage activity demonstrated exceptional stability against temperature [-80–60 °C], pH [2–11], UV exposure and incubation in artificial human urine. The phage effectively reduced UPEC counts over a range of infection rates, with MOI 1 the most effective, and which resulted in the limited emergence of phage-insensitive bacteria. A whole-genome study of the 47.926 bp vB_Eco_ZCEC08 phage identified one tRNA gene and 84 predicted genes. Comparative genomics and phylogenetic analysis suggest that the vB_Eco_ZCEC08 phage belongs to the same genus as the *Salmonella* phage vB_SenS_ST1 but represents a new species. Phage vB_Eco_ZCEC08 showed minimal cytotoxicity against human urinary bladder cancer and skin fibroblast cell lines.

**Conclusion:**

vB_Eco_ZCEC08 phage demonstrates strong selective lytic activity against UPEC in the absence of any lysogenic behavior. These properties coupled with inherent physiochemical stability and low cytotoxicity support the development of vB_Eco_ZCEC08 as an alternative treatment for multidrug-resistant UPEC.

**Supplementary Information:**

The online version contains supplementary material available at 10.1186/s12866-025-03903-x.

## Background

Urinary Tract Infections (UTIs) are among the most prevalent bacterial diseases, affecting around 150 million individuals globally each year [1]. The classification of UTIs includes both uncomplicated and complicated cases. Uncomplicated UTI frequently affects healthy individuals, causing conditions like cystitis (lower UTI) and pyelonephritis (upper UTI). On the other hand, complicated UTIs are associated with additional health issues such as immunodeficiency, pregnancy, renal failure or transplantation, neurological diseases, and the existence of medical drains or catheters [2, 3]. UTIs are caused by various uropathogenic bacteria, including *E. coli*,* Klebsiella pneumoniae*,* Proteus mirabilis*,* Staphylococcus aureus*, and *Pseudomonas aeruginosa* in addition to other bacteria and fungi [4–6]. Uropathogenic *E. coli* (UPEC) is considered the most dominant causative agent, associated with an average of 85% of uncomplicated and complicated UTIs [7]. A meta-analysis study reported UPEC to display predominant virulence factors including immune suppressors, adhesins, toxins, and siderophore systems that boost urinary tract infectivity [8]. Antibiotics are routinely prescribed for UTI treatment, which results in the recovery of many patients suffering from these infections [9, 10]. Nevertheless, the antibiotic resistance crisis has evolved to become one of the most serious human health threats of the twenty-first century [11]. A study estimated that by 2050, the number of deaths from antibiotic-resistant bacterial infections will reach 10 million, surpassing the global death toll from cancer without the discovery of alternative treatments [12]. Several factors are contributing to the acceleration of antibiotic resistance rates, including misuse, inappropriate prescription, and the excessive consumption of antibiotics. *E. coli* is among the ESKAPEE pathogens recognized as the most critical multidrug-resistant (MDR) pathogens implicated in nosocomial infections, and which include *Enterococcus faecium*,* S. aureus*,* K. pneumoniae*,* Acinetobacter baumannii*,* P. aeruginosa*,* Enterobacter spp.* and *E. coli* [13–15]. According to the World Health Organization (WHO) Bacterial Priority Pathogens List for 2024, the third-generation cephalosporin-resistant Enterobacterales, including cephalosporin-resistant *E. coli* (3GCRE), are designated as critical pathogens (Priority 1 group) with a global disease burden and economic impact. This situation underscores the urgent need for the discovery of alternative, effective antibacterial agents [16]. However, the innovation of novel antibiotics is currently constrained by financial limitations and technological development [17]. A potential alternative is bacteriophages (phages), since phages are prokaryotic viruses and natural predators of bacteria [18]. Phages can exhibit high specificity to their bacterial hosts but require careful evaluation to minimize their effects on off-target bacteria, such as those present in the human microbiota [19]. Moreover, phages act as a self-dosing treatment since they replicate inside the host bacteria, generating new phages as long as the susceptible bacteria are present [20]. Many studies have suggested that phage therapy holds promise as an alternative treatment, whether used as mono or combination therapy, particularly for UTIs caused by antibiotic-resistant bacteria, which often lead to chronic infections [21, 22].

In the current study, we aimed to isolate and characterize a potent virulent phage against MDR uropathogenic *E. coli*. Additionally, we sought to assess the fundamental characteristics of the candidate phage isolate, including the physiochemical stability, molecular characterization, phylogenetic analysis, phage-killing efficiency, phage safety with human cell lines and the dynamics of phage-bacteria replication in artificial human urine.

## Methods

### Bacterial isolation, purification, and stocking

Twenty-nine *E. coli* of UTI sources were gifted from the microbiology unit of Acculab at Rofayda Hospital, Giza, Egypt, in 2022. The bacterial isolates were cultured on Eosin Methylene Blue EMB agar (Oxoid, UK) and were incubated overnight at 37 °C to allow morphological examination. This step was repeated 3 times to ensure the presence of pure isolates. Selected colonies were subsequently sub-cultured overnight into tryptone soy broth (TSB) (Oxoid, UK) at 37 °C with shaking at 70 rpm. All isolates were then preserved in 20% (w/v) glycerol TSB at -80 °C.

### Identification and pathogenicity detection via polymerase chain reaction (PCR)

Bacterial DNA was isolated from fresh culture on tryptone soy agar (TSA) (Oxoid, UK), following the Gussow & Clackson, (1989) protocol [23]. Briefly, a single colony from each strain was dissolved in 0.5 mL of nuclease-free water and boiled at 100°C for 5 min. The mixture was then centrifuged at 1000 rpm for 5 min, and 5 µL of the supernatant was used for the PCR reactions. The bacterial strains were identified using *E. coli* specific primers (FP: 5’-AAGAAGCACCGGCTAACTCC-3’ and RP: 5’-CGCTTCTCTTTGTATGCGCC-3’) with an annealing temperature of 60 °C. For EC-08 (phage-host bacteria), a single colony was identified based on the proteome content using VITEK MS (bioMérieux, France) for precise microbial identification. Pathogenicity factors associated with the isolated bacteria were detected by PCR. Supplementary Table S1 details of the pathogenicity genes, including virulence and antibiotic resistance genes, along with PCR conditions for each gene. The PCR amplicons were separated using the electrophoresis system (Bio-Rad, USA) and a 1% (w/v) agarose gel (Oxoid, UK). All bands were examined in comparison to the 100 bp DNA ladder (Transgene, France).

### Antimicrobial susceptibility testing (AST)

Antimicrobial susceptibility testing was conducted according to the Performance Standards for Antimicrobial Susceptibility Testing (CLSI, 2022) [24] on uropathogenic *E. coli* strains using sixteen antibiotic agents from nine different classes. The antibiotics and their respective concentrations were as follows: ampicillin (AM 10), amoxicillin-clavulanate (AMC 30), ampicillin-sulbactam (SAM 20), piperacillin-tazobactam (TPZ 110), ceftazidime (CAZ 30), cefuroxime (CXM 30), cefoperazone (CFP 75), ertapenem (ETP 10), imipenem (IPM 10), meropenem (MRP 10), gentamycin (CN 10), tobramycin (TOB 10), ciprofloxacin (CIP 5), trimethoprim-sulfamethoxazole (SXT 25), chloramphenicol (C 30), tetracycline (TE 30) (Oxoid, UK). A volume of 100 µL of each bacterial culture (OD_600_ ~ 0.3) was swabbed onto the TSA plates. Subsequently, the antibiotic discs were placed onto the bacterial lawns and incubated for 16 h at 37 °C. The inhibition zone diameters were measured in triplicate for each antimicrobial agent, and the average diameters were calculated. These values were then compared to the reference zones in CLSI 2022. Based on the calculated results, the multiple antibiotic resistance (MAR) index was assessed for each strain separately, providing insights into the antimicrobial resistance profiles of the strains under study [25].

### Phage isolation, purification, and propagation

The virulent phage was isolated following the method of Carlson (2005) [26]. Briefly, different medical sewage samples were collected from Rofayda Hospital, Giza, Egypt. These samples were centrifuged at 4000 rpm to eliminate all waste and debris and then filtered using 0.2 μm syringe filters. For phage enrichment, the filtrate was incubated with a culture of the bacterial collection (OD_600_ ~ 0.3) at 37 °C and 70 rpm for 4 h. Afterwards, the mixture was centrifuged at 7000 rpm and 4 °C for 15 mintues.

Each bacterial strain was used separately for double-layer agar preparation. Specifically, 50 µL of the culture (OD_600_ ~ 0.3) was combined with 5 mL of molten top-agar of 45 °C and poured onto sterile TSA plates. A spot assay was performed by spotting 10 µL of each filtrate onto each bacterial strain. Subsequently, all agar plates were incubated at 37 °C for 18 h.

For purification, single phage plaques were cored from the agar using a micropipette tip and placed in a sterile SM buffer before overnight elution at 4 °C. The isolated phages were centrifuged at 7000 rpm and 4 °C for 15 mintues. The supernatant was then spotted on the host bacterial strain after a 10-fold serial dilution for each phage. The purification steps were repeated six times for each phage to ensure phage stock purity.

Phage amplification was performed according to Carlson (2005) with a few modifications [26]. In brief, 30 mL of the bacterial culture (~ 10^6^ CFU/ml) was infected with the phage at a multiplicity of infection (MOI) ~ 0.1) and incubated for 3 h at 37 °C and 70 rpm. The phage stock was incubated with chloroform (2%) to allow bacterial lysis and release of phages before two rounds of centrifugation. In the first round, low-speed centrifugation at 7000 rpm for 15 mintues allowed for the precipitation of any bacterial debris, and the supernatant was transferred to a sterile falcon tube. Thereafter, the high-speed centrifugation (second round) at 12,000 rpm centrifugation for 1 h was performed. The supernatant was discarded, and the pellet was suspended in 15 mL of SM buffer and stored at 4 °C.

To confirm the purity of the vB_Eco_ZCEC08 phage and estimate the genome size of the phage, PFGE was employed using the Fugett et al., (2007) protocol [27]. Initially, the bacteriophage contained within agarose plugs was digested with a lysis buffer, followed by an 18-h incubation at 55 °C. Subsequently, the sample was suspended in a washing buffer and then loaded into the wells of the Bio-Rad CHEF DRII system. PFGE was conducted in 0.5 X Tris-borate-EDTA at 200 V, maintaining a temperature of 14 °C for 18-h with a switch time set between 30 and 60 s. A standard catenated lambda PFG ladder (New England BioLabs, Herts, UK) ranging from 48.5 to 1,018 kb was used for comparison.

### Phage morphology using transmission electron microscopy (TEM)

The vB_Eco_ZCEC08 phage morphology was examined via TEM, at the core facility of the Medical Research Institute (Alexandria, Egypt) [28]. A sample of 10 µL of the phage (> 10^9^ PFU/mL) was stained with uranyl acetate (2.5%), then it was rinsed and negatively stained by 2% (w/v) phosphotungstic acid (pH 7.2). Subsequently, the stained solution was dried on a carbon-coated Cu-grid and prepared for visualization using TEM (1230 JEOL, Tokyo, Japan).

### Phage host range and efficiency of plating (EoP)

The lytic activity of the vB_Eco_ZCEC08 phage was assessed against 29 clinically isolated *E. coli* obtained from UTIs, along with 17 *E. coli*, as described previously [29]. Further, 10 *Salmonella enterica* strains were included in the host range analysis as a primary indication of the vB_Eco_ZCEC08 phage proximity to *Salmonella* phage vB_SenS_ST1. Initially, log-phase cultures of the different bacterial strains were used to prepare a double agar layer on TSA plates. Then, a 10-fold serial dilution of vB_Eco_ZCEC08 phage was spotted in triplicate on each of the bacterial cultures. The plates were then incubated overnight at 37 °C and observed for lysis plaques. EoP was calculated as in the following equation.

$$\:EoP=\frac{\left(\begin{aligned}&\text{T}\text{h}\text{e}\:\text{a}\text{v}\text{e}\text{r}\text{a}\text{g}\text{e}\:\text{P}\text{F}\text{U}\:\text{c}\text{o}\text{u}\text{n}\text{t}\:\text{o}\text{f}\:\text{v}\text{B}\_\text{E}\text{c}\text{o}\_\text{Z}\text{C}\text{E}\text{C}08\cr&\quad\text{p}\text{h}\text{a}\text{g}\text{e}\:\text{o}\text{n}\:\text{e}\text{a}\text{c}\text{h}\:\text{b}\text{a}\text{c}\text{t}\text{e}\text{r}\text{i}\text{a}\text{l}\:\text{h}\text{o}\text{s}\text{t}\:\end{aligned}\right)}{\left(\begin{aligned}&\text{T}\text{h}\text{e}\:\text{m}\text{a}\text{x}\text{i}\text{m}\text{u}\text{m}\:\text{P}\text{F}\text{U}\:\text{c}\text{o}\text{u}\text{n}\text{t}\cr&\quad\text{o}\text{b}\text{s}\text{e}\text{r}\text{v}\text{e}\text{d}\:\text{o}\text{n}\:\text{t}\text{h}\text{e}\:\text{m}\text{a}\text{i}\text{n}\:\text{h}\text{o}\text{s}\text{t}\left(\text{E}\text{C}-08\right)\end{aligned}\right)}$$

### Phage physicochemical stability

To study the stability of the vB_Eco_ZCEC08 phage, phage suspensions were subjected to various conditions and the residual phage titers determined after dilution in SM buffer by spotting on double-layer agar plates containing the host bacteria [30]. The stability at different physiochemical conditions is essential for the downstream applications of the phage. Firstly, the phage was exposed to different temperatures (-80, -20, 4, 37, 50, 60, 70, 75, and 80 °C) for 1 h each. Secondly, its stability was evaluated across the pH range [2–11] for 3 h. Additionally, the phage was subjected to UV exposure from a disinfectant UV lamp (~ 260 nm) for intervals (10, 20, 30, 40, 50, and 60 mintues). To confirm the safe use of chloroform during the phage purification, as in Section “Phage isolation, purification, and propagation”, the phage was tested for its stability with chloroform concentrations of 5% and 10% for (5, 15, 30, 45, and 60 min).

### Phage-bacteria replication dynamics

The dynamics of phage-bacterial replication were conducted similar to a previous study [30]. A bacterial culture (~ 10^6^ CFU/mL) was infected with vB_Eco_ZCEC08 phage at different multiplicities of infection (MOIs) of 0.1, 1, and 10. Infected and control cultures were incubated at 37 °C for 3 h. At various time intervals (0, 10, 20, 30, 45, 60, 90, 120, 150, and 180 min), the bacterial population count (control CFU/mL), bacterial survival (infected CFU/mL), and the number of phages released (PFU/mL) were determined using a 10-fold serial dilution combined with a spotting assay [31].

### Adsorption assay

An adsorption assay was undertaken to determine the time required for vB_Eco_ZCEC08 phage to attach to the bacterial host. One hundred microliters of vB_Eco_ZCEC08 phage solution (10^7^ PFU/mL) were added to 10 mL of *E. coli* (10^7^ CFU/mL) at MOI = 0.01 in a tube. Then, an aliquot was withdrawn at zero, 2, 3, 5, 7, 10, 15 and 20 min, followed by centrifugation for 16,000 x g for 1 min [32]. Then, a series of 10-fold serial dilutions were undertaken and spotted on double-layer agar containing the host bacteria.

### One-step growth curve

To study the phage growth curve, the phage titer was calculated throughout the infection cycle, as previously reported [33], with minor modifications. For instance, vB_Eco_ZCEC08 phage solution (10^8^ PFU/mL) was added to a fresh culture of *E. coli* (10^8^ CFU/mL) at MOI 0.1 and incubated at 37 ℃. Two aliquots were taken at each time point: one was treated with 3% chloroform (v:v) to induce the release of the intracellular phage virions, and the other represented the phage infective center (IC) without any treatments. Aliquots were taken at zero, 5, 6, 15, 20, 30, 50, 60, and 80 mintues, and a series of 10-fold serial dilution prepared and spotted on double-layer agar containing fresh culture of the host bacteria. The eclipse, latent, and lysis periods were determined with the burst size. The eclipse period is the time taken between phage infection (injection of the genetic material into the host bacteria) until the assembly of the virion inside the host cell. The phage latent period is the time taken from the phage infection until the first phage release, where the eclipse period is part of the latent period. The time to lysis represents the period from the first lysis of the bacteria to when the peak of the mature phage release is observed to plateau. Finally, the burst size is the average number of phages released per host cell [33].

### Phage stability in artificial human urine

Artificial human urine was prepared following the protocol of Brooks and Keevil (1997) [34] to support the growth of uropathogenic bacteria in vitro (Supplementary Table S2). Phage vB_Eco_ZCEC08 was incubated in the prepared artificial urine at 37 °C for a duration of 48 h. The phage titer was determined at different time points (0, 12, 24, and 48 h) using a 10-fold serial dilution combined with a spotting assay [31].

### Effect of artificial human urine on phage-bacteria replication dynamics

The phage-host bacterium (EC08) was washed using sterile PBS and then cultured in the artificial urine (~ 10^6^ CFU/mL). Subsequently, the bacterial culture was infected with vB_Eco_ZCEC08 phage at the optimum MOI of 1. Following the previous replication dynamics experiments, the test sample and control bacteria were then incubated at 37 °C for 3 h. Throughout this incubation period, the counts of the bacterial control (CFU/mL), bacterial survival (CFU/mL), and phage released (PFU/mL) were meticulously determined at various time intervals: 0, 30, 60, 90, 120, 150, and 180 min [20] to effectively monitor and assess the development of phage bacterial resistance in the urine environment.

### Cell viability assay

The phage effect on cell viability was tested following a previously published protocol [35]. Human Skin Fibroblast (HSF) and T-24 urinary bladder cell lines were cultured separately, in which 10^4^ cells/well were seeded in 96-well polystyrene plates. The cells were supplemented by DMEM containing 10% FBS and 100 µg penicillin/streptomycin as an antibacterial agent, then incubated at 37 °C with 5% CO_2_. Following incubation, various dilutions of vB_Eco_ZCEC08 phage (10^8^, 10^7^, and 10^6^ PFU/mL) and non-phage suspensions were independently prepared and added to the seeded cells, which were then incubated under the same growth conditions for 24 h. Subsequently, the DMEM media with different gradient concentrations was replaced by PBS containing 0.5 mg/mL MTT (3-(4,5-dimethyl-2-thiazolyl)-2,5-diphenyl-2-H-tetrazolium bromide) for 4 h at 37 °C with 5% CO_2_. The cells were imaged at 1000x magnification using an Inverted Microscope (Optika IM-3, Italy). DMSO solubilizer was added to dissolve the formazan precipitate, and the absorbance measured at 570 nm as an indicator of cell viability using a microplate reader.

### Phage DNA extraction and genome sequencing

Phage suspensions were treated with DNase1 (10 µg/mL) and RNase (10 µg/mL) before lysis in 0.5% SDS, 20 mM EDTA, 20–50 µg/mL proteinase K for 1 h at 56 °C, and extraction with phenol/ chloroform/isoamyl alcohol (25:24:1) [36]. The phage DNA was precipitated using isopropanol and sodium acetate at -20 °C overnight before redissolving in nuclease-free water. The whole genome of the phage DNA was amplified using REPLI-g (Qiagen, 150023). The amplified products were purified using 0.8X AMPure XP beads (Beckman Coulter; A63882). The concentrations of the cleaned-up samples were determined using the Qubit 4 Fluorometer (Thermo Fisher Scientific) and the Qubit 1X dsDNA HS Assay Kit (Thermo Fisher Scientific; Q33231), with 100 ng of each sample used for library preparation.

The Illumina DNA Prep Kit (Illumina; 20018705) and IDT for Illumina DNA/RNA UD Indexes Set B (Illumina; 20042666) were used to prepare indexed sequencing libraries. The library amplification process involved 5 cycles. The libraries were quantified using the Qubit 4 Fluorometer (Thermo Fisher Scientific and the Qubit 1X dsDNA HS Assay Kit (Thermo Fisher Scientific; Q33231). Agilent TapeStation 4200 and Agilent High Sensitivity D1000 ScreenTape Assay (Agilent; 5067–5584 and 5067–5585) were used to assess the library fragment-length distributions. After the libraries were combined into equivalent volumes, the KAPA Library Quantification Kit for Illumina (Roche; KK4824) was used to do the final library quantification. Using the MiSeq Reagent Nano Kit v2 (500 cycles) (Illumina; MS-103-1003), the library pool was sequenced at the University of Nottingham on an Illumina MiSeq to generate 250-bp paired-end reads.

### Bioinformatics analysis of sequencing data

#### Assembly and annotation

The genome was assembled using the de novo assembly workflow within Qiagen CLC Genomic Workbench v20.0.3. The resulting contig was used to check the similarity of the whole genome sequence on NCBI BLASTn [37]. The top hits, with the highest identity scores, were compared with the phage genome and visualized using progressiveMauve [38]. The genome was arranged using UGENE software v49.1 [39] to start with the terminase large subunit as the standard annotation starting point. The Rapid Annotation using Subsystem Technology Toolkit (RASTtk) [40] was used to align and annotate the phage genome. The annotation pipeline was customized to begin with “annotate-protein-phage,” excluding the annotation of hypothetical proteins, and then it was followed by “annotate-protein-kmer-v2”. The predicted amino acid sequences were queried against the NCBI BLASTp database [37], InterProScan [41], and HHPred [42] to confirm assigned functions and annotate hypothetical proteins. Putative transfer RNA genes (tRNA genes) were predicted using Aragorn v1.2.41 [43] and TRNAscan-SE 2.0 [44].

Regulatory regions, such as Rho-independent terminators, were determined using both ARNold [45] and Transcription Terminator Prediction web servers [46]. Moreover, the presence of *E. coli* promoter regions was detected using Prokaryote Promoter Prediction v2.0 [47]. The topology of transmembrane domains in the predicted proteins was analyzed using DeepTMHMM [48], and PEPTIDE tool v.2.0 used to check the hydrophobicity of the putative proteins [49]. Genes with potential depolymerase function were identified using Phage Depolymerase Finder (PhageDPO) [50]. The PHageBACterioPHage LIfestyle Predictor (BACPHLIP) [51] on CPT-Galaxy [52] was used to detect any temperate genetic markers. The Resistance Gene Identifier (RGI) [53] and PhageLeads tools were used to check for bacterial virulence or antimicrobial resistance-encoding genes [54]. Moreover, the PhageScope server was used additionally to confirm the annotation, presence of antimicrobial resistance or virulent factor genes, the phage taxonomy distribution, the lifestyle, transcription terminator annotation, and transmembrane protein annotation [55]. Lastly, the final annotations were utilized to generate a genomic map of the phage CGview [56] on the Proksee server [57].

#### Phylogenetic analysis

To cluster phages at the family-level classification, the ViPTree server was used to build circular and rectangular proteomic trees. The analysis comprised closely related phages and dsDNA prokaryotic viruses [58]. To construct a detailed rectangular proteomic tree, the phages with the highest ViPTree tBLASTx scores (SG) were selected. Moreover, genome-genome comparison was conducted for vB_Eco_ZCEC08 and Salmonella phage vB_SenS_ST1 using ViPTree. The virus intergenomic distance calculator (VIRIDIC) was used to assess the intergenomic similarity of the isolated phage genome to closely related phages within the same genus for a more specific classification beyond the family level. A threshold of nucleotide identity was applied to distinguish between genera (more than 70%) and species (greater than 95%) [59]. The input for VIRIDIC was reference phage genomes from ViPtree with high SG scores, top BLASTn hits with more than 60% coverage and identity, filtered reference phage genomic sequences from the NCBI virus, and an outgroup reference phage genome from other families and morphotypes from ViPtree with a low SG score. The filtration criteria were *Caudoviricetes*, sequence length between 46 and 48 kb, Refseq complete genome, and host type *Pseudomonadota*.

Furthermore, core genes detection between vB_Eco_ZCEC08 and its closely related phages within the same genus was achieved using pan-genome analysis using OrthoMCL on the KBase App [60]. Amino acid sequences from signature genes, such as terminase large subunit, were used to construct a protein-based phylogenetic tree. The protein sequences were aligned using ClustalW, along with the conserved genes of an outgroup of phages of different families and morphotypes. This previous step was performed as an attempt to verify the assignment of the isolated phage with its closest relatives. Mega 11 was used to construct phylogenetic trees, and the Maximum Likelihood approach and Whelan and Goldman model were employed to deduce the evolutionary history [61]. Using 1000 bootstrap replicates, the analysis was bolstered, and any gaps and missing data were eliminated.

PhageClouds, a protein-protein network-based method, was utilized to compare the genome of the isolated phage with other phage sequences on NCBI-GenBank. A threshold of 0.15 was employed to calculate the intergenomic distances between genomes [62].

### Statistical analysis

The experiments in this study were conducted with three biological and technical replicates. The graphs and statistical analysis were calculated using GraphPad Prism 5 software. All the statistical analysis were undertaken using One-way ANOVA to evaluate the significance set at *p* < 0.05.

## Results

### Bacterial identification

Bacteria isolated from UTIs were observed on EMB agar to display small colonies with a distinctive green-metallic sheen. Gram staining affirmed their identity as rod-shaped Gram-negative bacteria. The bacterial isolates were confirmed by PCR using *E. coli-*specific primers to produce the predicted amplicon size of 766 bp. For additional confirmation the Vitek MS automated system was employed to ascertain the identity of the phage-host bacterial strain (EC-08).

### Bacterial susceptibility to antibiotics and MAR indices

**Fig. 1:**
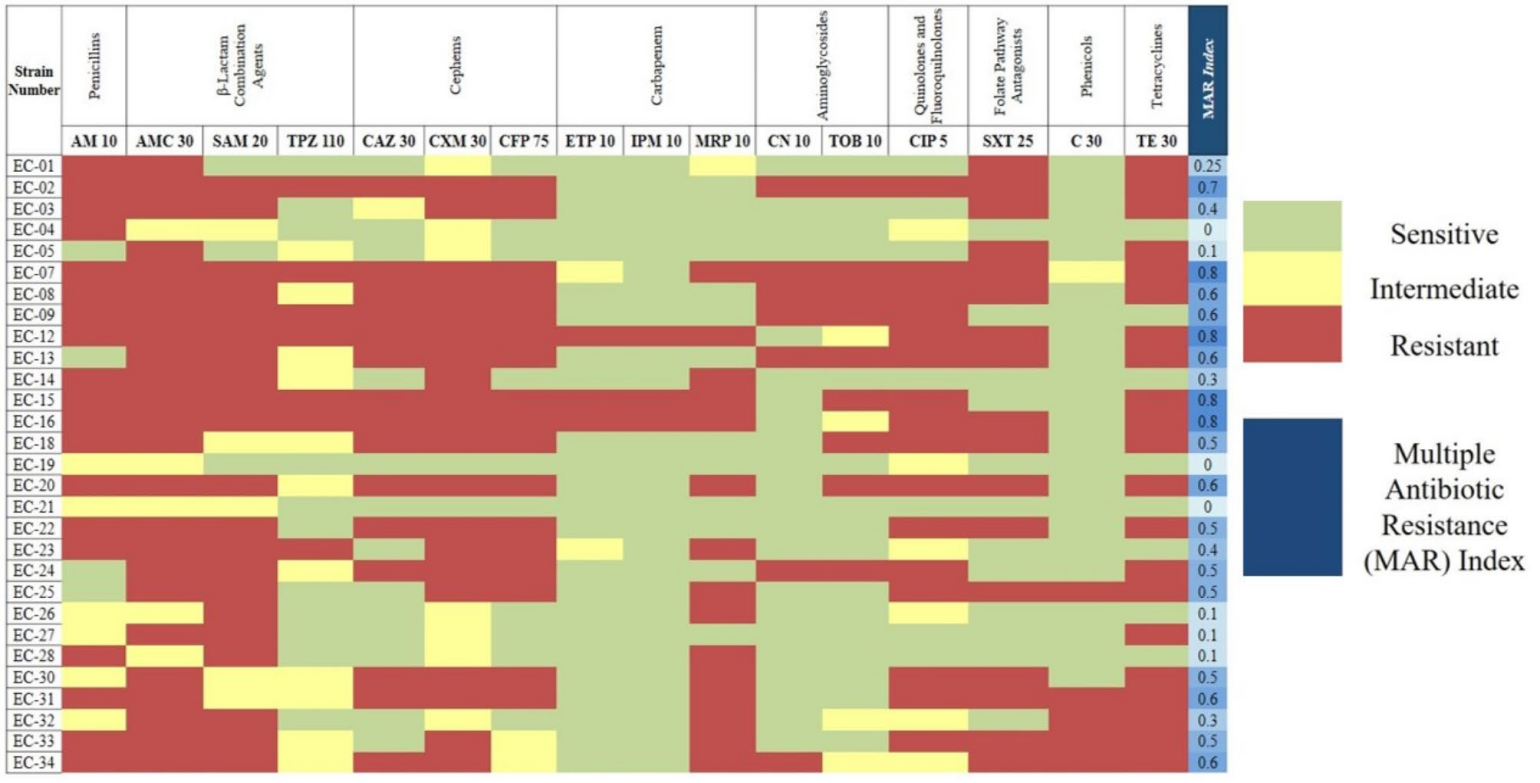
AST and MAR indices for the uropathogenic *E. coli* isolates. Each strain was tested against 16 antibiotics from 9 different classes

Out of the 29 isolates, a significant majority of 27 were classified as multidrug-resistant (MDR), indicating their non-susceptibility to at least one antimicrobial agent across three or more antimicrobial categories. Interestingly, only 2 isolates demonstrated susceptibility to the test antibiotics. The multiple antibiotic resistance (MAR) indices varied among the MDR bacteria, ranging from 0.1 to 0.8 (Fig. 1, Supplementary Table S3). Among the MDR isolates, resistance was notably observed against ampicillin, two β-lactam combination agents - amoxicillin-clavulanate and ampicillin-sulbactam, two cephems-cefuroxime and cefoperazone, as well as tetracycline. Notably, based on the antibiotic sensitivity profile, EC-08 was identified as an MDR strain with a MAR index = 0.6. EC-08 exhibited resistance to 11 antimicrobial agents from diverse classes, only showing susceptibility to the carbapenems (ertapenem, imipenem, and meropenem) and chloramphenicol among those tested.

### Bacterial virulence and antibiotic resistance

The uropathogenic *E. coli* collection showed the presence of the virulence gene *FimH* (encoding the terminal fimbrial adhesin) and less frequently *traT* (encoding a serum resistance-associated outer membrane protein) (27/34). In terms of antibiotic resistance, the extended-spectrum β-lactamase (ESBL) genes, *bla*_*CTX*_ was found to be more prevalent than *bla*_*TEM*_ (Supplementary Table S4).

### Phage morphological characterization

**Fig. 2:**
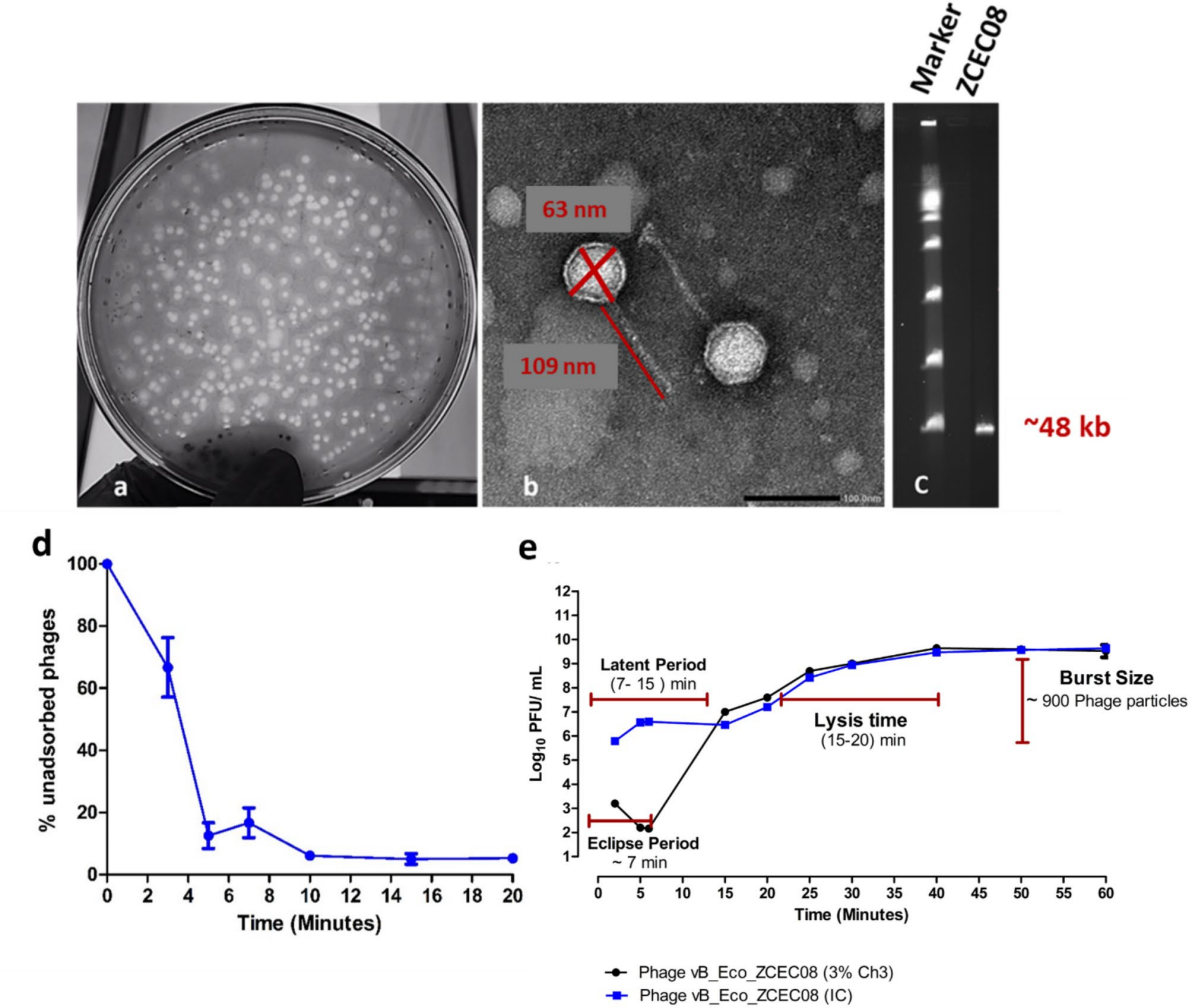
Characteristics of vB_Eco_ZCEC08 Panel (**a**) shows the plaque morphology, size, and appearance of the halo zone. Panel (**b**) shows the siphoviral morphology of vB_Eco_ZCEC08 by TEM (the image bar is 100 nm). Panel (**c**) shows phage using pulsed-field gel electrophoresis of vB_Eco_ZCEC08 with an estimated genome size of 48 kb. Panel (**d**) shows the means of triplicate adsorption assays for vB_Eco_ZCEC08 phage over 20 mintues. Panel (**e**) shows a one-step growth curve for vB_Eco_ZCEC08 at MOI 0.1

Phage vB_Eco_ZCEC08 appears on the double-layer agar plate containing the host EC-08 as clear plaques surrounded by halo of translucent zones (Fig. 2a). These halos may signify the presence of robust phage depolymerases in conjunction with the tail spike proteins [63]. TEM micrographs depicted the virion morphology as siphoviral (head with a long non-contractile tail; Fig. 2b). PFGE confirmed the phage’s purity, displaying a single genome band with a size of approximately 48 kb (Fig. 2c). Host cell adsorption showed around 50% of the phages were adsorbed after 3 mintues and 75% after 7 mintues (Fig. 2d). A one-step growth curve showed a latent period of 7 to 15 mintues, a lysis time of 15 to 20 mintues, and a burst size of approximately 900 PFU/cell (Fig. 2e).

### Phage host range and efficiency of plating

The vB_Eco_ZCEC08 phage was tested against all UPEC isolates (listed in Fig. 1), revealing that four (EC-02, EC-08, EC-13, and EC-32) exhibited susceptibility to the phage, as evidenced by the presence of a notable inhibition zone on the double-agar layer. The efficiency of plating (EoP) measurements indicated that three isolates (EC-02, EC-08, EC-13) displayed high EoP values. However, isolate EC-32 exhibited a notably low EoP value of approximately 0.000001. The host range results against further *E. coli* and *Salmonella enterica* revealed three *Salmonella* serovars were susceptible to the vB_Eco_ZCEC08 phage (Table 1).

**Table 1:** Bacterial host range for vB_Eco_ZCEC08 phage

ID/ Isotype	Bacteria	The bacteriolytic activity of vB_Eco_ZCEC08 phage
O6	*E. coli*	−
O25	*E. coli*	−
O27	*E. coli*	−
O55	*E. coli*	−
O78	*E. coli*	−
O114	*E. coli*	−
O115	*E. coli*	−
O125	*E. coli*	−
O126	*E. coli*	−
O127:H6	*E. coli*	−
O143	*E. coli*	−
O157:H7	*E. coli*	−
O161	*E. coli*	−
O164	*E. coli*	−
O168	*E. coli*	−
O169	*E. coli*	−
CMPZC49 liv	*Salmonella* enterica Blegdam III	+
CMPZC52 sple (7)	*Salmonella enterica* Blegdam IV	+
CMPZC77 int	*Salmonella enterica* Kentucky III	+
CMPZC29 int	*Salmonella enterica* Enteritidis II	−
CMPZC84 G.B	*Salmonella enterica* sp.	−
CMPZC49G.B	*Salmonella enterica* Gallinarum	−
CMPZC53 sple	*Salmonella enterica* Blegdam I	−
CMPZC18	*Salmonella enterica* sp.	−
CMPZC85 sple	*Salmonella enterica* Kentucky	−
CMPZC85 liv	*Salmonella enterica* Virchom	−

### Phage physicochemical stability

**Fig. 3:**
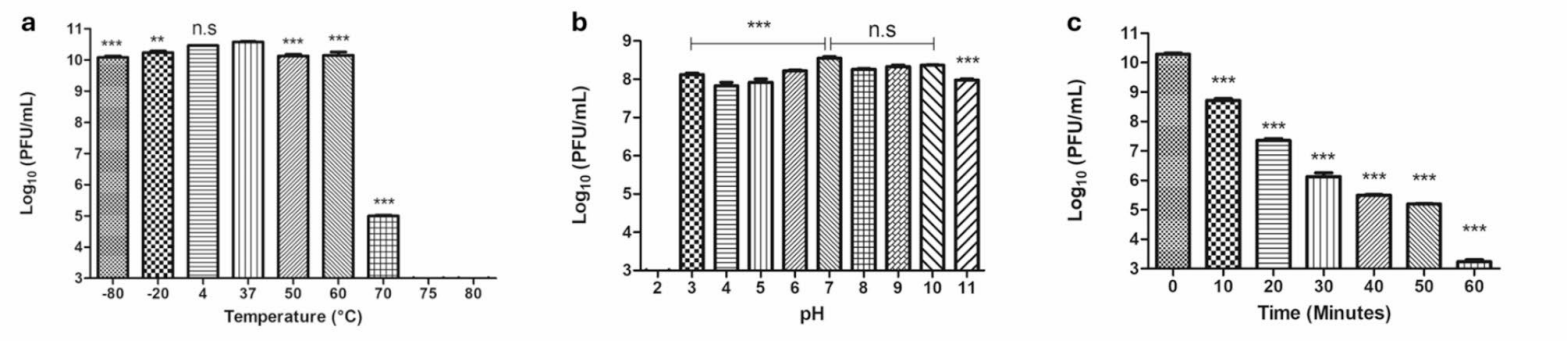
Physiochemical stability of vB_Eco_ZCEC08 phage. Panel (a) represents the temperature stability among various temperatures for 1 h. Panel (b) represents the stability of vB_Eco_ZCEC08 phage among different pH values after 3 h of incubation. Panel (c) represents the UV stability of vB_Eco_ZCEC08 phage during exposure to UV at different time points for 60 min. The bar charts show the means and standard deviations (n=3). n.s not significant *p < 0.05; **p < 0.01; ***p < 0.001

Phage vB_Eco_ZCEC08 demonstrated high stability across different physical conditions, indicating its potential suitability for different phage formulations, phage delivery and therapeutic applications. In terms of temperature, the phage exhibited robust stability over a wide range [-80–60 °C], although its activity began to decline at 70 °C and was completely lost at higher temperatures (75 and 80 °C) (Fig. 3a). Similarly, the phage displayed high stability across a broad pH range (3–11) following 3 h incubation, with activity only being compromised at pH 2 (Fig. 3b). UV stability testing revealed a gradual reduction in phage titer, averaging a 10% decrease per 10-min interval to reach approximately 30% of its original titer after 60 mintues of exposure (Fig. 3c). Moreover, the phage exhibited no significant reduction in activity following exposure to chloroform at concentrations of 5% and 10% over 60 mintues compared to the control.

### *In vitro* replication dynamics of vB_Eco_ZCEC08 phage

**Fig. 4:**
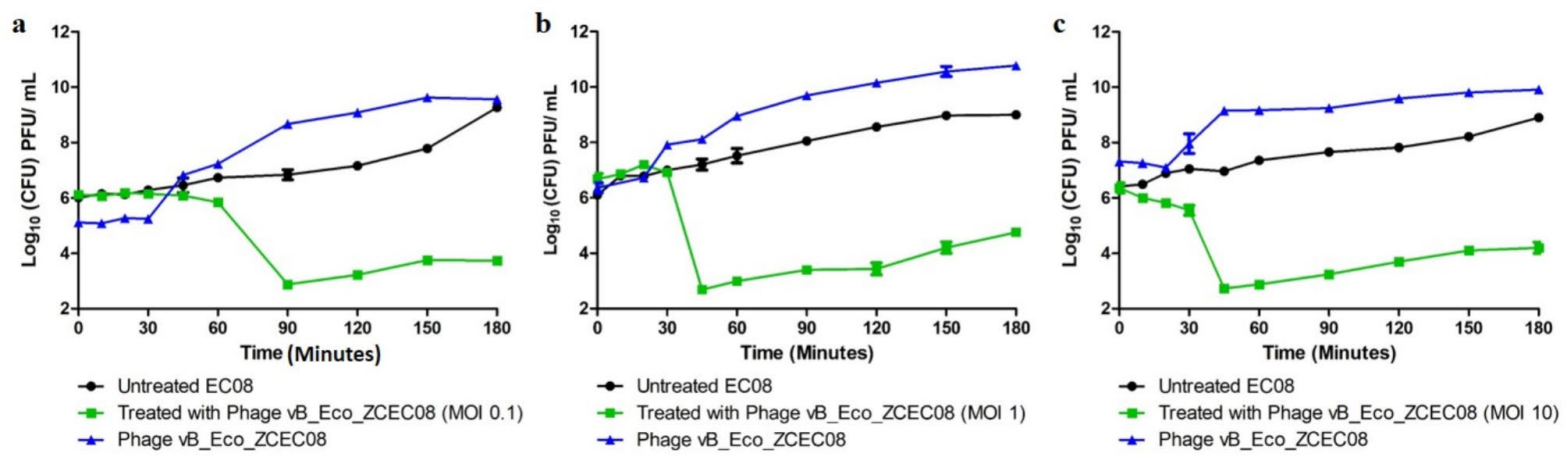
*In vitro* phage-bacteria replication dynamics at different MOIs: (a) MOI 0.1, (b) MOI 1, and (c) MOI 10 for 3 h (180 min)

Phage vB_Eco_ZCEC08 exhibited antibacterial activity against its host bacterium at different MOIs over a 3-h incubation period. Across all the MOIs tested, there was a notable reduction in bacterial count compared to untreated controls (Fig. 4). At MOI 0.1, bacterial levels dropped around 3 Log_10_ CFU/mL after 90 mintues of phage exposure. However, bacterial regrowth was observed after 120 mintues, suggesting the selection of phage-insensitive bacteria (Fig. 4a). At MOI 1, bacterial counts significantly fell by approximately 4 Log_10_ CFU/mL after 45 mintues of incubation, indicating rapid and potent antibacterial activity, although insensitive types emerged to resist the phage infection (Fig. 4b). At the highest MOI tested (MOI 10), the bacterial counts showed and initial fall within 10 min of the phage incubation before approaching a 4 Log_10_ CFU/mL reduction after 45 mintues (Fig. 4c). Phage insensitive bacteria were recovered and demonstrated to have the same antibiotic profile as the original host bacterium.

### Effect of artificial human urine on phage stability and replication dynamics

**Fig. 5:**
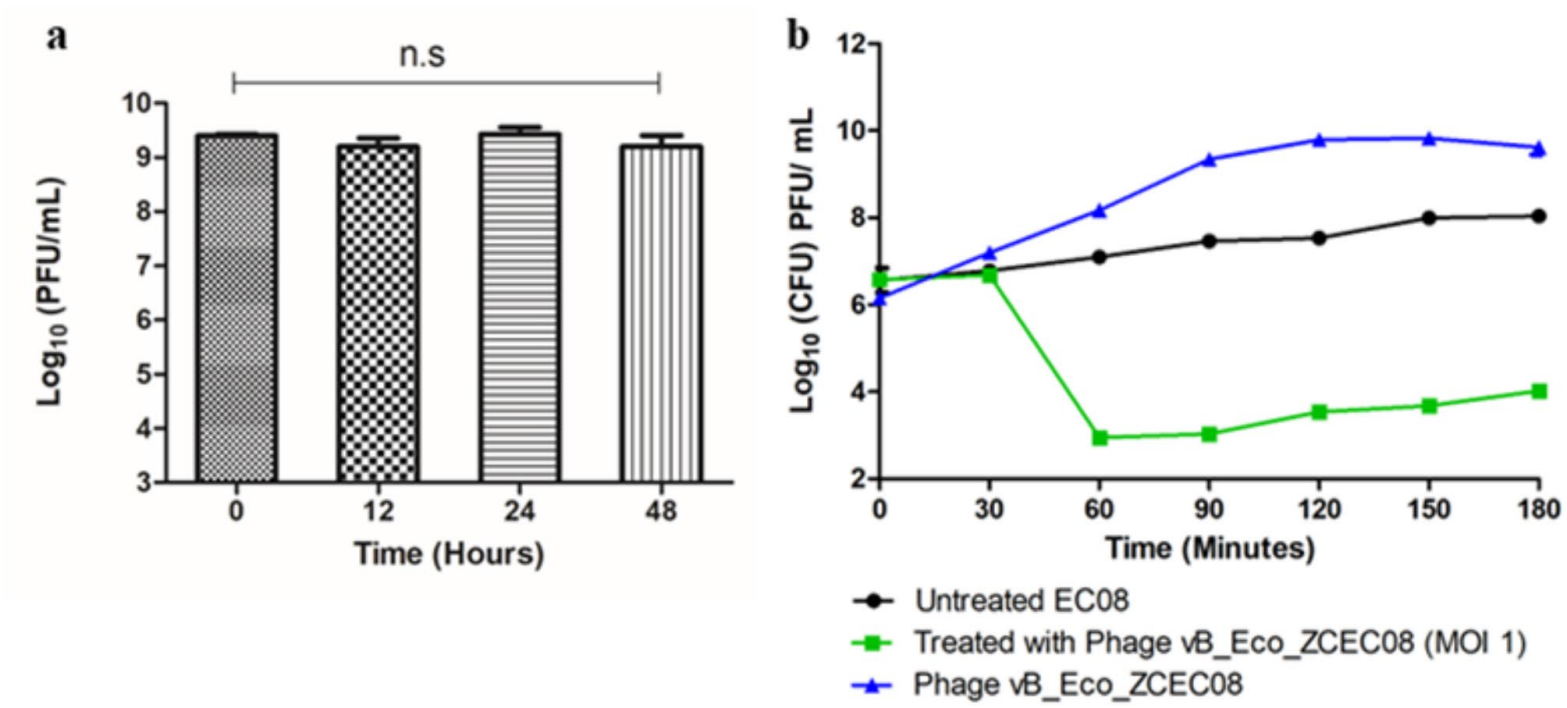
vB_Eco_ZCEC08 stability and replication in artificial human urine. Panel (a) shows the mean titers of vB_Eco_ZCEC08 phage held in artificial human urine over 48 h, where the error bars represent the standard deviation of the triplicate determinations (n.s – not significant). Panel (b) shows the phage-bacteria replication dynamics in artificial human urine at MOI= 1 over 3 h compared to non-infected control bacteria

Phage vB_Eco_ZCEC08 demonstrated robust stability in artificial human urine at 37 °C over 48 h, with no significant change in the phage titer (Fig. 5a). Given the potent activity observed at MOI = 1 *in vitro*, the phage efficacy was further investigated in artificial human urine (Fig. 5b). At MOI = 1, vB_Eco_ZCEC08 phage initiated a reduction in bacterial growth within 60 mintues of infection. However, bacterial regrowth was observed thereafter, albeit retaining an approximate 4 log_10_ CFU/mL reduction comparable to the control bacteria after 180 mintues. Phage replication was not impaired in artificial human urine, where a 3 log_10_ PFU/mL increase in the phage titer was observed over 180 mintues.

### Cell viability assay

**Fig. 6:**
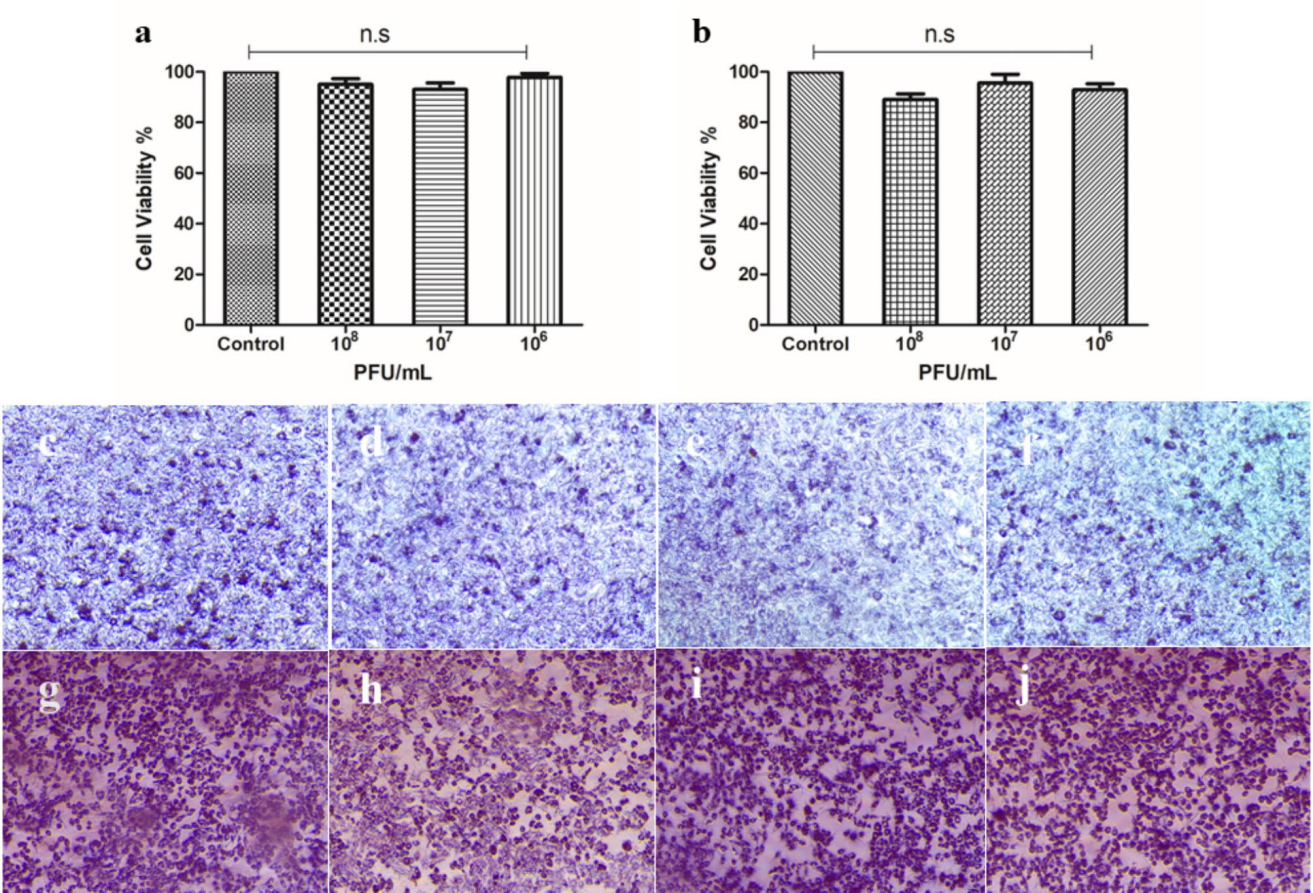
The viability of human cell lines in the presence of vB_Eco_ZCEC08. Panel (**a**) shows the means of triplicate HSF cell viability (% of control) determinations over a range of phage titers compared to control over 24 h, where the error bars represent the standard deviation of the mean % cell viability (*n* = 3; n.s – not significant). Panel (**b**) shows the effects of various phage titers on T-24 cell viability (mean % of control) over 24 h (*n* = 3; n.s – not significant). Panels (**c-j**) show photomicrographs of the cell cultures stained with MTT (**c**) the HSF cells without phage (control); panels (**d**), (**e**), and (**f**) show HSF cells exposed to 10^8^, 10^7^, and 10^6^ PFU/mL respectively; panel (**g**) shows T-24 cells without phage; panels (**h**), (**i**), and (**j**) are T-24 cells after 24 h incubation with 10^8^, 10^7^, and 10^6^ PFU/ml respectively

The vB_Eco_ZCEC08 phage exhibited likely safe use with no significant effects on the viability of normal cell lines (HSF) or bladder carcinoma cells (T-24) upon application of a range of phage titers (Fig. 6) comparable to the DMEM-only control.

### Comparative and phylogenetic analysis

#### Genomic analysis and annotation

Following DNA extraction and genome sequencing using the Illumina MiSeq platform, the sequence reads were assembled into a single contig of 47,926 bp with an average coverage of 645 reads. The average read length was 233 bp from 133,886 pair-end reads. vB_Eco_ZCEC08 phage possesses a linear double-stranded DNA genome characterized by a GC content of 46.53%. The genomic sequence of the vB_Eco_ZCEC08 phage has been submitted to the NCBI nucleotide database under accession number PP213477. Analysis with ARNold identified 35 Rho-independent terminators, and the transcription terminator prediction tool detected a further 20, 16 of which were also detected by PhageScope. A total of 16 promoters were detected, with 6 of them located on the sense strand.

**Fig. 7:**
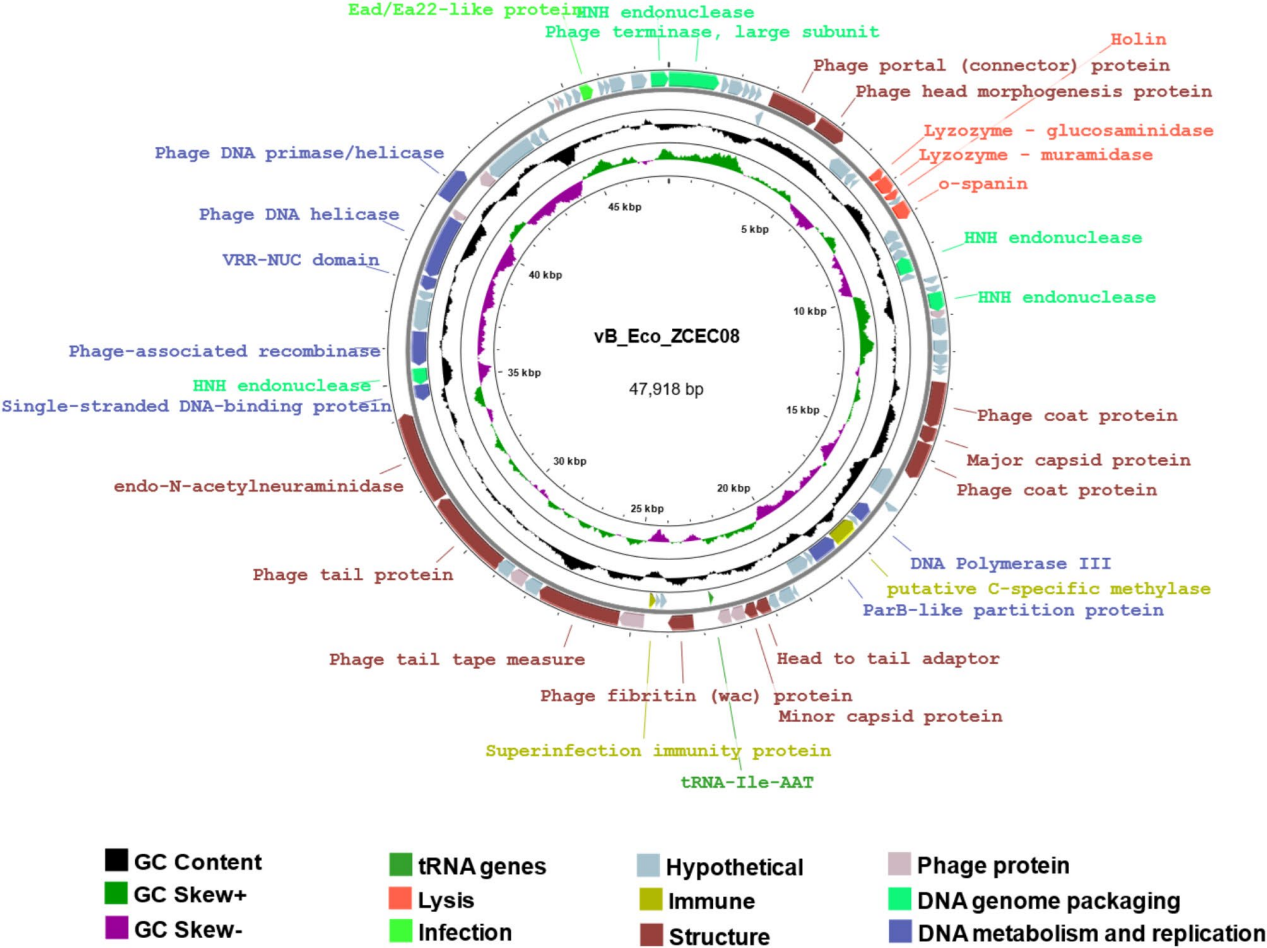
Genomic circular map of vB_Eco_ZCEC08 generated with PROKSEE. The coding sequences (CDS) are represented by different colors according to the functional group assignments: tRNA genes (dark green); lysis (salmon pink); infection (light green); immune (mustard yellow); DNA genome packaging (turquoise); structure (burgundy); DNA metabolism and replication (blue); hypothetical (grey) and phage protein (light purple). The black circle in the middle represents GC content; GC Skew + is represented in a green circle; and GC Skew – is represented in a purple inner circle

Annotation predicted 84 open reading frames (ORFs, encoding 30 proteins with assignable functions and 53 hypothetical proteins) distributed across both strands. In addition, a single tRNA gene with the anticodon AAT (Ile). The ORFs were categorized into different functional groups, encompassing structural proteins (11 genes), immune proteins (two genes), DNA genome packaging (five genes), DNA metabolism and replication proteins (seven genes), infection (one gene), and four genes associated with host cell lysis (Fig. 7, Supplementary Table S5).

The genome of phage vB_Eco_ZCEC08 encodes several structural proteins that play vital roles in phage assembly; these are largely located on the forward strand. These proteins include portal protein (ORF 8), head morphogenesis protein (ORF 9), coat proteins (ORF 32 and 34), major and minor capsid proteins (ORF 33 and 47), fibritin (ORF 50), tail tape measure protein (ORF 55), tail protein (ORF 59), and endo-N-acetyl neuraminidase (ORF 60), which were found closely clustered in groups. The genes encoding immune proteins are located on the reverse strand - a putative C-specific methylase (ORF 39) and superinfection immunity protein (ORF 53). The genes responsible for the packaging of the DNA genome include a large subunit terminase (ORF 1) and homing endonucleases (ORF 21 and 25). Functional genes involved in DNA metabolism and replication include a DNA polymerase (ORF 37), DNA binding protein (ORF 61), recombinase (ORF 63), helicase (ORF 67), and primase (ORF 69). An endo-N-acetyl neuraminidase (depolymerase) domain was predicted in the tail spike protein (ORF 60), which was supported by PhageDPO analysis with high confidence (87%, Supplementary Table S6). Genes associated with lysis functions were grouped in a cassette that included lysozymes (glucosaminidase ORF13 and muramidase, ORF14), holin (ORF 15), lysozyme, and o-spanin (ORF 17).

The amino acid sequence of the putative holin possesses two alpha-helical transmembrane domains approaching 100% probability DeepTMHMM models (Supplementary Fig. S1). The protein encoded by ORF 15 is also enriched in hydrophobic amino acids (56.58%).

The phage lifestyle was predicted as virulent with a high likelihood (96.25% and 98%) based on the respective in silico analyses BACPHLIP and PhageScope. No virulence or antibiotic resistance genes were identified by PhageLeads or RGI tools.

#### Phylogenetic analysis

**Fig. 8:**
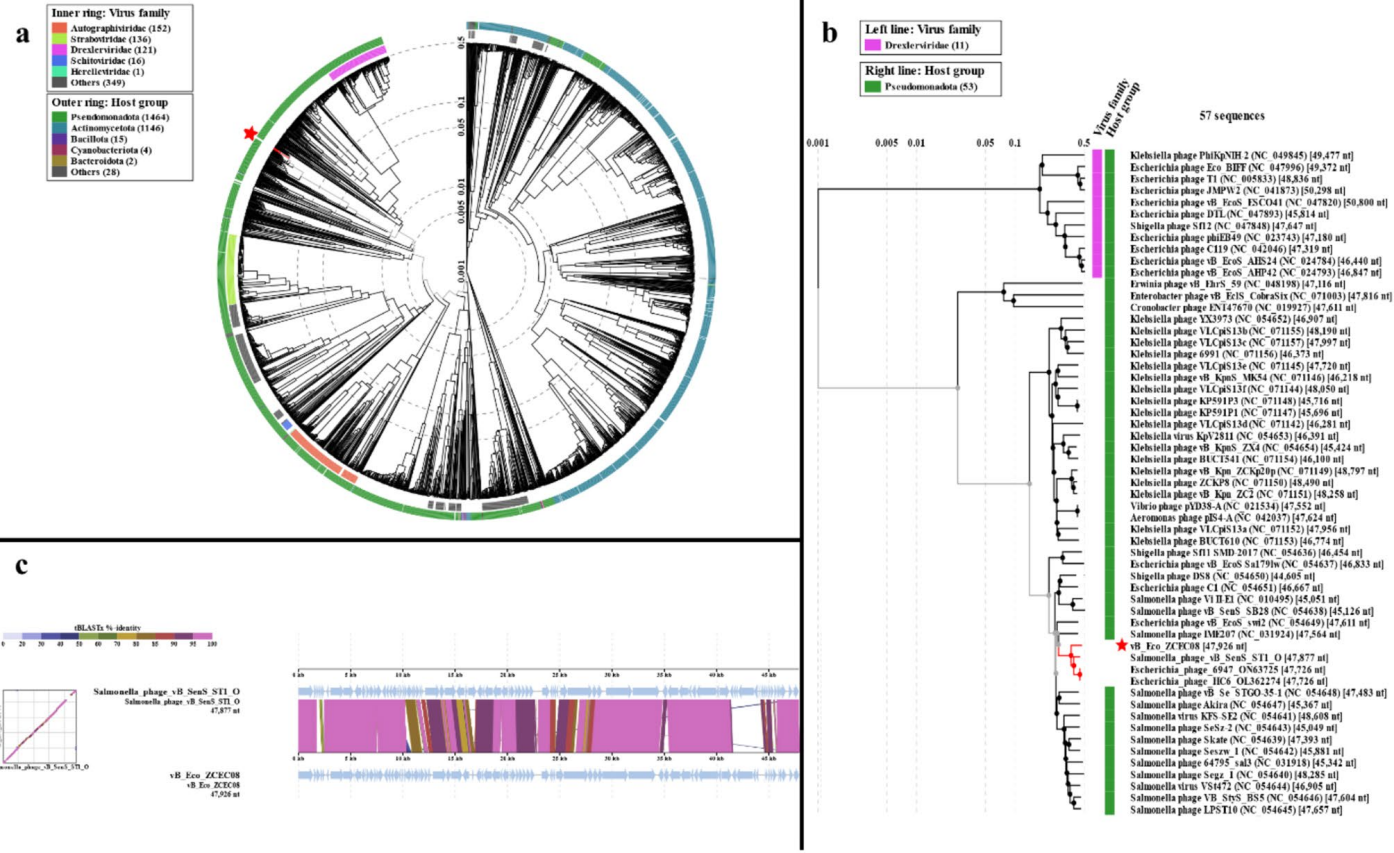
Proteomic phylogeny of vB_Eco_ZCEC08 phage (denoted with a red star). (**a**) Circular proteomic tree of phage vB_Eco_ZCEC08, clustered with top hits on BLASTn and related RefSeq phage genomes. (**b**) Rectangular proteomic tree of vB_Eco_ZCEC08 and other reference genome sequences with highest SG scores that belong to the same host group. (**c**) Genome-genome alignment of vB_Eco_ZCEC08 phage with its closest match Salmonella phage vB_SenS_ST1

The results from BLASTn conducted on NCBI revealed three phages demonstrating a notable degree of sequence identity (94–96%) and query coverage (90–92%) with the vB_Eco_ZCEC08 phage genome sequence. These phages have been categorized under unclassified *Caudoviricetes*. A circular proteomic tree was generated using VIPTree to compare vB_Eco_ZCEC08 with other reference genomes. The analysis showed that vB_Eco_ZCEC08 and related phages (*Salmonella* phage vB_SenS_ST1 - OQ469755.1, Escherichia_phage_6947 – ON637251, and Escherichia_phage_HC6 – OL362274) cluster with unclassified *Caudoviricetes* within the bacterial host class *Pseudomonadota* (Fig. 8a). Furthermore, a rectangular proteomic tree was constructed using the phage genome and the phages with the highest VipTree *S*_*G*_. Based on our analysis, phage vB_Eco_ZCEC08 formed a distinct lineage separate from the established *Drexlerviridae*, which suggests that vB_Eco_ZCEC08 might belong to a new family with its closest relative *Salmonella* phage vB_SenS_ST1 (Fig. 8b). The genome-genome alignment of vB_Eco_ZCEC08 with *Salmonella* phage vB_SenS_ST1 (OQ469755.1) is shown in Fig. 8c.

**Fig. 9:**
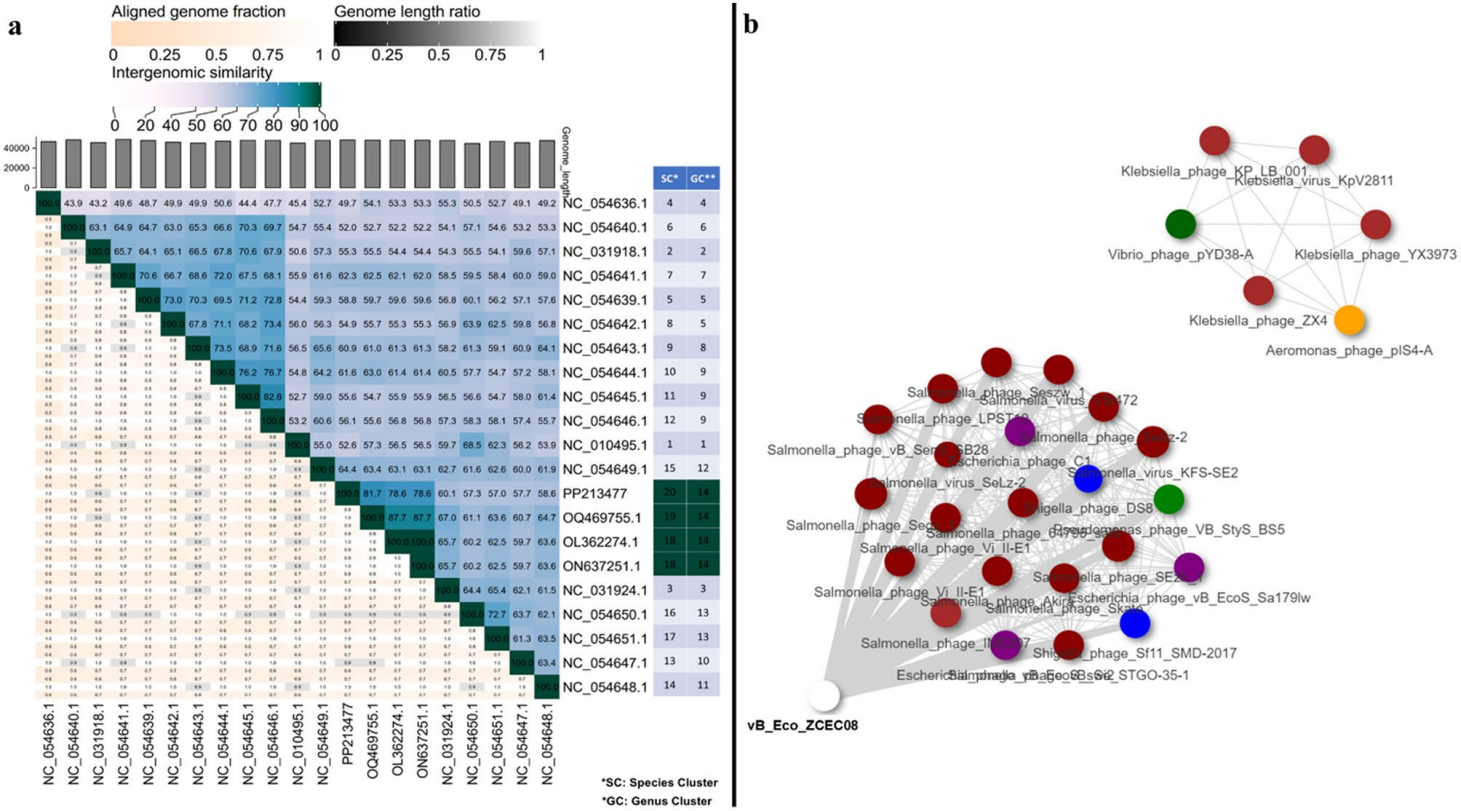
Intergenomic distance analysis of vB_Eco_ZCEC08 phage (**a**) VIRIDIC heat map of vB_Eco_ZCEC08 phage and its closest relatives from BLASTn and hits with highest tblastx scores. (**b**) PhageClouds analysis of the isolated phage in comparison with phage genomes found on NCBI-GenBank based on calculating the intergenomic distances using a threshold of 0.15

Analysis conducted using VIRIDIC aimed to establish nucleotide identity between vB_Eco_ZCEC08 and related phage genome sequences reported within nucleotide sequence databases. VIRIDC analysis takes into consideration three key factors: the aligned genome fraction, genome length ratio, and intergenomic similarity. Phages with high intergenomic similarity above 60% were shortlisted, and a heat map representing the analysis is shown in Fig. 9a. At the genus level, the analysis yielded 14 clusters, and at the species level, 20 clusters. The vB_Eco_ZCEC08 phage was grouped with other unclassified *Caudovirecetes* according to the VIRIDIC analysis (Supplementary Table S7). PhageClouds analysis grouped the isolated phage based on the calculated intergenomic distances with 22 phages of different genera, the closest of which is *Escherichia* phage vB EcoS swi2 (distance 0.1, accession MT768060) and the one with the greatest distance was *Salmonella* phage vB Sens SB28 (distance 0.14, accession MK947460) (Fig. 9b, Supplementary Table S8).

**Fig. 10:**
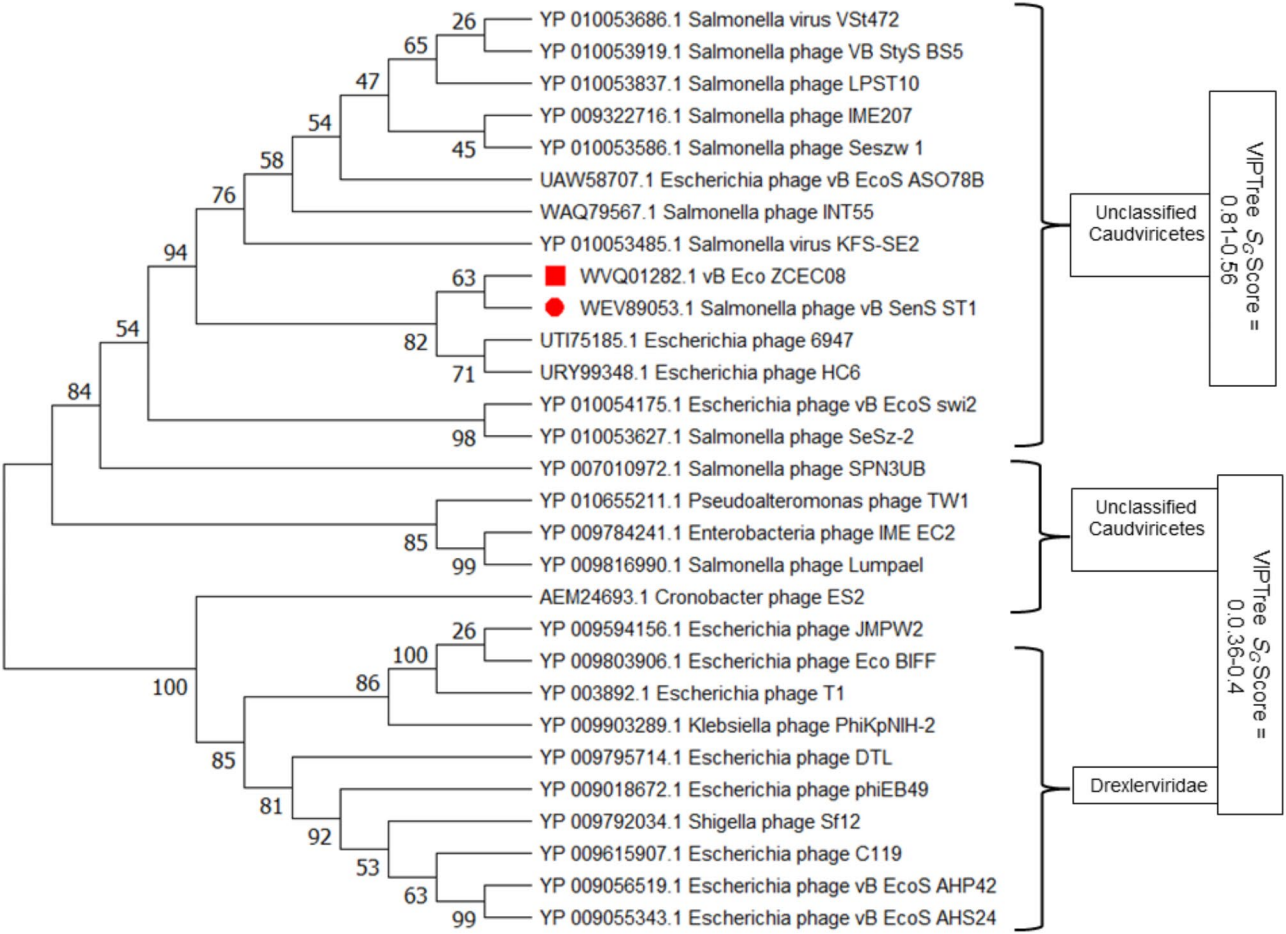
A phylogenetic tree representing the evolutionary relationship between the terminase large subunits of phage vB_Eco_ZCEC08 and the homologous proteins in the closest matches from BLASTp. The red square denotes the location of vB_Eco_ZCEC08 phage protein TerL; the red circle denotes the closest phage in the same clade

Using OrthoMCL, ~ 70 orthologous protein sequences could be identified within the predicted proteome of vB_Eco_ZCEC08 phage. These included spanin, a head morphogenesis protein, RusA-like Holliday junction resolvases, a superinfection immunity protein amongst others. These data along with the highest identity matches from BLASTn and hits from tBLASTx were used to rank the functionally conserved gene products from vB_Eco_ZCEC08 phage. The terminase large subunit (TerL) was recognized as a significant conserved protein (Supplementary Table S9). Utilizing this information, a TerL protein-based phylogenetic tree was constructed using Mega 11, which grouped vB_Eco_ZCEC08 in a single clade with *Salmonella* phage vB_SenS_ST, with which it also shares the highest genomic sequence identity (Fig. 10).

## Discussion

Uropathogenic *E. coli* stands out as the leading causative agent of both community and hospital-acquired UTIs within the *Enterobacteriaceae* family [64]. Virulent bacteriophages have emerged as promising alternatives owing to their potent and targeted bactericidal activity.

In this study, a collection of UPEC bacteria were characterized with respect to their pathogenicity potential and antibiotic sensitivity. These bacteria were used to isolate a potent lytic phage against MDR host bacteria. The phenotypic and genotypic characteristics of phage isolate vB_Eco_ZCEC08 were investigated. To mimic natural interactions between vB_Eco_ZCEC08 phage and host bacteria within the infected bladder, artificial human urine was used to explore the dynamics of phage replication under the conditions required for application, and cultured bladder cancer and normal skin fibroblast cell lines were used to assess phage cytotoxicity.

The UPEC isolates were unequivocally identified as *E. coli* via specific primers, with the phage-host strain (EC08) validated by the VITEK-MS with 99.9% accuracy, showcasing its rapid detection based on the microbial proteomic ratio. Pathogenicity and antibiotic resistance screening were investigated using two virulence genes (*fimH*, *traT*) and two antibiotic resistance genes (*bla*_*CTX*_, *bla*_*TEM*_) (Supplementary Table S4). The *fimH* gene emerged as the most prevalent among the tested UPEC isolates with 93.1%, consistent with previous findings suggesting its pivotal role in UTI pathogenesis [65]. Oliveira et al., (2011) reported that a functional *fimH* gene is essential to bacterial recognition and adherence of the bladder epithelial cells in UTIs [66]. In the current study, 75.8% of the isolates tested positive for the *traT* gene, underscoring its significance as another fundamental pathogenicity gene in the UTI collection, although less prevalent than *fimH*. These results are consistent with the findings of Rezatofighi et al., (2021), who reported that *fimH* and *traT* were the most frequently detected pathogenicity genes in UPEC isolates [65].

The presence of extended-spectrum β-lactamase (ESBL) genes, such as *bla*_*CTX*_, *bla*_*TEM*_, and *bla*_*SHV*_, encode enzymes that are able to hydrolyze a range of modified beta-lactam antibiotics to give rise to resistance [67, 68]. In our study, we focused on screening for two ESBL genes, *bla*_*CTX*_ and *bla*_*TEM*_ within the UPEC collection. The results revealed that the *bla*_*CTX*_ gene was present in 65.5% of the UPEC isolates examined, whereas the *bla*_*TEM*_ gene was detected in 44.8%. These findings align with previous research, where it was reported that the *bla*_*CTX*_ exhibited the highest prevalence at 74%, followed by *bla*_*TEM*_, at 67%, and the *bla*_*SHV*_ gene at 45% in a clinical UPEC collection comprising of 100 isolates [69].

To assess MDR, we used the MAR index score, a metric that reflects the ratio of antibiotics to which bacteria are resistant compared to the total number of antibiotics tested [25]. This index provides insights into the degree of antibiotic exposure in the environment from which the bacteria were isolated (either high, moderate, or low antibiotic use) [25]. A MAR index of ≥ 0.2 indicates MDR and suggests a high-risk source of contamination. In our study, 75.8% of the UPEC exhibited MDR characteristics with MAR indices ≥ 0.2. However, 24.2% of the isolates displayed intermediate or susceptible profiles to most of the antibiotics tested (Fig. 1). Remarkably, the phage-host bacteria (EC08) exhibited a MAR index of 0.6, signifying high-level MDR across multiple antimicrobial classes. While studies have suggested a correlation between antibiotic resistance and phage susceptibility, our findings do not support this assertion. For example, a previous study suggested that bacteria might be triggered to phage susceptibility due to the selection pressure of antibiotic resistance, indicating that bacteria with the highest MAR indices will be more susceptible to phages than low antibiotic-resistant bacteria [70]. Despite the high MAR indices observed in bacterial isolates in the current collection (EC-02 = 0.7, EC-08 = 0.6, EC-13 = 0.6, and EC-32 = 0.3), only four strains were susceptible to bacteriophage vB_Eco_ZCEC08.

The vB_Eco_ZCEC08 phage was isolated from sewage water in Giza, Egypt. Transmission electron microscopy revealed that vB_Eco_ZCEC08 is of siphoviral morphotype featuring an icosahedral head and non-contractile tail. The vB_Eco_ZCEC08 phage showed a relatively short lysis period with a high burst size of 900 PFU/cell. Moreover, it proved to be robust, exhibiting stability across a broad temperature range [-80 °C to 70 °C] after 1 h of incubation, and over a wide pH range [3–11] following 3 h of incubation. The phage also demonstrated resilience to UV exposure, maintaining stability even after 60 mintues of exposure. These findings highlight the favorable environmental stability of the vB_Eco_ZCEC08 phage, so that it can be used in different downstream formulations such as in capsules or beads for future storage and delivery. Moreover, vB_Eco_ZCEC08 phage showed higher stability compared to the characteristics of the *E. coli* phages reported in previous studies [71]. For instance, the thermal and pH stability of vB_Eco_ZCEC08 surpassed that of phages such as SKA49 and SKA64 phages reported by Sattar et al., (2023), for which stability was confined to 37 °C [72]. SKA49 was stable over a pH range from 5 to 9, whereas SKA64 activity was limited to pH 7 [72]. Additionally, the thermal stability of vB_Eco_ZCEC08 matches or exceeds that of other *E. coli* phages in our collection (ZCEC10, ZCEC11, and ZCEC12), and exhibits higher pH stability retaining titers over 3 h of incubation at pH 3 [71].

Host range analysis of vB_Eco_ZCEC08 revealed its specificity, infecting 4 *E. coli* isolates associated with UTIs, and 3 *Salmonella enterica* strains. The narrow host range, along with the absence of antibiotic resistance and virulence genes in the phage genome, suggest the phage has the potential for safe use in phage therapy, although further studies are needed to formulate for application [73]. The assessment of the cell viability in the presence of vB_Eco_ZCEC08 phage indicated no measurable cytotoxic effects on the HSF or T-24 cell lines across various phage titers (Fig. 6). These results align with findings from previous studies, which similarly reported that phages are safe and have no cytotoxic effects, thus are suitable for human therapeutic use [74, 75].

The dynamics of vB_Eco_ZCEC08 against its host bacteria were studied across different MOIs (0.1, 1, and 10) in TSB culture media, and at an MOI of 1 in artificial urine. The results indicated effective control of bacterial growth occurred across all MOIs (Figs. 4 and 5). However, bacterial regrowth was recorded, which may signify the development of phage-bacterial resistance [20]. Interestingly, the data revealed that the killing activity and the onset of resistance varied depending on the MOI applied (phage treatment dose). Specifically, MOI 10 exhibited the fastest reduction in bacterial count. On the other hand, MOI 1 displayed slower bacterial killing after 60 mintues but achieved the overall highest reduction in bacterial count. The phage produced the highest titer in the MOI 1 culture, achieving a 4 Log_10_ PFU/mL increase, suggesting the optimum phage dose that permits effective phage replication and release. EC-08 that was confirmed after spotting the phage on the bacteria recovered after regrowth. At the higher MOIs (MOI 10), the phage can lyse the bacteria without replication “Lysis from without” [76]. This might explain the relatively smaller increase in virion count observed at the end of the infection cycle when the phage was applied at an MOI of 10.

The lysis cassette of phage vB_Eco_ZCEC08 encodes lysozymes (ORFs 13 and 14), a holin (ORF 15), and an O-spanin (ORF 17) located adjacently on the genome. The ORF 15 product is a 76 amino acid hydrophobic protein that is predicted to contain two alpha-helical transmembrane domains with the N- and C-termini predicted to lie in the cytoplasm. Based on these features, and considering the gene synteny, ORF 15 aligns more closely with class II holins [77, 78].

A second annotation phase verified that ORF 60 be assigned as an endo-N-acetyl neuraminidase. The endo-N-acetyl neuraminidase enzyme has glycanase activity towards colominic acid, which can enable the formation of plaques with acapsular halos. Based on the similarity to other viral particles with host capsule depolymerase activity, the activity of this enzyme is likely linked to the tail spikes [79]. The molecular taxonomy of the vB_Eco_ZCEC08 phage was investigated using nucleotide and protein sequences. Proteomic analysis is highly dependable for determining taxonomic distant relationships of phages, especially at the family level [79, 80]. One such tool, VIPTree, constructs a proteome-based phylogeny. Using ViPTree, phage vB_Eco_ZCEC08 clustered with matches from Refseq genomes at the family level. These top matches were also identified as exhibiting the closest nucleotide identity using BLASTn analysis. Notably, this group was found to be distinct from the tailed *Drexlerviridae* family, which encompasses diverse genera such as *Eclunavirus*,* Hicfunavirus*, and *Jhansiroadvirus.* Further analysis focused on calculating the intergenomic similarities at the nucleotide level. VIRIDIC analysis clustered the phage vB_Eco_ZCEC08 with its top BLASTn matches within the same genus but could be designated as a different species. Based on PhageClouds results, vB_Eco_ZCEC08 shares a cloud with 21 other phage genomes from NCBI with distances from 0.1 to 0.14 at a threshold of 0.15. These phages belong to diverse genera, including *Macdonaldcampvirus* and *Swiduovirus* [62]. Notably, the closest BLASTn matches were excluded in the PhageClouds results since they are not designated as reference sequences in the GenBank database. The highest intergenomic distance match using PhageClouds was *Salmonella* phage vB_SenS_SB28, which belongs to the genus *Macdonaldcampvirus*, while the closest match was *Escherichia* phage vB EcoS swi2 that belongs to the genus *Swiduovirus*.

Additionally, signature gene analysis was conducted to rectify inaccuracies resulting from paired genome analyses [80]. The phylogenetic analysis of the terminase large subunit revealed that vB_Eco_ZCEC08 phage clustered within the same genus as *Salmonella* phage vB_SenS_ST1, *Escherichia* phage HC6, and *Escherichia* phage 6947. This result conformed with the clustering observed in the VIRIDIC analysis. Finally, these three phages exhibited a significant level of similarity and coverage, over 80%, when compared to the isolated phage using BLASTn. The genomic size of these organisms is approximately 48.8 Kbp, and they have around 85 genes that encode proteins. Based on the comparative information, we deduce that phage vB_Eco_ZCEC08 belongs to a distinct species, albeit it shares the same genus as *Salmonella* phage vB_SenS_ST1, *Escherichia* phage HC6, and *Escherichia* phage 6947.

## Conclusion

The virulent phage vB_Eco_ZCEC08 has emerged as a promising alternative for the control of clinical uropathogenic *E. coli*. It represents a basis for the development of a phage-based therapeutic option due to its potent lytic activity against MDR UPEC, demonstrated safety, stability under diverse environmental conditions, and the absence of cytotoxicity or undesirable genetic elements. In addition, the vB_Eco_ZCEC08 phage provides a promising model for studying bacterial resistance mechanisms for optimizing phage therapy strategies. Future research shall explore its efficacy across a broader range of UPEC serotypes and assess its synergistic potential with antimicrobials or phage cocktails to control bacterial resistance.

## Electronic supplementary material

Below is the link to the electronic supplementary material.

Supplementary Material 1

## Data Availability

All data of the current study is displayed in the original article or the supplementary materials. The vB_Eco_ZCEC08 phage whole genome sequence is deposited in the NCBI nucleotide database under accession number (PP213477).

## Abbreviations

AM: Ampicillin
AMC: Amoxicillin Clavulanate
AST: Antibiotic Sensitivity Testing
C: Chloramphenicol
CAZ: Ceftazidime
CFP: Cefoperazone
CIP: Ciprofloxacin
CN: Gentamycin
CXM: Cefuroxime
DMEM: Dulbecco’s Modified Eagle Medium
EMB: Eosin Methylene Blue
EoP: Efficiency of Plating
ESBL: Extended Spectrum β-Lactamase
ETP: Ertapenem
FBS: Fetal Bovine Serum
HSF: Human Skin Fibroblast
IPM: Imipenem
MAR: Multiple Antibiotic Resistance
MDR: Multidrug Resistant
MOI: Multiplicity of Infection
MRP: Meropenem
ORF: Open Reading Frame
PBS: Phosphate Buffer Saline
PCR: Polymerase Chain Reaction
RASTtk: Rapid Annotation using Subsystem Technology Toolkit
RGI: Resistance Gene Identifier
SAM: Ampicillin Sulbactam
SXT: Trimethoprim Sulfamethoxazole
TE: Tetracycline
TEM: Transmission Electron Microscopy
TerL: Terminase Large Subunit
TOB: Tobramycin
TPZ: Piperacillin Tazobactam
TSA: Tryptone Soy Agar
TSB: Tryptone Soy Broth
UPEC: Uropathogenic Escherichia. coli
UTI: Urinary Tract Infections
WHO: World Health Organization
