# Supplementary material for: Characterization and therapeutic potential of phage vB_Eco_ZCEC08 against multidrug-resistant uropathogenic Escherichia coli
Source: BMC Microbiol. 2025 Apr 16;25:221. doi: 10.1186/s12866-025-03903-x (PMC12001532; doi:10.1186/s12866-025-03903-x)
Supplement: Supplementary file 1 — Supplementary Material 1 [file 12866_2025_3903_MOESM1_ESM.pptx]

## Slide 1
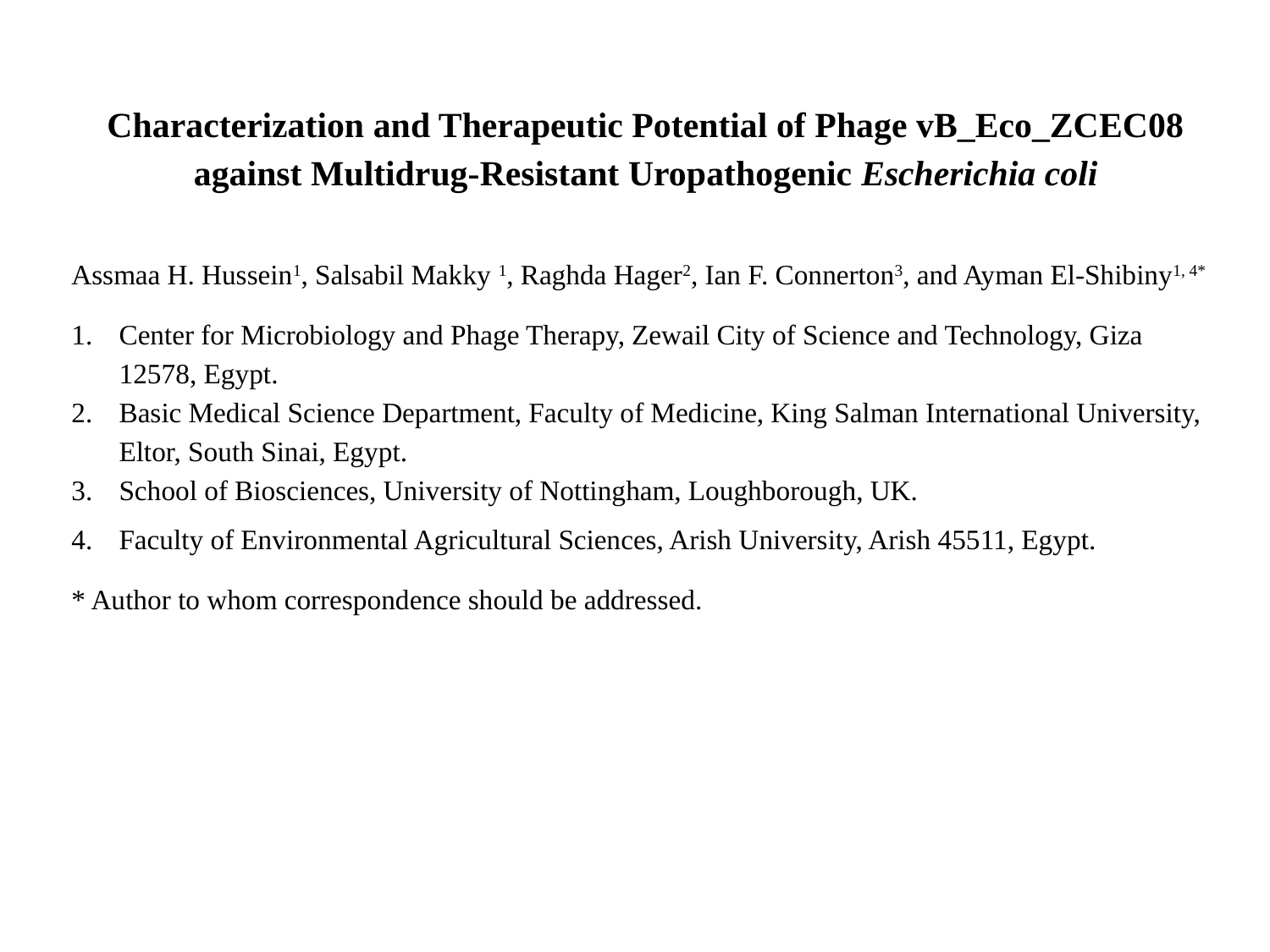

Characterization and Therapeutic Potential of Phage vB_Eco_ZCEC08 against Multidrug-Resistant Uropathogenic Escherichia coli
Assmaa H. Hussein1, Salsabil Makky 1, Raghda Hager2, Ian F. Connerton3, and Ayman El-Shibiny1, 4*
Center for Microbiology and Phage Therapy, Zewail City of Science and Technology, Giza 12578, Egypt.
Basic Medical Science Department, Faculty of Medicine, King Salman International University, Eltor, South Sinai, Egypt.
School of Biosciences, University of Nottingham, Loughborough, UK.
Faculty of Environmental Agricultural Sciences, Arish University, Arish 45511, Egypt.
* Author to whom correspondence should be addressed.

## Slide 2
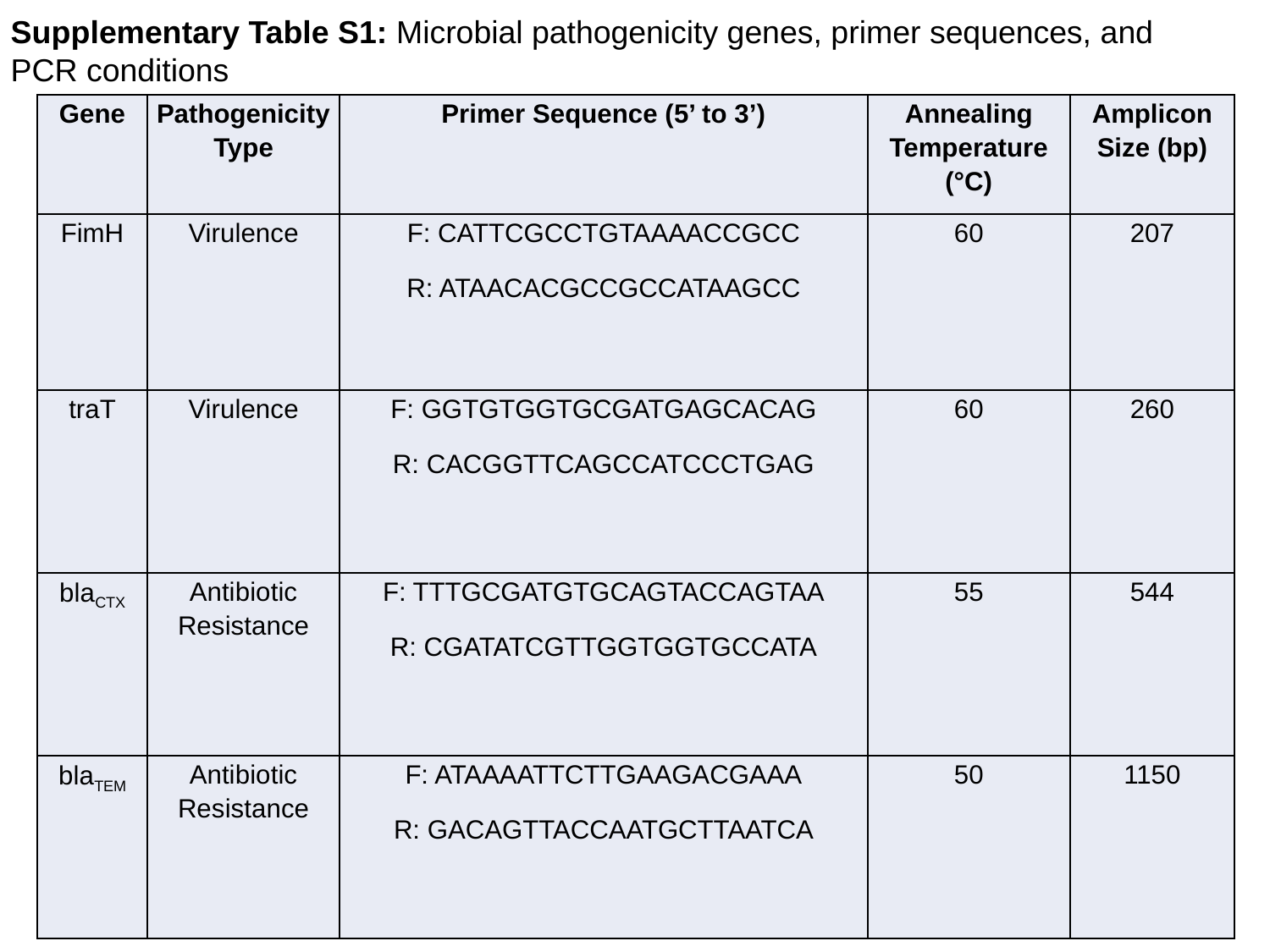

Supplementary Table S1: Microbial pathogenicity genes, primer sequences, and PCR conditions
| Gene | Pathogenicity Type | Primer Sequence (5’ to 3’) | Annealing Temperature (°C) | Amplicon Size (bp) |
| --- | --- | --- | --- | --- |
| FimH | Virulence | F: CATTCGCCTGTAAAACCGCC R: ATAACACGCCGCCATAAGCC | 60 | 207 |
| traT | Virulence | F: GGTGTGGTGCGATGAGCACAG R: CACGGTTCAGCCATCCCTGAG | 60 | 260 |
| blaCTX | Antibiotic Resistance | F: TTTGCGATGTGCAGTACCAGTAA R: CGATATCGTTGGTGGTGCCATA | 55 | 544 |
| blaTEM | Antibiotic Resistance | F: ATAAAATTCTTGAAGACGAAA R: GACAGTTACCAATGCTTAATCA | 50 | 1150 |

## Slide 3
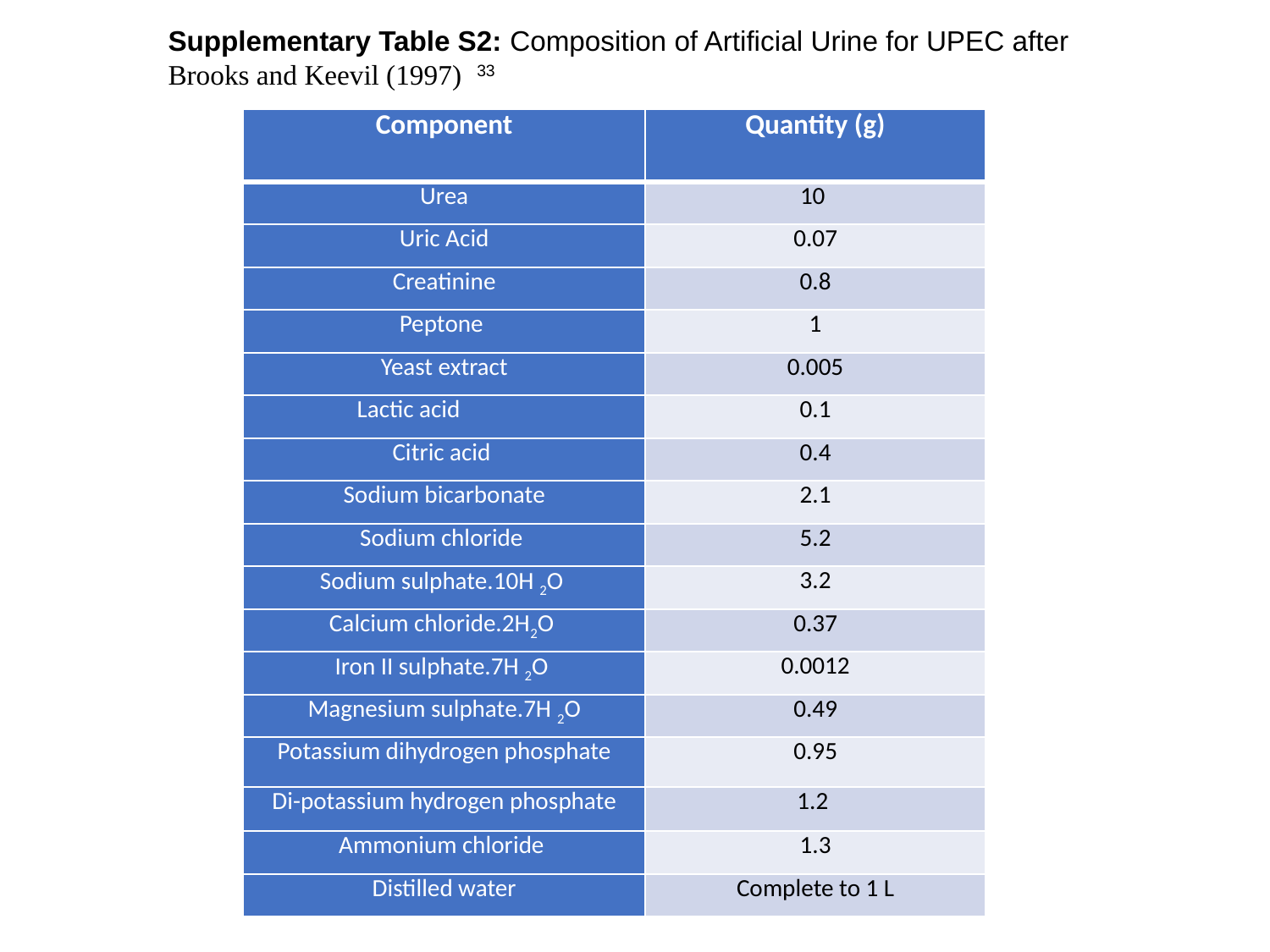

Supplementary Table S2: Composition of Artificial Urine for UPEC after Brooks and Keevil (1997) 33
| Component | Quantity (g) |
| --- | --- |
| Urea | 10 |
| Uric Acid | 0.07 |
| Creatinine | 0.8 |
| Peptone | 1 |
| Yeast extract | 0.005 |
| Lactic acid | 0.1 |
| Citric acid | 0.4 |
| Sodium bicarbonate | 2.1 |
| Sodium chloride | 5.2 |
| Sodium sulphate.10H 2O | 3.2 |
| Calcium chloride.2H2O | 0.37 |
| Iron II sulphate.7H 2O | 0.0012 |
| Magnesium sulphate.7H 2O | 0.49 |
| Potassium dihydrogen phosphate | 0.95 |
| Di-potassium hydrogen phosphate | 1.2 |
| Ammonium chloride | 1.3 |
| Distilled water | Complete to 1 L |

## Slide 4
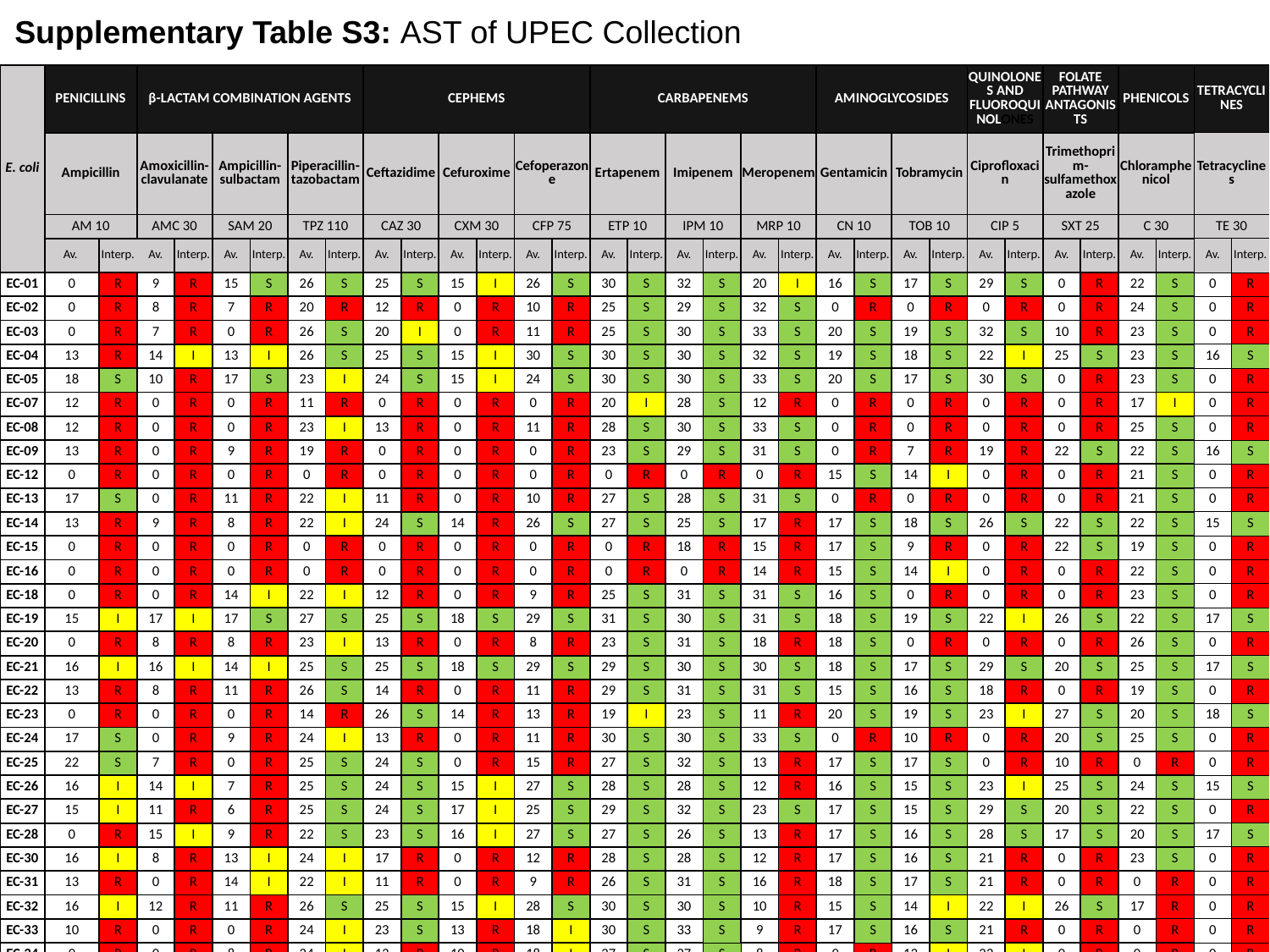

Supplementary Table S3: AST of UPEC Collection
| E. coli | PENICILLINS | | β-LACTAM COMBINATION AGENTS | | | | | | CEPHEMS | | | | | | CARBAPENEMS | | | | | | AMINOGLYCOSIDES | | | | QUINOLONES AND FLUOROQUINOLONES | | FOLATE PATHWAY ANTAGONISTS | | PHENICOLS | | TETRACYCLINES | |
| --- | --- | --- | --- | --- | --- | --- | --- | --- | --- | --- | --- | --- | --- | --- | --- | --- | --- | --- | --- | --- | --- | --- | --- | --- | --- | --- | --- | --- | --- | --- | --- | --- |
| | Ampicillin | | | | Ampicillin-sulbactam | | Piperacillin-tazobactam | | Ceftazidime | | Cefuroxime | | Cefoperazone | | Ertapenem | | Imipenem | | Meropenem | | Gentamicin | | Tobramycin | | Ciprofloxacin | | Trimethoprim-sulfamethoxazole | | Chloramphenicol | | Tetracyclines | |
| | | | Amoxicillin-clavulanate | | | | | | | | | | | | | | | | | | | | | | | | | | | | | |
| | | | | | | | | | | | | | | | | | | | | | | | | | | | | | | | | |
| | AM 10 | | AMC 30 | | SAM 20 | | TPZ 110 | | CAZ 30 | | CXM 30 | | CFP 75 | | ETP 10 | | IPM 10 | | MRP 10 | | CN 10 | | TOB 10 | | CIP 5 | | SXT 25 | | C 30 | | TE 30 | |
| | Av. | Interp. | Av. | Interp. | Av. | Interp. | Av. | Interp. | Av. | Interp. | Av. | Interp. | Av. | Interp. | Av. | Interp. | Av. | Interp. | Av. | Interp. | Av. | Interp. | Av. | Interp. | Av. | Interp. | Av. | Interp. | Av. | Interp. | Av. | Interp. |
| EC-01 | 0 | R | 9 | R | 15 | S | 26 | S | 25 | S | 15 | I | 26 | S | 30 | S | 32 | S | 20 | I | 16 | S | 17 | S | 29 | S | 0 | R | 22 | S | 0 | R |
| EC-02 | 0 | R | 8 | R | 7 | R | 20 | R | 12 | R | 0 | R | 10 | R | 25 | S | 29 | S | 32 | S | 0 | R | 0 | R | 0 | R | 0 | R | 24 | S | 0 | R |
| EC-03 | 0 | R | 7 | R | 0 | R | 26 | S | 20 | I | 0 | R | 11 | R | 25 | S | 30 | S | 33 | S | 20 | S | 19 | S | 32 | S | 10 | R | 23 | S | 0 | R |
| EC-04 | 13 | R | 14 | I | 13 | I | 26 | S | 25 | S | 15 | I | 30 | S | 30 | S | 30 | S | 32 | S | 19 | S | 18 | S | 22 | I | 25 | S | 23 | S | 16 | S |
| EC-05 | 18 | S | 10 | R | 17 | S | 23 | I | 24 | S | 15 | I | 24 | S | 30 | S | 30 | S | 33 | S | 20 | S | 17 | S | 30 | S | 0 | R | 23 | S | 0 | R |
| EC-07 | 12 | R | 0 | R | 0 | R | 11 | R | 0 | R | 0 | R | 0 | R | 20 | I | 28 | S | 12 | R | 0 | R | 0 | R | 0 | R | 0 | R | 17 | I | 0 | R |
| EC-08 | 12 | R | 0 | R | 0 | R | 23 | I | 13 | R | 0 | R | 11 | R | 28 | S | 30 | S | 33 | S | 0 | R | 0 | R | 0 | R | 0 | R | 25 | S | 0 | R |
| EC-09 | 13 | R | 0 | R | 9 | R | 19 | R | 0 | R | 0 | R | 0 | R | 23 | S | 29 | S | 31 | S | 0 | R | 7 | R | 19 | R | 22 | S | 22 | S | 16 | S |
| EC-12 | 0 | R | 0 | R | 0 | R | 0 | R | 0 | R | 0 | R | 0 | R | 0 | R | 0 | R | 0 | R | 15 | S | 14 | I | 0 | R | 0 | R | 21 | S | 0 | R |
| EC-13 | 17 | S | 0 | R | 11 | R | 22 | I | 11 | R | 0 | R | 10 | R | 27 | S | 28 | S | 31 | S | 0 | R | 0 | R | 0 | R | 0 | R | 21 | S | 0 | R |
| EC-14 | 13 | R | 9 | R | 8 | R | 22 | I | 24 | S | 14 | R | 26 | S | 27 | S | 25 | S | 17 | R | 17 | S | 18 | S | 26 | S | 22 | S | 22 | S | 15 | S |
| EC-15 | 0 | R | 0 | R | 0 | R | 0 | R | 0 | R | 0 | R | 0 | R | 0 | R | 18 | R | 15 | R | 17 | S | 9 | R | 0 | R | 22 | S | 19 | S | 0 | R |
| EC-16 | 0 | R | 0 | R | 0 | R | 0 | R | 0 | R | 0 | R | 0 | R | 0 | R | 0 | R | 14 | R | 15 | S | 14 | I | 0 | R | 0 | R | 22 | S | 0 | R |
| EC-18 | 0 | R | 0 | R | 14 | I | 22 | I | 12 | R | 0 | R | 9 | R | 25 | S | 31 | S | 31 | S | 16 | S | 0 | R | 0 | R | 0 | R | 23 | S | 0 | R |
| EC-19 | 15 | I | 17 | I | 17 | S | 27 | S | 25 | S | 18 | S | 29 | S | 31 | S | 30 | S | 31 | S | 18 | S | 19 | S | 22 | I | 26 | S | 22 | S | 17 | S |
| EC-20 | 0 | R | 8 | R | 8 | R | 23 | I | 13 | R | 0 | R | 8 | R | 23 | S | 31 | S | 18 | R | 18 | S | 0 | R | 0 | R | 0 | R | 26 | S | 0 | R |
| EC-21 | 16 | I | 16 | I | 14 | I | 25 | S | 25 | S | 18 | S | 29 | S | 29 | S | 30 | S | 30 | S | 18 | S | 17 | S | 29 | S | 20 | S | 25 | S | 17 | S |
| EC-22 | 13 | R | 8 | R | 11 | R | 26 | S | 14 | R | 0 | R | 11 | R | 29 | S | 31 | S | 31 | S | 15 | S | 16 | S | 18 | R | 0 | R | 19 | S | 0 | R |
| EC-23 | 0 | R | 0 | R | 0 | R | 14 | R | 26 | S | 14 | R | 13 | R | 19 | I | 23 | S | 11 | R | 20 | S | 19 | S | 23 | I | 27 | S | 20 | S | 18 | S |
| EC-24 | 17 | S | 0 | R | 9 | R | 24 | I | 13 | R | 0 | R | 11 | R | 30 | S | 30 | S | 33 | S | 0 | R | 10 | R | 0 | R | 20 | S | 25 | S | 0 | R |
| EC-25 | 22 | S | 7 | R | 0 | R | 25 | S | 24 | S | 0 | R | 15 | R | 27 | S | 32 | S | 13 | R | 17 | S | 17 | S | 0 | R | 10 | R | 0 | R | 0 | R |
| EC-26 | 16 | I | 14 | I | 7 | R | 25 | S | 24 | S | 15 | I | 27 | S | 28 | S | 28 | S | 12 | R | 16 | S | 15 | S | 23 | I | 25 | S | 24 | S | 15 | S |
| EC-27 | 15 | I | 11 | R | 6 | R | 25 | S | 24 | S | 17 | I | 25 | S | 29 | S | 32 | S | 23 | S | 17 | S | 15 | S | 29 | S | 20 | S | 22 | S | 0 | R |
| EC-28 | 0 | R | 15 | I | 9 | R | 22 | S | 23 | S | 16 | I | 27 | S | 27 | S | 26 | S | 13 | R | 17 | S | 16 | S | 28 | S | 17 | S | 20 | S | 17 | S |
| EC-30 | 16 | I | 8 | R | 13 | I | 24 | I | 17 | R | 0 | R | 12 | R | 28 | S | 28 | S | 12 | R | 17 | S | 16 | S | 21 | R | 0 | R | 23 | S | 0 | R |
| EC-31 | 13 | R | 0 | R | 14 | I | 22 | I | 11 | R | 0 | R | 9 | R | 26 | S | 31 | S | 16 | R | 18 | S | 17 | S | 21 | R | 0 | R | 0 | R | 0 | R |
| EC-32 | 16 | I | 12 | R | 11 | R | 26 | S | 25 | S | 15 | I | 28 | S | 30 | S | 30 | S | 10 | R | 15 | S | 14 | I | 22 | I | 26 | S | 17 | R | 0 | R |
| EC-33 | 10 | R | 0 | R | 0 | R | 24 | I | 23 | S | 13 | R | 18 | I | 30 | S | 33 | S | 9 | R | 17 | S | 16 | S | 21 | R | 0 | R | 0 | R | 0 | R |
| EC-34 | 9 | R | 0 | R | 8 | R | 24 | I | 13 | R | 10 | R | 18 | I | 27 | S | 27 | S | 8 | R | 9 | R | 13 | I | 22 | I | 0 | R | 0 | R | 0 | R |

## Slide 5
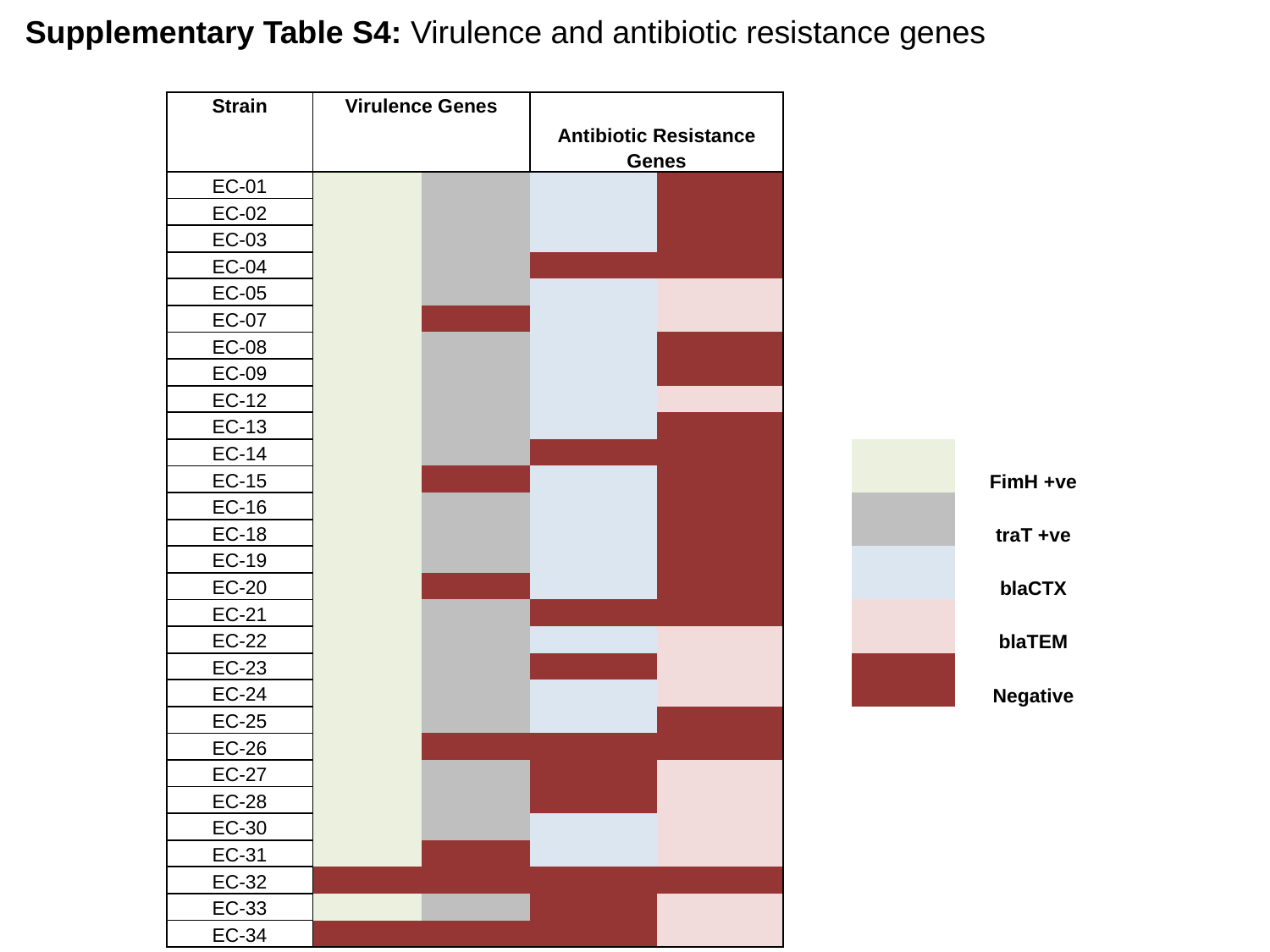

Supplementary Table S4: Virulence and antibiotic resistance genes
| Strain | Virulence Genes | | Antibiotic Resistance Genes | | | | |
| --- | --- | --- | --- | --- | --- | --- | --- |
| EC-01 | | | | | | | |
| EC-02 | | | | | | | |
| EC-03 | | | | | | | |
| EC-04 | | | | | | | |
| EC-05 | | | | | | | |
| EC-07 | | | | | | | |
| EC-08 | | | | | | | |
| EC-09 | | | | | | | |
| EC-12 | | | | | | | |
| EC-13 | | | | | | | |
| EC-14 | | | | | | | FimH +ve |
| EC-15 | | | | | | | |
| EC-16 | | | | | | | traT +ve |
| EC-18 | | | | | | | |
| EC-19 | | | | | | | blaCTX |
| EC-20 | | | | | | | |
| EC-21 | | | | | | | blaTEM |
| EC-22 | | | | | | | |
| EC-23 | | | | | | | Negative |
| EC-24 | | | | | | | |
| EC-25 | | | | | | | |
| EC-26 | | | | | | | |
| EC-27 | | | | | | | |
| EC-28 | | | | | | | |
| EC-30 | | | | | | | |
| EC-31 | | | | | | | |
| EC-32 | | | | | | | |
| EC-33 | | | | | | | |
| EC-34 | | | | | | | |

## Slide 6
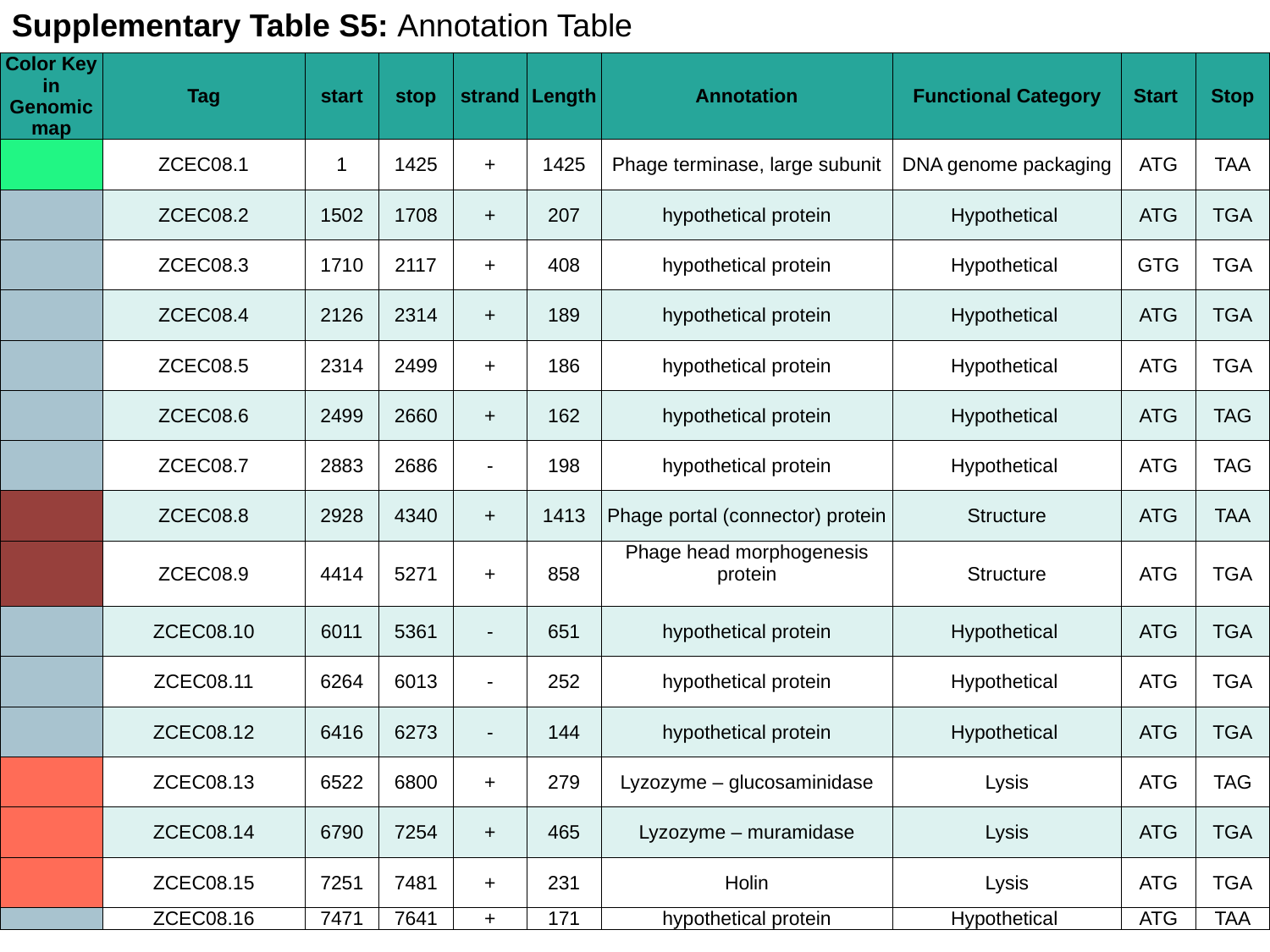

Supplementary Table S5: Annotation Table
| Color Key in Genomic map | Tag | start | stop | strand | Length | Annotation | Functional Category | Start | Stop |
| --- | --- | --- | --- | --- | --- | --- | --- | --- | --- |
| | ZCEC08.1 | 1 | 1425 | + | 1425 | Phage terminase, large subunit | DNA genome packaging | ATG | TAA |
| | ZCEC08.2 | 1502 | 1708 | + | 207 | hypothetical protein | Hypothetical | ATG | TGA |
| | ZCEC08.3 | 1710 | 2117 | + | 408 | hypothetical protein | Hypothetical | GTG | TGA |
| | ZCEC08.4 | 2126 | 2314 | + | 189 | hypothetical protein | Hypothetical | ATG | TGA |
| | ZCEC08.5 | 2314 | 2499 | + | 186 | hypothetical protein | Hypothetical | ATG | TGA |
| | ZCEC08.6 | 2499 | 2660 | + | 162 | hypothetical protein | Hypothetical | ATG | TAG |
| | ZCEC08.7 | 2883 | 2686 | - | 198 | hypothetical protein | Hypothetical | ATG | TAG |
| | ZCEC08.8 | 2928 | 4340 | + | 1413 | Phage portal (connector) protein | Structure | ATG | TAA |
| | ZCEC08.9 | 4414 | 5271 | + | 858 | Phage head morphogenesis protein | Structure | ATG | TGA |
| | ZCEC08.10 | 6011 | 5361 | - | 651 | hypothetical protein | Hypothetical | ATG | TGA |
| | ZCEC08.11 | 6264 | 6013 | - | 252 | hypothetical protein | Hypothetical | ATG | TGA |
| | ZCEC08.12 | 6416 | 6273 | - | 144 | hypothetical protein | Hypothetical | ATG | TGA |
| | ZCEC08.13 | 6522 | 6800 | + | 279 | Lyzozyme – glucosaminidase | Lysis | ATG | TAG |
| | ZCEC08.14 | 6790 | 7254 | + | 465 | Lyzozyme – muramidase | Lysis | ATG | TGA |
| | ZCEC08.15 | 7251 | 7481 | + | 231 | Holin | Lysis | ATG | TGA |
| | ZCEC08.16 | 7471 | 7641 | + | 171 | hypothetical protein | Hypothetical | ATG | TAA |

## Slide 7
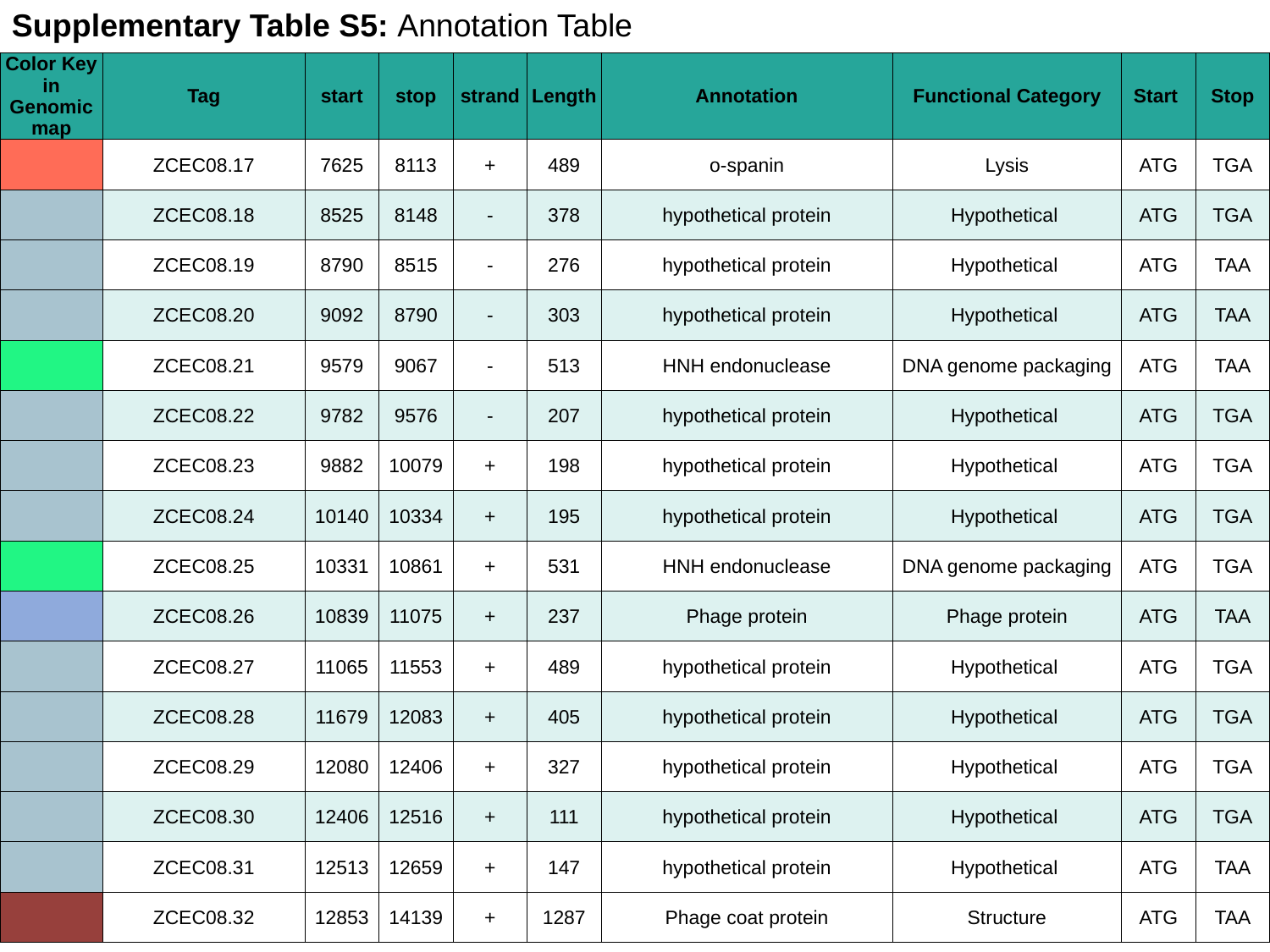

Supplementary Table S5: Annotation Table
| Color Key in Genomic map | Tag | start | stop | strand | Length | Annotation | Functional Category | Start | Stop |
| --- | --- | --- | --- | --- | --- | --- | --- | --- | --- |
| | ZCEC08.17 | 7625 | 8113 | + | 489 | o-spanin | Lysis | ATG | TGA |
| | ZCEC08.18 | 8525 | 8148 | - | 378 | hypothetical protein | Hypothetical | ATG | TGA |
| | ZCEC08.19 | 8790 | 8515 | - | 276 | hypothetical protein | Hypothetical | ATG | TAA |
| | ZCEC08.20 | 9092 | 8790 | - | 303 | hypothetical protein | Hypothetical | ATG | TAA |
| | ZCEC08.21 | 9579 | 9067 | - | 513 | HNH endonuclease | DNA genome packaging | ATG | TAA |
| | ZCEC08.22 | 9782 | 9576 | - | 207 | hypothetical protein | Hypothetical | ATG | TGA |
| | ZCEC08.23 | 9882 | 10079 | + | 198 | hypothetical protein | Hypothetical | ATG | TGA |
| | ZCEC08.24 | 10140 | 10334 | + | 195 | hypothetical protein | Hypothetical | ATG | TGA |
| | ZCEC08.25 | 10331 | 10861 | + | 531 | HNH endonuclease | DNA genome packaging | ATG | TGA |
| | ZCEC08.26 | 10839 | 11075 | + | 237 | Phage protein | Phage protein | ATG | TAA |
| | ZCEC08.27 | 11065 | 11553 | + | 489 | hypothetical protein | Hypothetical | ATG | TGA |
| | ZCEC08.28 | 11679 | 12083 | + | 405 | hypothetical protein | Hypothetical | ATG | TGA |
| | ZCEC08.29 | 12080 | 12406 | + | 327 | hypothetical protein | Hypothetical | ATG | TGA |
| | ZCEC08.30 | 12406 | 12516 | + | 111 | hypothetical protein | Hypothetical | ATG | TGA |
| | ZCEC08.31 | 12513 | 12659 | + | 147 | hypothetical protein | Hypothetical | ATG | TAA |
| | ZCEC08.32 | 12853 | 14139 | + | 1287 | Phage coat protein | Structure | ATG | TAA |

## Slide 8
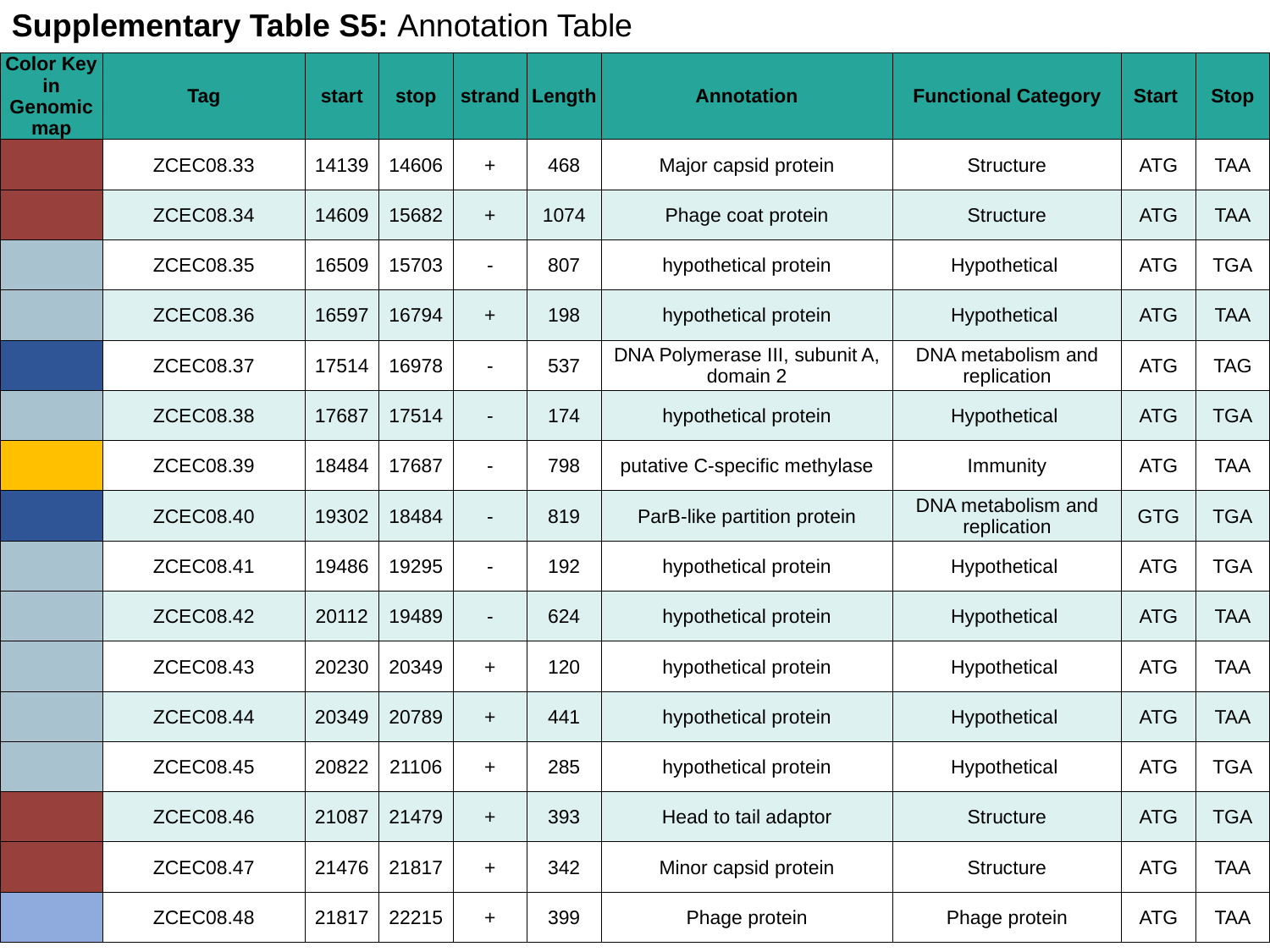

Supplementary Table S5: Annotation Table
| Color Key in Genomic map | Tag | start | stop | strand | Length | Annotation | Functional Category | Start | Stop |
| --- | --- | --- | --- | --- | --- | --- | --- | --- | --- |
| | ZCEC08.33 | 14139 | 14606 | + | 468 | Major capsid protein | Structure | ATG | TAA |
| | ZCEC08.34 | 14609 | 15682 | + | 1074 | Phage coat protein | Structure | ATG | TAA |
| | ZCEC08.35 | 16509 | 15703 | - | 807 | hypothetical protein | Hypothetical | ATG | TGA |
| | ZCEC08.36 | 16597 | 16794 | + | 198 | hypothetical protein | Hypothetical | ATG | TAA |
| | ZCEC08.37 | 17514 | 16978 | - | 537 | DNA Polymerase III, subunit A, domain 2 | DNA metabolism and replication | ATG | TAG |
| | ZCEC08.38 | 17687 | 17514 | - | 174 | hypothetical protein | Hypothetical | ATG | TGA |
| | ZCEC08.39 | 18484 | 17687 | - | 798 | putative C-specific methylase | Immunity | ATG | TAA |
| | ZCEC08.40 | 19302 | 18484 | - | 819 | ParB-like partition protein | DNA metabolism and replication | GTG | TGA |
| | ZCEC08.41 | 19486 | 19295 | - | 192 | hypothetical protein | Hypothetical | ATG | TGA |
| | ZCEC08.42 | 20112 | 19489 | - | 624 | hypothetical protein | Hypothetical | ATG | TAA |
| | ZCEC08.43 | 20230 | 20349 | + | 120 | hypothetical protein | Hypothetical | ATG | TAA |
| | ZCEC08.44 | 20349 | 20789 | + | 441 | hypothetical protein | Hypothetical | ATG | TAA |
| | ZCEC08.45 | 20822 | 21106 | + | 285 | hypothetical protein | Hypothetical | ATG | TGA |
| | ZCEC08.46 | 21087 | 21479 | + | 393 | Head to tail adaptor | Structure | ATG | TGA |
| | ZCEC08.47 | 21476 | 21817 | + | 342 | Minor capsid protein | Structure | ATG | TAA |
| | ZCEC08.48 | 21817 | 22215 | + | 399 | Phage protein | Phage protein | ATG | TAA |

## Slide 9
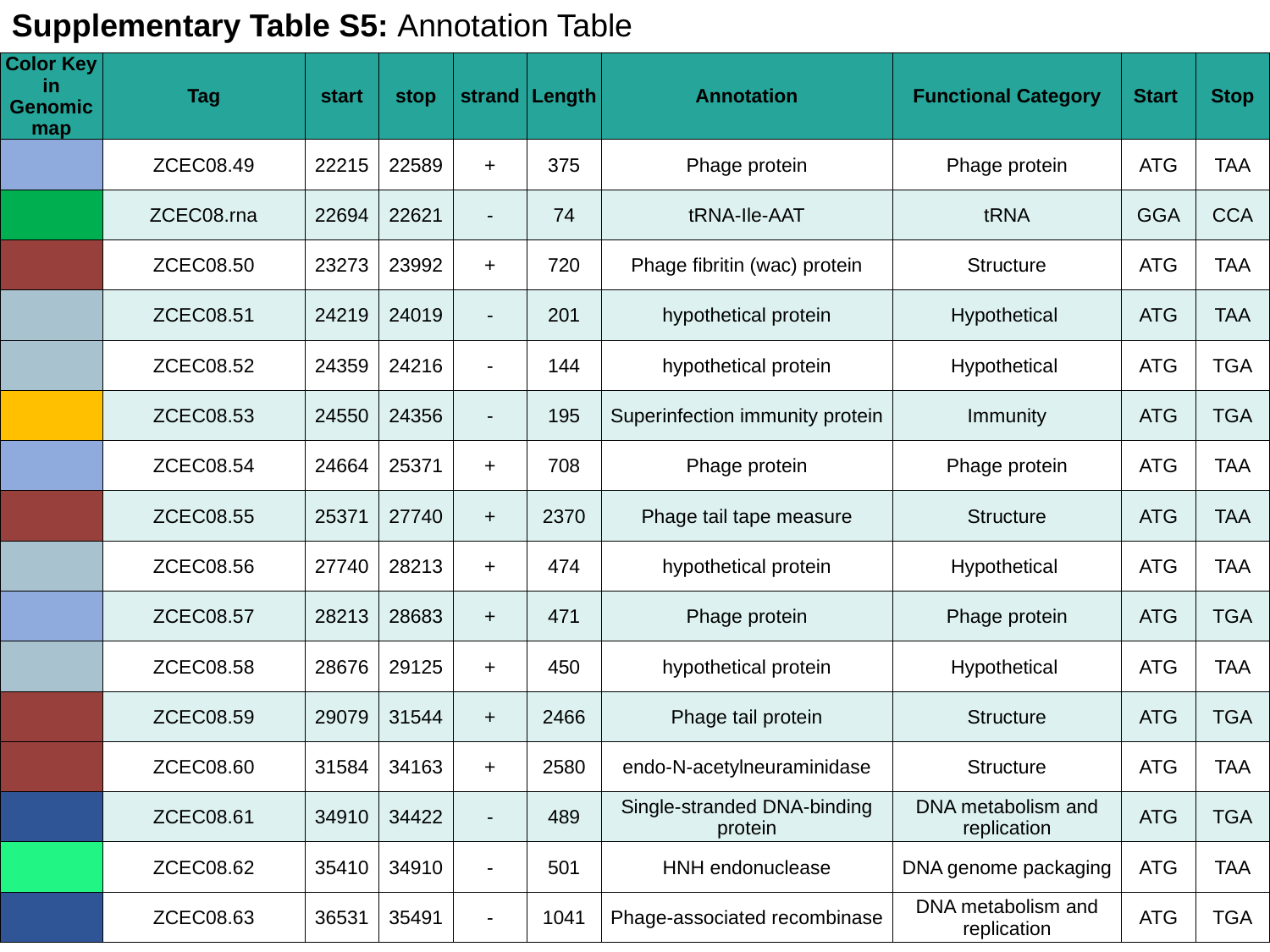

Supplementary Table S5: Annotation Table
| Color Key in Genomic map | Tag | start | stop | strand | Length | Annotation | Functional Category | Start | Stop |
| --- | --- | --- | --- | --- | --- | --- | --- | --- | --- |
| | ZCEC08.49 | 22215 | 22589 | + | 375 | Phage protein | Phage protein | ATG | TAA |
| | ZCEC08.rna | 22694 | 22621 | - | 74 | tRNA-Ile-AAT | tRNA | GGA | CCA |
| | ZCEC08.50 | 23273 | 23992 | + | 720 | Phage fibritin (wac) protein | Structure | ATG | TAA |
| | ZCEC08.51 | 24219 | 24019 | - | 201 | hypothetical protein | Hypothetical | ATG | TAA |
| | ZCEC08.52 | 24359 | 24216 | - | 144 | hypothetical protein | Hypothetical | ATG | TGA |
| | ZCEC08.53 | 24550 | 24356 | - | 195 | Superinfection immunity protein | Immunity | ATG | TGA |
| | ZCEC08.54 | 24664 | 25371 | + | 708 | Phage protein | Phage protein | ATG | TAA |
| | ZCEC08.55 | 25371 | 27740 | + | 2370 | Phage tail tape measure | Structure | ATG | TAA |
| | ZCEC08.56 | 27740 | 28213 | + | 474 | hypothetical protein | Hypothetical | ATG | TAA |
| | ZCEC08.57 | 28213 | 28683 | + | 471 | Phage protein | Phage protein | ATG | TGA |
| | ZCEC08.58 | 28676 | 29125 | + | 450 | hypothetical protein | Hypothetical | ATG | TAA |
| | ZCEC08.59 | 29079 | 31544 | + | 2466 | Phage tail protein | Structure | ATG | TGA |
| | ZCEC08.60 | 31584 | 34163 | + | 2580 | endo-N-acetylneuraminidase | Structure | ATG | TAA |
| | ZCEC08.61 | 34910 | 34422 | - | 489 | Single-stranded DNA-binding protein | DNA metabolism and replication | ATG | TGA |
| | ZCEC08.62 | 35410 | 34910 | - | 501 | HNH endonuclease | DNA genome packaging | ATG | TAA |
| | ZCEC08.63 | 36531 | 35491 | - | 1041 | Phage-associated recombinase | DNA metabolism and replication | ATG | TGA |

## Slide 10
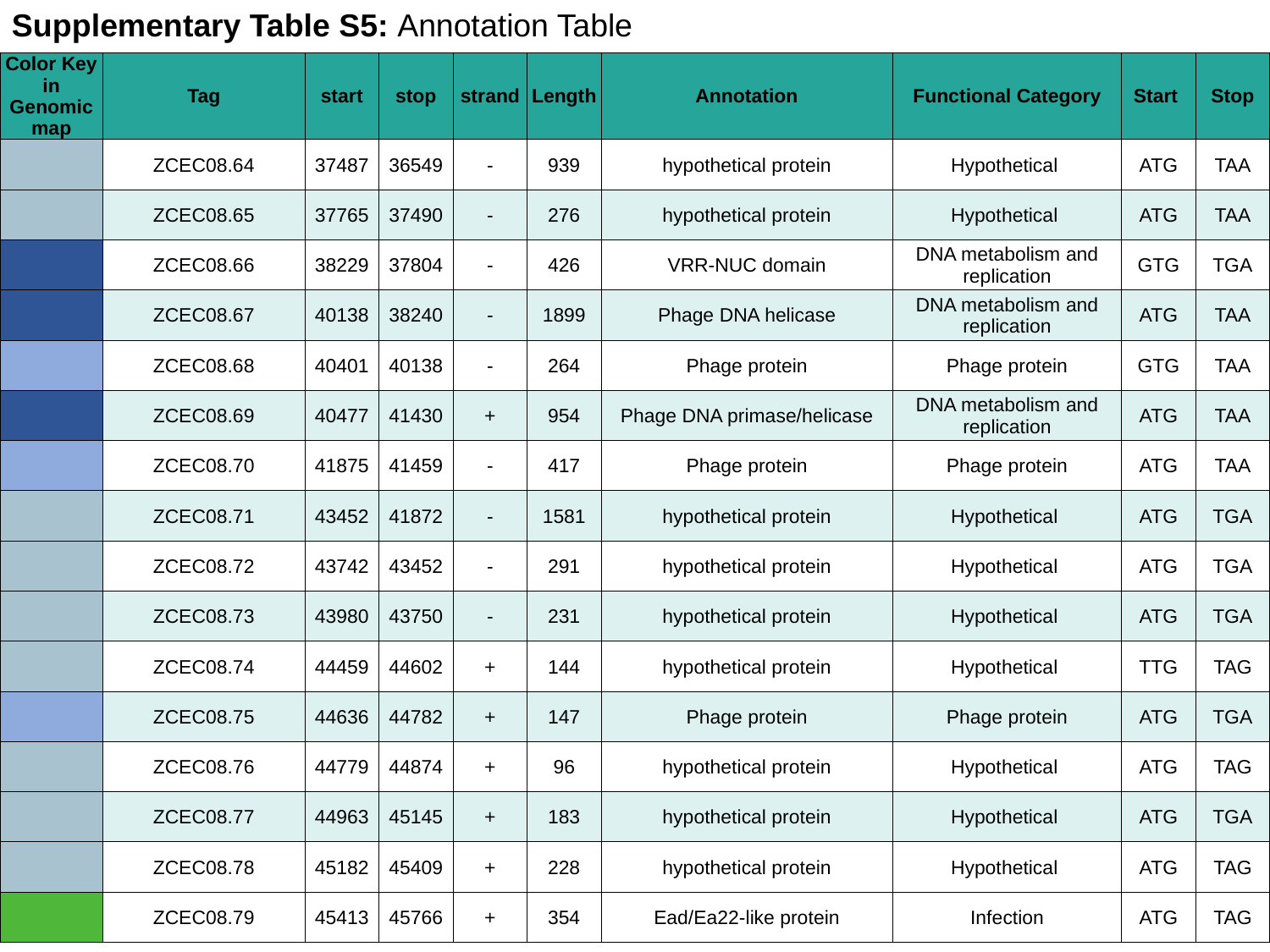

Supplementary Table S5: Annotation Table
| Color Key in Genomic map | Tag | start | stop | strand | Length | Annotation | Functional Category | Start | Stop |
| --- | --- | --- | --- | --- | --- | --- | --- | --- | --- |
| | ZCEC08.64 | 37487 | 36549 | - | 939 | hypothetical protein | Hypothetical | ATG | TAA |
| | ZCEC08.65 | 37765 | 37490 | - | 276 | hypothetical protein | Hypothetical | ATG | TAA |
| | ZCEC08.66 | 38229 | 37804 | - | 426 | VRR-NUC domain | DNA metabolism and replication | GTG | TGA |
| | ZCEC08.67 | 40138 | 38240 | - | 1899 | Phage DNA helicase | DNA metabolism and replication | ATG | TAA |
| | ZCEC08.68 | 40401 | 40138 | - | 264 | Phage protein | Phage protein | GTG | TAA |
| | ZCEC08.69 | 40477 | 41430 | + | 954 | Phage DNA primase/helicase | DNA metabolism and replication | ATG | TAA |
| | ZCEC08.70 | 41875 | 41459 | - | 417 | Phage protein | Phage protein | ATG | TAA |
| | ZCEC08.71 | 43452 | 41872 | - | 1581 | hypothetical protein | Hypothetical | ATG | TGA |
| | ZCEC08.72 | 43742 | 43452 | - | 291 | hypothetical protein | Hypothetical | ATG | TGA |
| | ZCEC08.73 | 43980 | 43750 | - | 231 | hypothetical protein | Hypothetical | ATG | TGA |
| | ZCEC08.74 | 44459 | 44602 | + | 144 | hypothetical protein | Hypothetical | TTG | TAG |
| | ZCEC08.75 | 44636 | 44782 | + | 147 | Phage protein | Phage protein | ATG | TGA |
| | ZCEC08.76 | 44779 | 44874 | + | 96 | hypothetical protein | Hypothetical | ATG | TAG |
| | ZCEC08.77 | 44963 | 45145 | + | 183 | hypothetical protein | Hypothetical | ATG | TGA |
| | ZCEC08.78 | 45182 | 45409 | + | 228 | hypothetical protein | Hypothetical | ATG | TAG |
| | ZCEC08.79 | 45413 | 45766 | + | 354 | Ead/Ea22-like protein | Infection | ATG | TAG |

## Slide 11
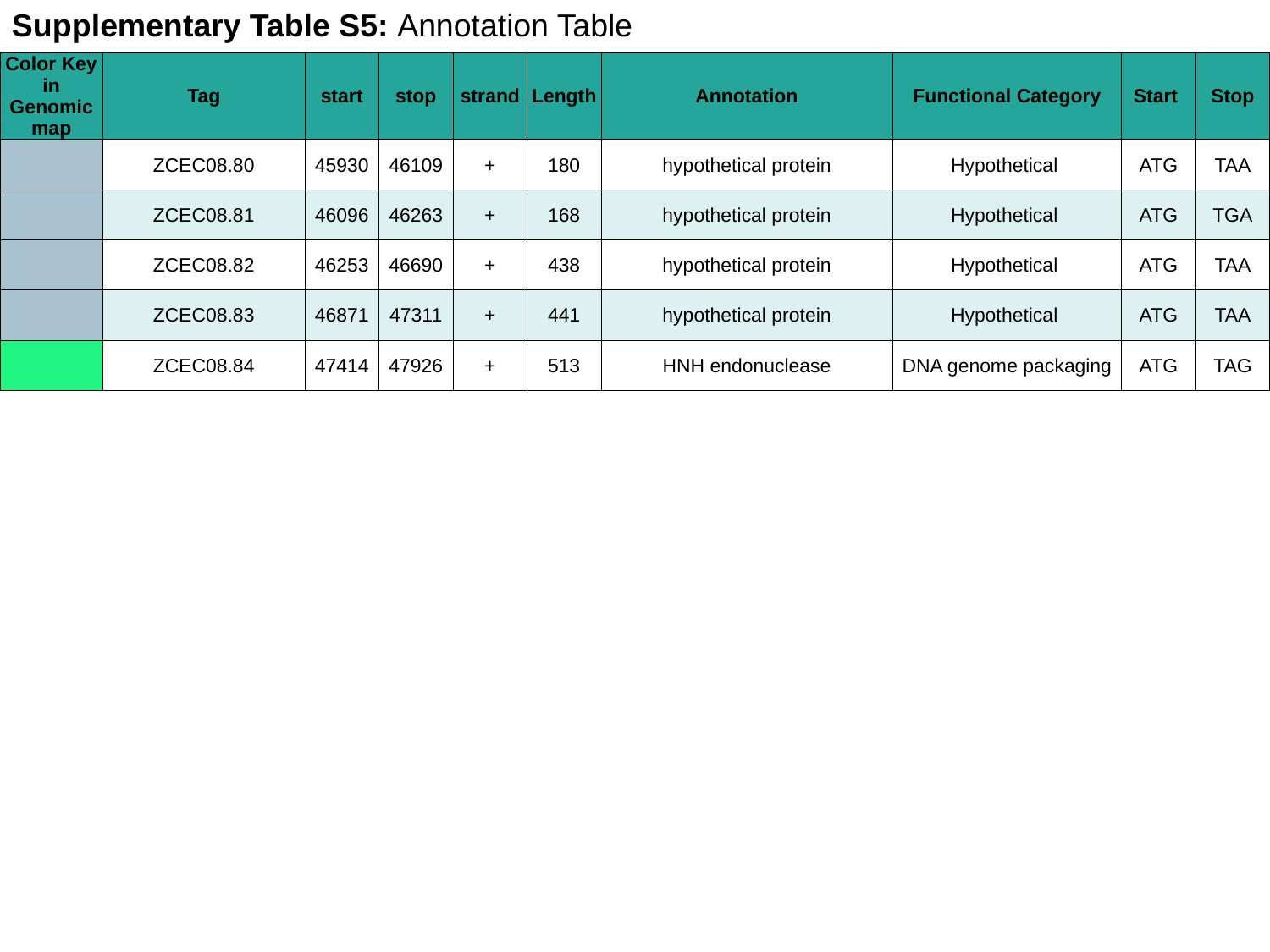

Supplementary Table S5: Annotation Table
| Color Key in Genomic map | Tag | start | stop | strand | Length | Annotation | Functional Category | Start | Stop |
| --- | --- | --- | --- | --- | --- | --- | --- | --- | --- |
| | ZCEC08.80 | 45930 | 46109 | + | 180 | hypothetical protein | Hypothetical | ATG | TAA |
| | ZCEC08.81 | 46096 | 46263 | + | 168 | hypothetical protein | Hypothetical | ATG | TGA |
| | ZCEC08.82 | 46253 | 46690 | + | 438 | hypothetical protein | Hypothetical | ATG | TAA |
| | ZCEC08.83 | 46871 | 47311 | + | 441 | hypothetical protein | Hypothetical | ATG | TAA |
| | ZCEC08.84 | 47414 | 47926 | + | 513 | HNH endonuclease | DNA genome packaging | ATG | TAG |

## Slide 12
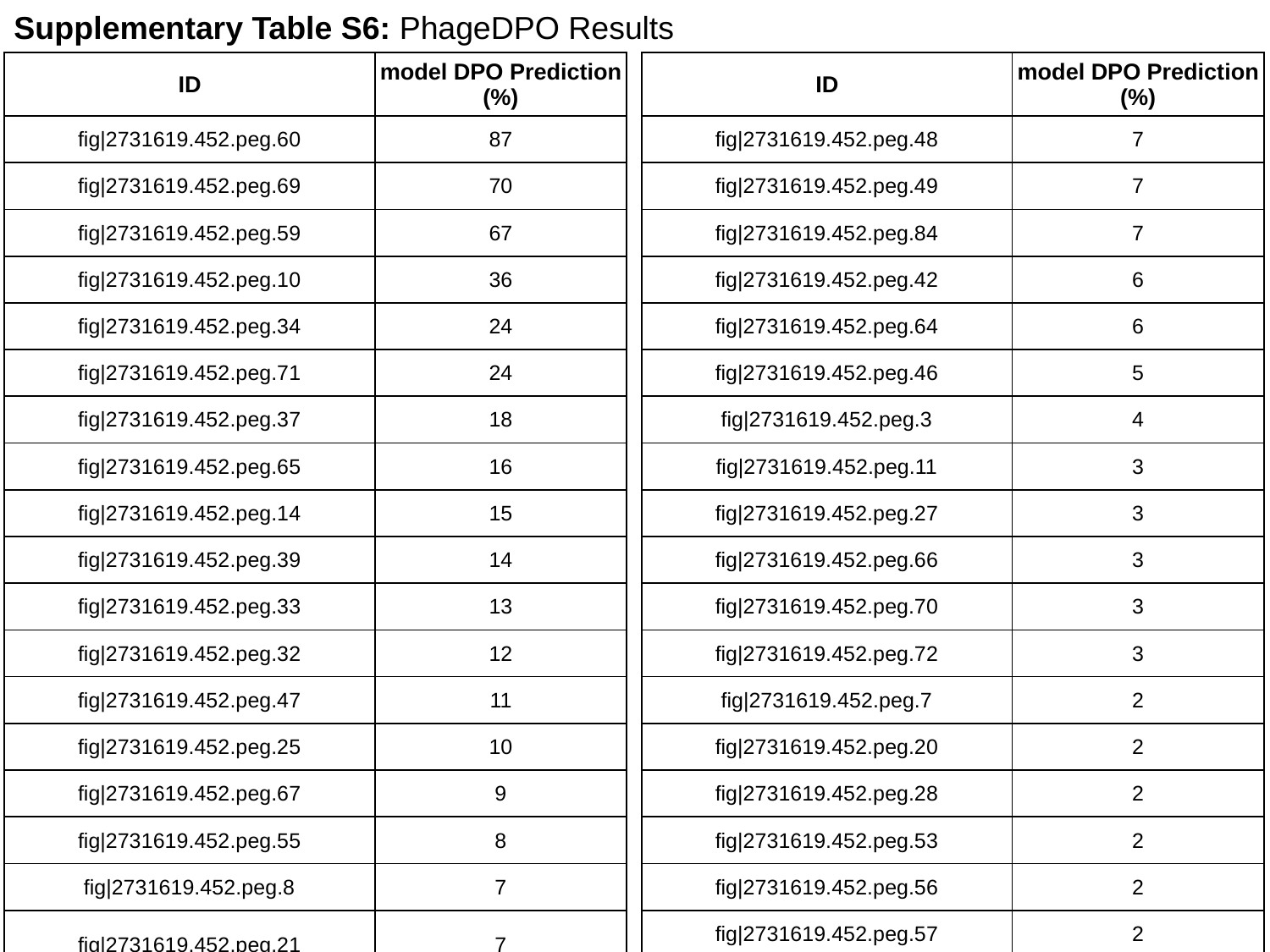

Supplementary Table S6: PhageDPO Results
| ID | model DPO Prediction (%) |
| --- | --- |
| fig|2731619.452.peg.60 | 87 |
| fig|2731619.452.peg.69 | 70 |
| fig|2731619.452.peg.59 | 67 |
| fig|2731619.452.peg.10 | 36 |
| fig|2731619.452.peg.34 | 24 |
| fig|2731619.452.peg.71 | 24 |
| fig|2731619.452.peg.37 | 18 |
| fig|2731619.452.peg.65 | 16 |
| fig|2731619.452.peg.14 | 15 |
| fig|2731619.452.peg.39 | 14 |
| fig|2731619.452.peg.33 | 13 |
| fig|2731619.452.peg.32 | 12 |
| fig|2731619.452.peg.47 | 11 |
| fig|2731619.452.peg.25 | 10 |
| fig|2731619.452.peg.67 | 9 |
| fig|2731619.452.peg.55 | 8 |
| fig|2731619.452.peg.8 | 7 |
| fig|2731619.452.peg.21 | 7 |
| ID | model DPO Prediction (%) |
| --- | --- |
| fig|2731619.452.peg.48 | 7 |
| fig|2731619.452.peg.49 | 7 |
| fig|2731619.452.peg.84 | 7 |
| fig|2731619.452.peg.42 | 6 |
| fig|2731619.452.peg.64 | 6 |
| fig|2731619.452.peg.46 | 5 |
| fig|2731619.452.peg.3 | 4 |
| fig|2731619.452.peg.11 | 3 |
| fig|2731619.452.peg.27 | 3 |
| fig|2731619.452.peg.66 | 3 |
| fig|2731619.452.peg.70 | 3 |
| fig|2731619.452.peg.72 | 3 |
| fig|2731619.452.peg.7 | 2 |
| fig|2731619.452.peg.20 | 2 |
| fig|2731619.452.peg.28 | 2 |
| fig|2731619.452.peg.53 | 2 |
| fig|2731619.452.peg.56 | 2 |
| fig|2731619.452.peg.57 | 2 |

## Slide 13
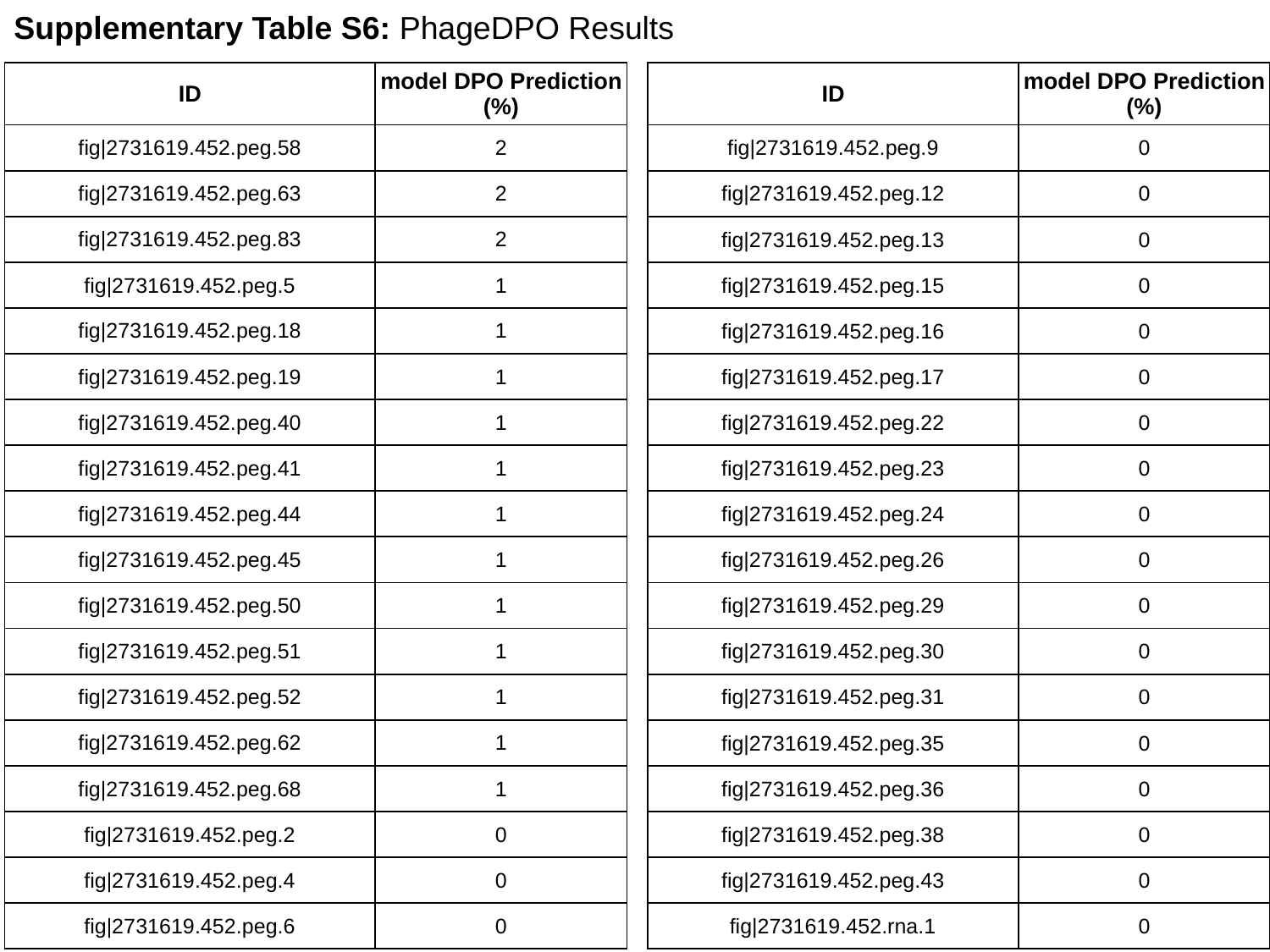

Supplementary Table S6: PhageDPO Results
| ID | model DPO Prediction (%) |
| --- | --- |
| fig|2731619.452.peg.58 | 2 |
| fig|2731619.452.peg.63 | 2 |
| fig|2731619.452.peg.83 | 2 |
| fig|2731619.452.peg.5 | 1 |
| fig|2731619.452.peg.18 | 1 |
| fig|2731619.452.peg.19 | 1 |
| fig|2731619.452.peg.40 | 1 |
| fig|2731619.452.peg.41 | 1 |
| fig|2731619.452.peg.44 | 1 |
| fig|2731619.452.peg.45 | 1 |
| fig|2731619.452.peg.50 | 1 |
| fig|2731619.452.peg.51 | 1 |
| fig|2731619.452.peg.52 | 1 |
| fig|2731619.452.peg.62 | 1 |
| fig|2731619.452.peg.68 | 1 |
| fig|2731619.452.peg.2 | 0 |
| fig|2731619.452.peg.4 | 0 |
| fig|2731619.452.peg.6 | 0 |
| ID | model DPO Prediction (%) |
| --- | --- |
| fig|2731619.452.peg.9 | 0 |
| fig|2731619.452.peg.12 | 0 |
| fig|2731619.452.peg.13 | 0 |
| fig|2731619.452.peg.15 | 0 |
| fig|2731619.452.peg.16 | 0 |
| fig|2731619.452.peg.17 | 0 |
| fig|2731619.452.peg.22 | 0 |
| fig|2731619.452.peg.23 | 0 |
| fig|2731619.452.peg.24 | 0 |
| fig|2731619.452.peg.26 | 0 |
| fig|2731619.452.peg.29 | 0 |
| fig|2731619.452.peg.30 | 0 |
| fig|2731619.452.peg.31 | 0 |
| fig|2731619.452.peg.35 | 0 |
| fig|2731619.452.peg.36 | 0 |
| fig|2731619.452.peg.38 | 0 |
| fig|2731619.452.peg.43 | 0 |
| fig|2731619.452.rna.1 | 0 |

## Slide 14
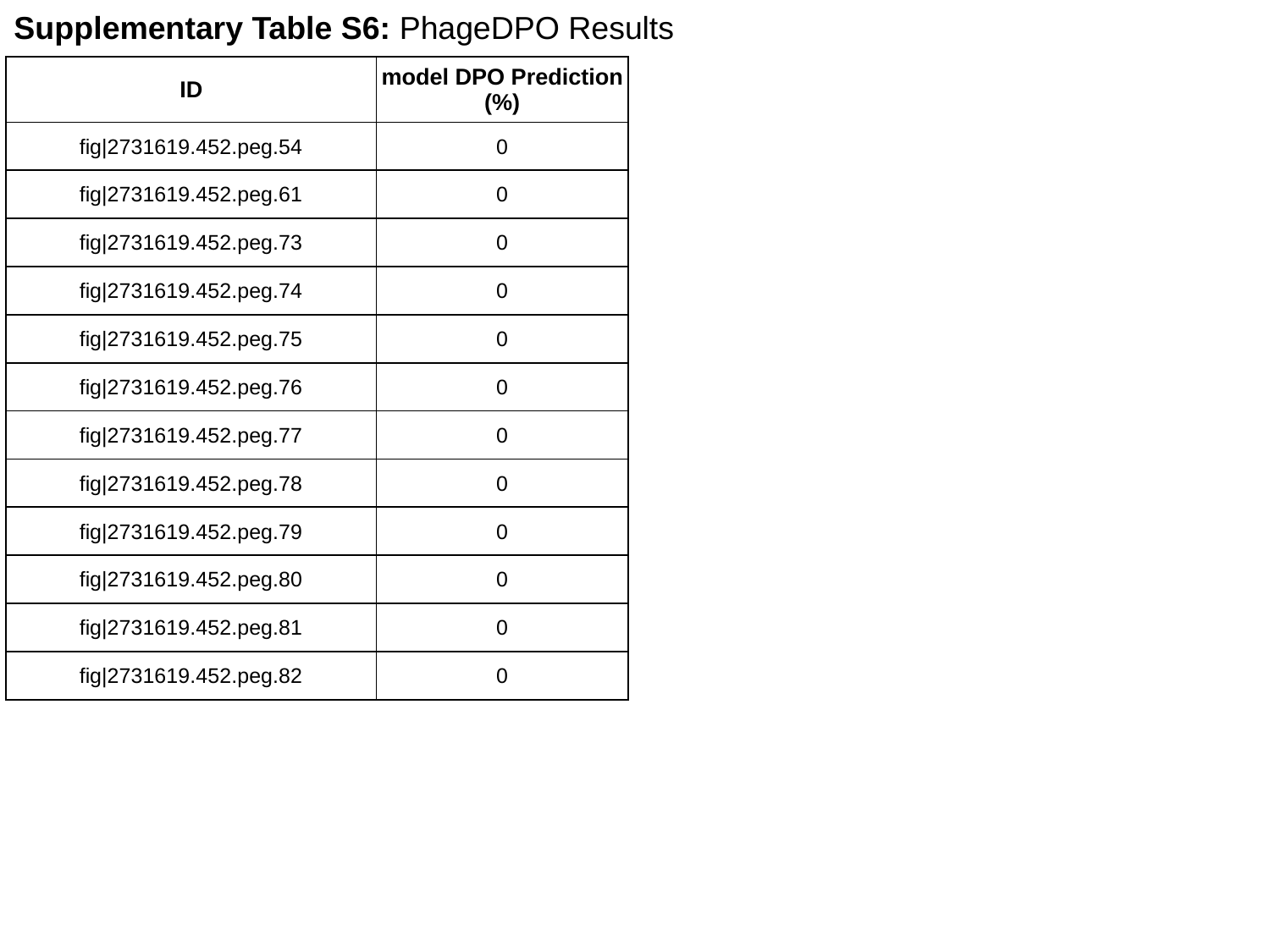

Supplementary Table S6: PhageDPO Results
| ID | model DPO Prediction (%) |
| --- | --- |
| fig|2731619.452.peg.54 | 0 |
| fig|2731619.452.peg.61 | 0 |
| fig|2731619.452.peg.73 | 0 |
| fig|2731619.452.peg.74 | 0 |
| fig|2731619.452.peg.75 | 0 |
| fig|2731619.452.peg.76 | 0 |
| fig|2731619.452.peg.77 | 0 |
| fig|2731619.452.peg.78 | 0 |
| fig|2731619.452.peg.79 | 0 |
| fig|2731619.452.peg.80 | 0 |
| fig|2731619.452.peg.81 | 0 |
| fig|2731619.452.peg.82 | 0 |

## Slide 15
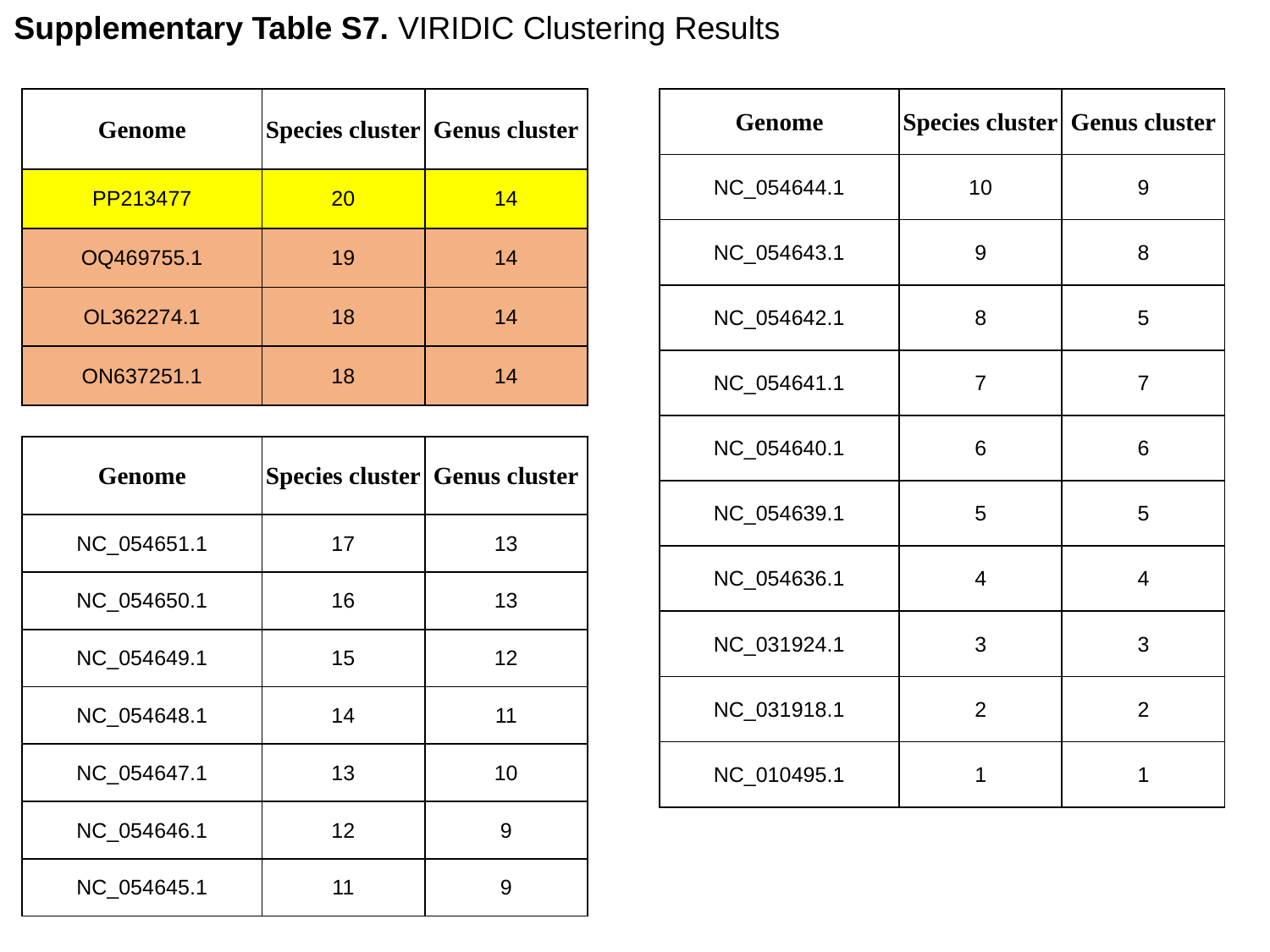

Supplementary Table S7. VIRIDIC Clustering Results
| Genome | Species cluster | Genus cluster |
| --- | --- | --- |
| PP213477 | 20 | 14 |
| OQ469755.1 | 19 | 14 |
| OL362274.1 | 18 | 14 |
| ON637251.1 | 18 | 14 |
| Genome | Species cluster | Genus cluster |
| --- | --- | --- |
| NC\_054644.1 | 10 | 9 |
| NC\_054643.1 | 9 | 8 |
| NC\_054642.1 | 8 | 5 |
| NC\_054641.1 | 7 | 7 |
| NC\_054640.1 | 6 | 6 |
| NC\_054639.1 | 5 | 5 |
| NC\_054636.1 | 4 | 4 |
| NC\_031924.1 | 3 | 3 |
| NC\_031918.1 | 2 | 2 |
| NC\_010495.1 | 1 | 1 |
| Genome | Species cluster | Genus cluster |
| --- | --- | --- |
| NC\_054651.1 | 17 | 13 |
| NC\_054650.1 | 16 | 13 |
| NC\_054649.1 | 15 | 12 |
| NC\_054648.1 | 14 | 11 |
| NC\_054647.1 | 13 | 10 |
| NC\_054646.1 | 12 | 9 |
| NC\_054645.1 | 11 | 9 |

## Slide 16
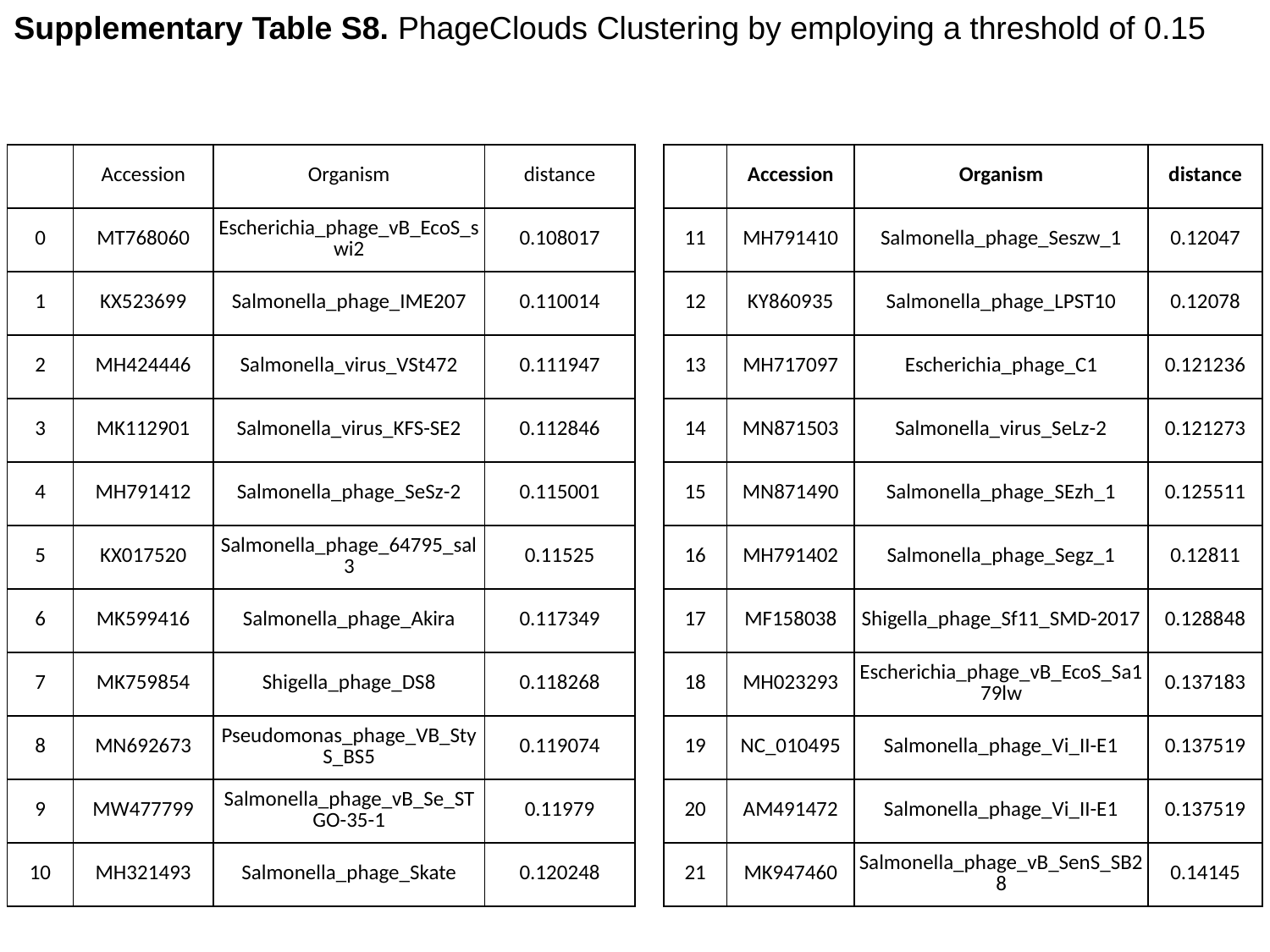

Supplementary Table S8. PhageClouds Clustering by employing a threshold of 0.15
| | Accession | Organism | distance |
| --- | --- | --- | --- |
| 0 | MT768060 | Escherichia\_phage\_vB\_EcoS\_swi2 | 0.108017 |
| 1 | KX523699 | Salmonella\_phage\_IME207 | 0.110014 |
| 2 | MH424446 | Salmonella\_virus\_VSt472 | 0.111947 |
| 3 | MK112901 | Salmonella\_virus\_KFS-SE2 | 0.112846 |
| 4 | MH791412 | Salmonella\_phage\_SeSz-2 | 0.115001 |
| 5 | KX017520 | Salmonella\_phage\_64795\_sal3 | 0.11525 |
| 6 | MK599416 | Salmonella\_phage\_Akira | 0.117349 |
| 7 | MK759854 | Shigella\_phage\_DS8 | 0.118268 |
| 8 | MN692673 | Pseudomonas\_phage\_VB\_StyS\_BS5 | 0.119074 |
| 9 | MW477799 | Salmonella\_phage\_vB\_Se\_STGO-35-1 | 0.11979 |
| 10 | MH321493 | Salmonella\_phage\_Skate | 0.120248 |
| | Accession | Organism | distance |
| --- | --- | --- | --- |
| 11 | MH791410 | Salmonella\_phage\_Seszw\_1 | 0.12047 |
| 12 | KY860935 | Salmonella\_phage\_LPST10 | 0.12078 |
| 13 | MH717097 | Escherichia\_phage\_C1 | 0.121236 |
| 14 | MN871503 | Salmonella\_virus\_SeLz-2 | 0.121273 |
| 15 | MN871490 | Salmonella\_phage\_SEzh\_1 | 0.125511 |
| 16 | MH791402 | Salmonella\_phage\_Segz\_1 | 0.12811 |
| 17 | MF158038 | Shigella\_phage\_Sf11\_SMD-2017 | 0.128848 |
| 18 | MH023293 | Escherichia\_phage\_vB\_EcoS\_Sa179lw | 0.137183 |
| 19 | NC\_010495 | Salmonella\_phage\_Vi\_II-E1 | 0.137519 |
| 20 | AM491472 | Salmonella\_phage\_Vi\_II-E1 | 0.137519 |
| 21 | MK947460 | Salmonella\_phage\_vB\_SenS\_SB28 | 0.14145 |

## Slide 17
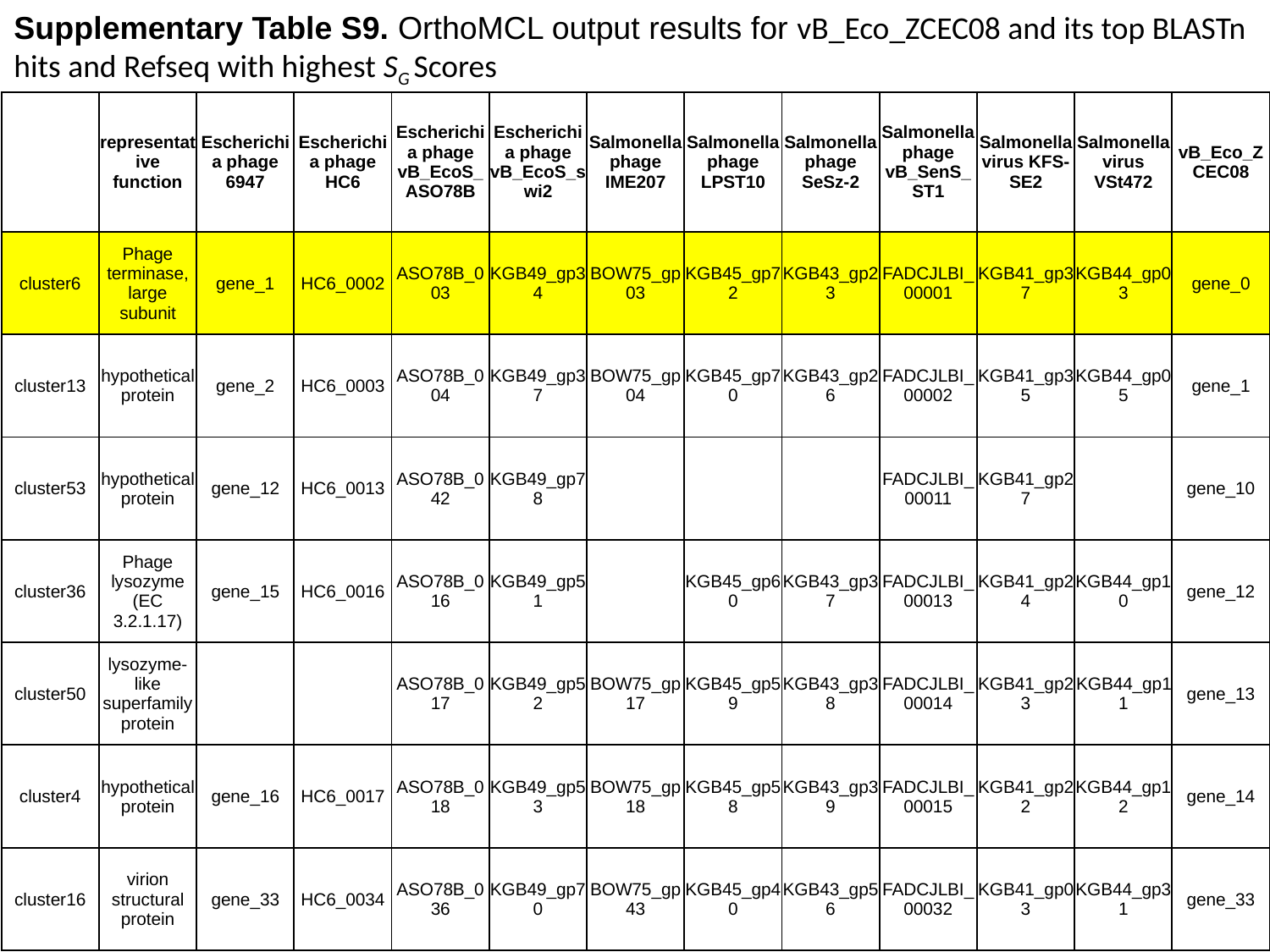

Supplementary Table S9. OrthoMCL output results for vB_Eco_ZCEC08 and its top BLASTn hits and Refseq with highest SG Scores
| | representative function | Escherichia phage 6947 | Escherichia phage HC6 | Escherichia phage vB\_EcoS\_ASO78B | Escherichia phage vB\_EcoS\_swi2 | Salmonella phage IME207 | Salmonella phage LPST10 | Salmonella phage SeSz-2 | Salmonella phage vB\_SenS\_ST1 | Salmonella virus KFS-SE2 | Salmonella virus VSt472 | vB\_Eco\_ZCEC08 |
| --- | --- | --- | --- | --- | --- | --- | --- | --- | --- | --- | --- | --- |
| cluster6 | Phage terminase, large subunit | gene\_1 | HC6\_0002 | ASO78B\_003 | KGB49\_gp34 | BOW75\_gp03 | KGB45\_gp72 | KGB43\_gp23 | FADCJLBI\_00001 | KGB41\_gp37 | KGB44\_gp03 | gene\_0 |
| cluster13 | hypothetical protein | gene\_2 | HC6\_0003 | ASO78B\_004 | KGB49\_gp37 | BOW75\_gp04 | KGB45\_gp70 | KGB43\_gp26 | FADCJLBI\_00002 | KGB41\_gp35 | KGB44\_gp05 | gene\_1 |
| cluster53 | hypothetical protein | gene\_12 | HC6\_0013 | ASO78B\_042 | KGB49\_gp78 | | | | FADCJLBI\_00011 | KGB41\_gp27 | | gene\_10 |
| cluster36 | Phage lysozyme (EC 3.2.1.17) | gene\_15 | HC6\_0016 | ASO78B\_016 | KGB49\_gp51 | | KGB45\_gp60 | KGB43\_gp37 | FADCJLBI\_00013 | KGB41\_gp24 | KGB44\_gp10 | gene\_12 |
| cluster50 | lysozyme-like superfamily protein | | | ASO78B\_017 | KGB49\_gp52 | BOW75\_gp17 | KGB45\_gp59 | KGB43\_gp38 | FADCJLBI\_00014 | KGB41\_gp23 | KGB44\_gp11 | gene\_13 |
| cluster4 | hypothetical protein | gene\_16 | HC6\_0017 | ASO78B\_018 | KGB49\_gp53 | BOW75\_gp18 | KGB45\_gp58 | KGB43\_gp39 | FADCJLBI\_00015 | KGB41\_gp22 | KGB44\_gp12 | gene\_14 |
| cluster16 | virion structural protein | gene\_33 | HC6\_0034 | ASO78B\_036 | KGB49\_gp70 | BOW75\_gp43 | KGB45\_gp40 | KGB43\_gp56 | FADCJLBI\_00032 | KGB41\_gp03 | KGB44\_gp31 | gene\_33 |

## Slide 18
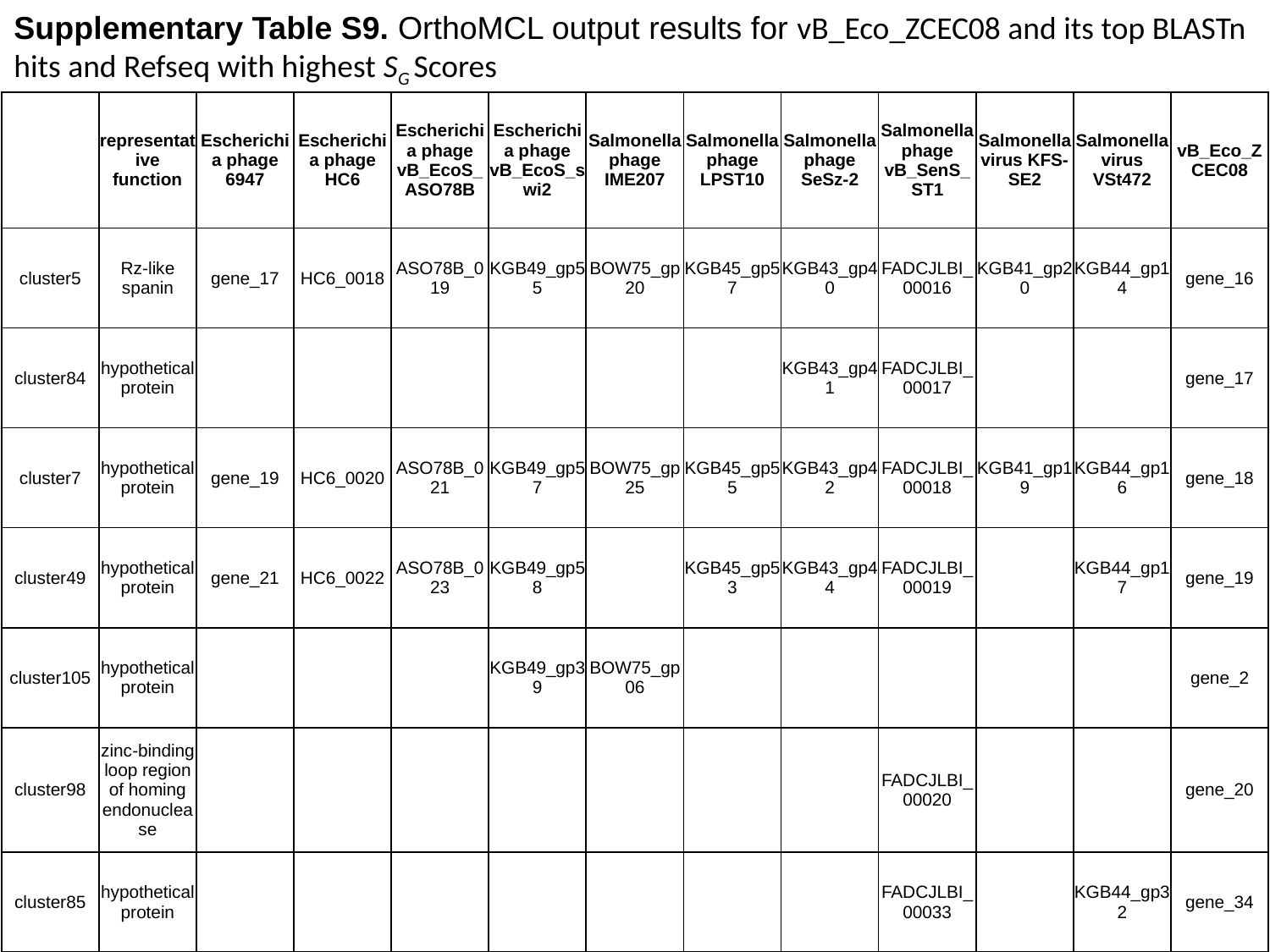

Supplementary Table S9. OrthoMCL output results for vB_Eco_ZCEC08 and its top BLASTn hits and Refseq with highest SG Scores
| | representative function | Escherichia phage 6947 | Escherichia phage HC6 | Escherichia phage vB\_EcoS\_ASO78B | Escherichia phage vB\_EcoS\_swi2 | Salmonella phage IME207 | Salmonella phage LPST10 | Salmonella phage SeSz-2 | Salmonella phage vB\_SenS\_ST1 | Salmonella virus KFS-SE2 | Salmonella virus VSt472 | vB\_Eco\_ZCEC08 |
| --- | --- | --- | --- | --- | --- | --- | --- | --- | --- | --- | --- | --- |
| cluster5 | Rz-like spanin | gene\_17 | HC6\_0018 | ASO78B\_019 | KGB49\_gp55 | BOW75\_gp20 | KGB45\_gp57 | KGB43\_gp40 | FADCJLBI\_00016 | KGB41\_gp20 | KGB44\_gp14 | gene\_16 |
| cluster84 | hypothetical protein | | | | | | | KGB43\_gp41 | FADCJLBI\_00017 | | | gene\_17 |
| cluster7 | hypothetical protein | gene\_19 | HC6\_0020 | ASO78B\_021 | KGB49\_gp57 | BOW75\_gp25 | KGB45\_gp55 | KGB43\_gp42 | FADCJLBI\_00018 | KGB41\_gp19 | KGB44\_gp16 | gene\_18 |
| cluster49 | hypothetical protein | gene\_21 | HC6\_0022 | ASO78B\_023 | KGB49\_gp58 | | KGB45\_gp53 | KGB43\_gp44 | FADCJLBI\_00019 | | KGB44\_gp17 | gene\_19 |
| cluster105 | hypothetical protein | | | | KGB49\_gp39 | BOW75\_gp06 | | | | | | gene\_2 |
| cluster98 | zinc-binding loop region of homing endonuclease | | | | | | | | FADCJLBI\_00020 | | | gene\_20 |
| cluster85 | hypothetical protein | | | | | | | | FADCJLBI\_00033 | | KGB44\_gp32 | gene\_34 |

## Slide 19
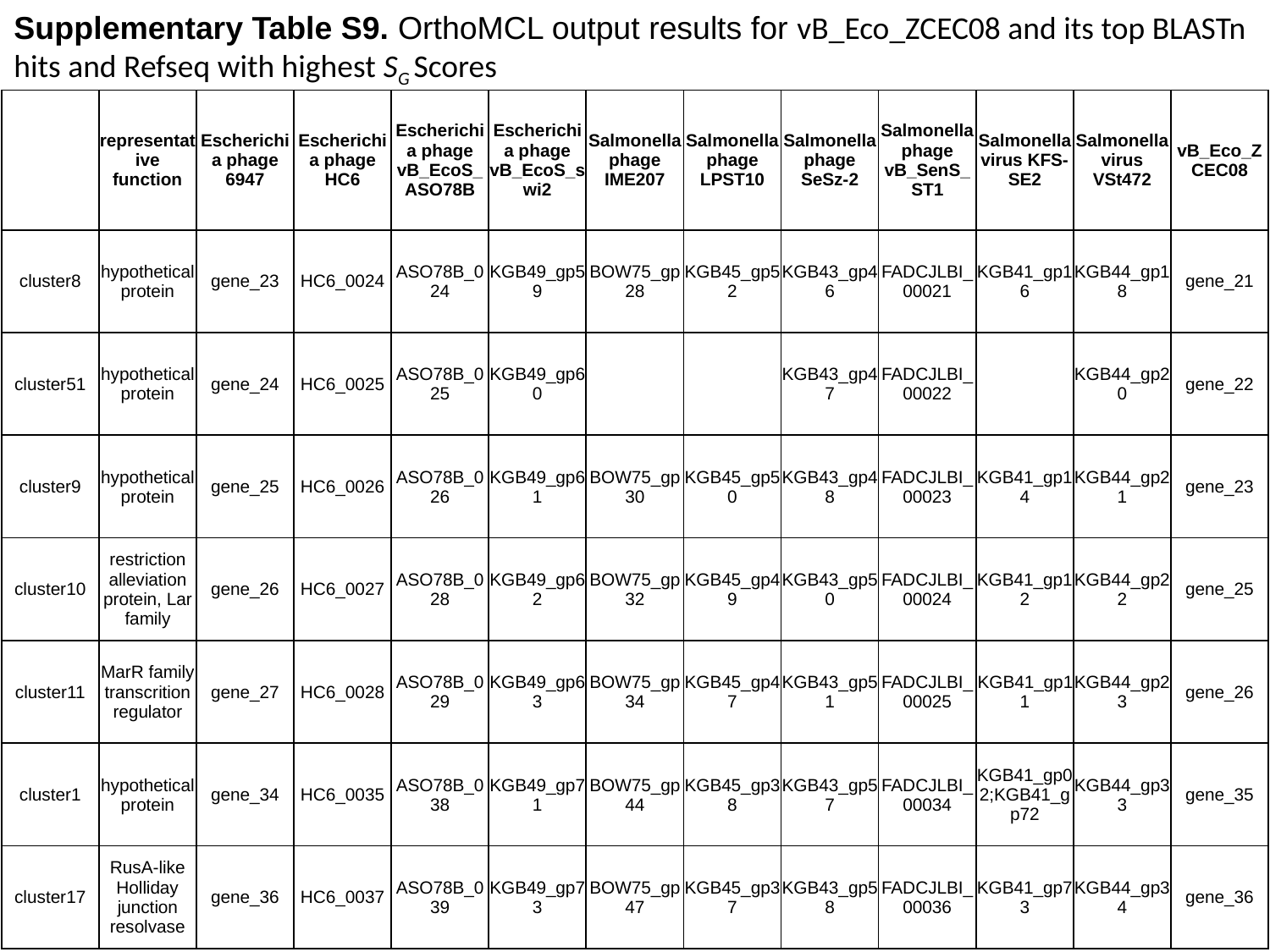

Supplementary Table S9. OrthoMCL output results for vB_Eco_ZCEC08 and its top BLASTn hits and Refseq with highest SG Scores
| | representative function | Escherichia phage 6947 | Escherichia phage HC6 | Escherichia phage vB\_EcoS\_ASO78B | Escherichia phage vB\_EcoS\_swi2 | Salmonella phage IME207 | Salmonella phage LPST10 | Salmonella phage SeSz-2 | Salmonella phage vB\_SenS\_ST1 | Salmonella virus KFS-SE2 | Salmonella virus VSt472 | vB\_Eco\_ZCEC08 |
| --- | --- | --- | --- | --- | --- | --- | --- | --- | --- | --- | --- | --- |
| cluster8 | hypothetical protein | gene\_23 | HC6\_0024 | ASO78B\_024 | KGB49\_gp59 | BOW75\_gp28 | KGB45\_gp52 | KGB43\_gp46 | FADCJLBI\_00021 | KGB41\_gp16 | KGB44\_gp18 | gene\_21 |
| cluster51 | hypothetical protein | gene\_24 | HC6\_0025 | ASO78B\_025 | KGB49\_gp60 | | | KGB43\_gp47 | FADCJLBI\_00022 | | KGB44\_gp20 | gene\_22 |
| cluster9 | hypothetical protein | gene\_25 | HC6\_0026 | ASO78B\_026 | KGB49\_gp61 | BOW75\_gp30 | KGB45\_gp50 | KGB43\_gp48 | FADCJLBI\_00023 | KGB41\_gp14 | KGB44\_gp21 | gene\_23 |
| cluster10 | restriction alleviation protein, Lar family | gene\_26 | HC6\_0027 | ASO78B\_028 | KGB49\_gp62 | BOW75\_gp32 | KGB45\_gp49 | KGB43\_gp50 | FADCJLBI\_00024 | KGB41\_gp12 | KGB44\_gp22 | gene\_25 |
| cluster11 | MarR family transcrition regulator | gene\_27 | HC6\_0028 | ASO78B\_029 | KGB49\_gp63 | BOW75\_gp34 | KGB45\_gp47 | KGB43\_gp51 | FADCJLBI\_00025 | KGB41\_gp11 | KGB44\_gp23 | gene\_26 |
| cluster1 | hypothetical protein | gene\_34 | HC6\_0035 | ASO78B\_038 | KGB49\_gp71 | BOW75\_gp44 | KGB45\_gp38 | KGB43\_gp57 | FADCJLBI\_00034 | KGB41\_gp02;KGB41\_gp72 | KGB44\_gp33 | gene\_35 |
| cluster17 | RusA-like Holliday junction resolvase | gene\_36 | HC6\_0037 | ASO78B\_039 | KGB49\_gp73 | BOW75\_gp47 | KGB45\_gp37 | KGB43\_gp58 | FADCJLBI\_00036 | KGB41\_gp73 | KGB44\_gp34 | gene\_36 |

## Slide 20
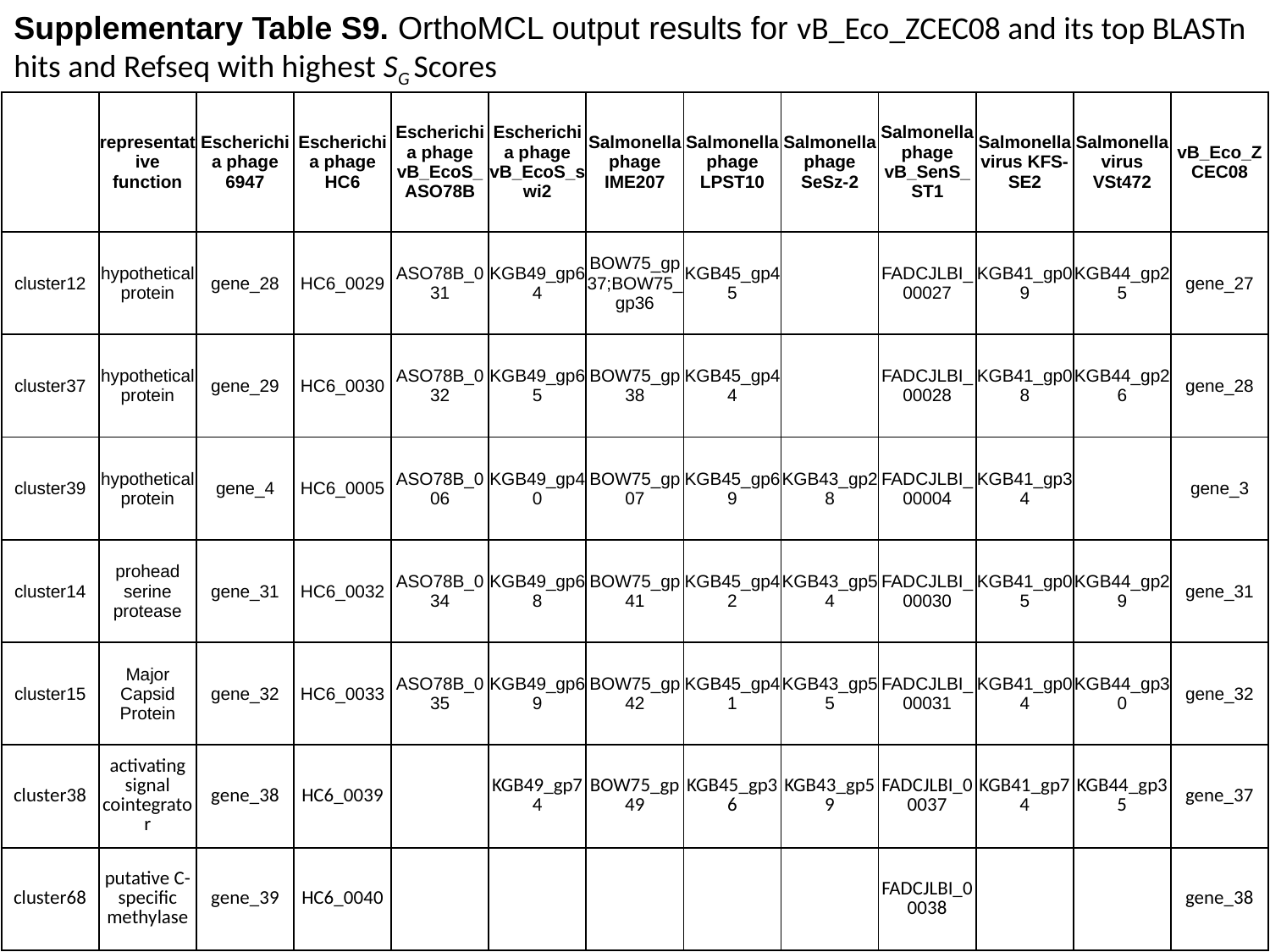

Supplementary Table S9. OrthoMCL output results for vB_Eco_ZCEC08 and its top BLASTn hits and Refseq with highest SG Scores
| | representative function | Escherichia phage 6947 | Escherichia phage HC6 | Escherichia phage vB\_EcoS\_ASO78B | Escherichia phage vB\_EcoS\_swi2 | Salmonella phage IME207 | Salmonella phage LPST10 | Salmonella phage SeSz-2 | Salmonella phage vB\_SenS\_ST1 | Salmonella virus KFS-SE2 | Salmonella virus VSt472 | vB\_Eco\_ZCEC08 |
| --- | --- | --- | --- | --- | --- | --- | --- | --- | --- | --- | --- | --- |
| cluster12 | hypothetical protein | gene\_28 | HC6\_0029 | ASO78B\_031 | KGB49\_gp64 | BOW75\_gp37;BOW75\_gp36 | KGB45\_gp45 | | FADCJLBI\_00027 | KGB41\_gp09 | KGB44\_gp25 | gene\_27 |
| cluster37 | hypothetical protein | gene\_29 | HC6\_0030 | ASO78B\_032 | KGB49\_gp65 | BOW75\_gp38 | KGB45\_gp44 | | FADCJLBI\_00028 | KGB41\_gp08 | KGB44\_gp26 | gene\_28 |
| cluster39 | hypothetical protein | gene\_4 | HC6\_0005 | ASO78B\_006 | KGB49\_gp40 | BOW75\_gp07 | KGB45\_gp69 | KGB43\_gp28 | FADCJLBI\_00004 | KGB41\_gp34 | | gene\_3 |
| cluster14 | prohead serine protease | gene\_31 | HC6\_0032 | ASO78B\_034 | KGB49\_gp68 | BOW75\_gp41 | KGB45\_gp42 | KGB43\_gp54 | FADCJLBI\_00030 | KGB41\_gp05 | KGB44\_gp29 | gene\_31 |
| cluster15 | Major Capsid Protein | gene\_32 | HC6\_0033 | ASO78B\_035 | KGB49\_gp69 | BOW75\_gp42 | KGB45\_gp41 | KGB43\_gp55 | FADCJLBI\_00031 | KGB41\_gp04 | KGB44\_gp30 | gene\_32 |
| cluster38 | activating signal cointegrator | gene\_38 | HC6\_0039 | | KGB49\_gp74 | BOW75\_gp49 | KGB45\_gp36 | KGB43\_gp59 | FADCJLBI\_00037 | KGB41\_gp74 | KGB44\_gp35 | gene\_37 |
| cluster68 | putative C-specific methylase | gene\_39 | HC6\_0040 | | | | | | FADCJLBI\_00038 | | | gene\_38 |

## Slide 21
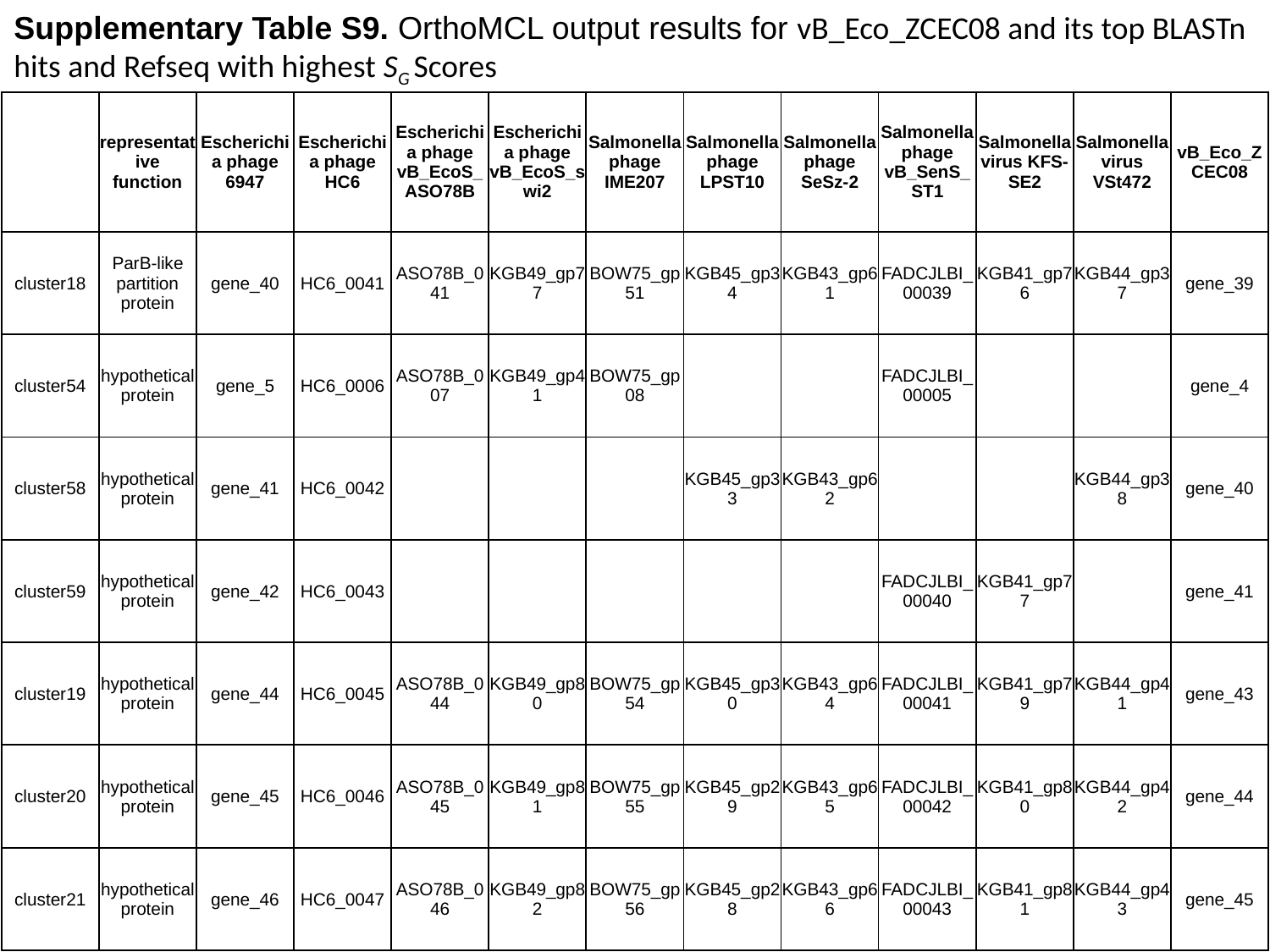

Supplementary Table S9. OrthoMCL output results for vB_Eco_ZCEC08 and its top BLASTn hits and Refseq with highest SG Scores
| | representative function | Escherichia phage 6947 | Escherichia phage HC6 | Escherichia phage vB\_EcoS\_ASO78B | Escherichia phage vB\_EcoS\_swi2 | Salmonella phage IME207 | Salmonella phage LPST10 | Salmonella phage SeSz-2 | Salmonella phage vB\_SenS\_ST1 | Salmonella virus KFS-SE2 | Salmonella virus VSt472 | vB\_Eco\_ZCEC08 |
| --- | --- | --- | --- | --- | --- | --- | --- | --- | --- | --- | --- | --- |
| cluster18 | ParB-like partition protein | gene\_40 | HC6\_0041 | ASO78B\_041 | KGB49\_gp77 | BOW75\_gp51 | KGB45\_gp34 | KGB43\_gp61 | FADCJLBI\_00039 | KGB41\_gp76 | KGB44\_gp37 | gene\_39 |
| cluster54 | hypothetical protein | gene\_5 | HC6\_0006 | ASO78B\_007 | KGB49\_gp41 | BOW75\_gp08 | | | FADCJLBI\_00005 | | | gene\_4 |
| cluster58 | hypothetical protein | gene\_41 | HC6\_0042 | | | | KGB45\_gp33 | KGB43\_gp62 | | | KGB44\_gp38 | gene\_40 |
| cluster59 | hypothetical protein | gene\_42 | HC6\_0043 | | | | | | FADCJLBI\_00040 | KGB41\_gp77 | | gene\_41 |
| cluster19 | hypothetical protein | gene\_44 | HC6\_0045 | ASO78B\_044 | KGB49\_gp80 | BOW75\_gp54 | KGB45\_gp30 | KGB43\_gp64 | FADCJLBI\_00041 | KGB41\_gp79 | KGB44\_gp41 | gene\_43 |
| cluster20 | hypothetical protein | gene\_45 | HC6\_0046 | ASO78B\_045 | KGB49\_gp81 | BOW75\_gp55 | KGB45\_gp29 | KGB43\_gp65 | FADCJLBI\_00042 | KGB41\_gp80 | KGB44\_gp42 | gene\_44 |
| cluster21 | hypothetical protein | gene\_46 | HC6\_0047 | ASO78B\_046 | KGB49\_gp82 | BOW75\_gp56 | KGB45\_gp28 | KGB43\_gp66 | FADCJLBI\_00043 | KGB41\_gp81 | KGB44\_gp43 | gene\_45 |

## Slide 22
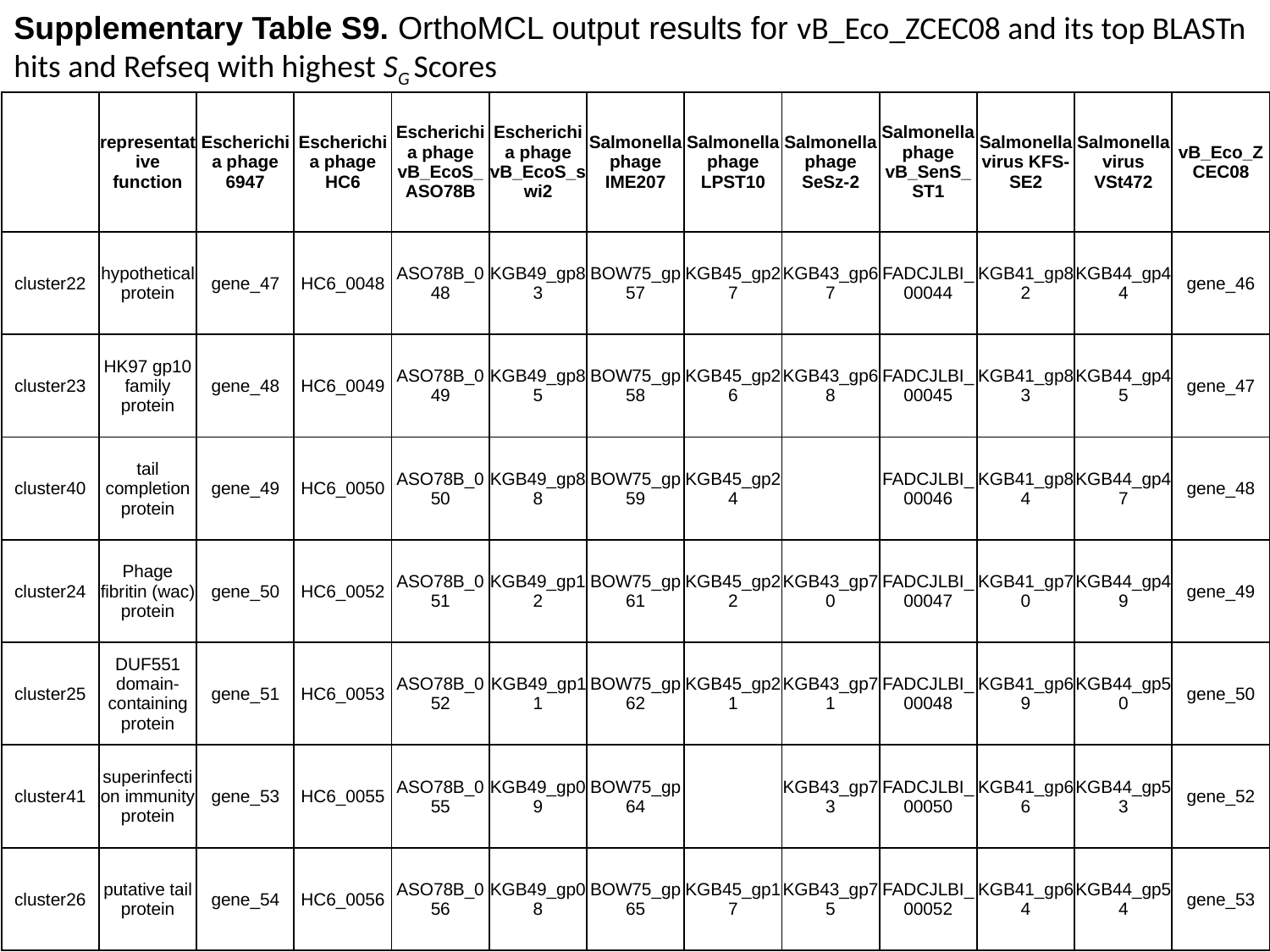

Supplementary Table S9. OrthoMCL output results for vB_Eco_ZCEC08 and its top BLASTn hits and Refseq with highest SG Scores
| | representative function | Escherichia phage 6947 | Escherichia phage HC6 | Escherichia phage vB\_EcoS\_ASO78B | Escherichia phage vB\_EcoS\_swi2 | Salmonella phage IME207 | Salmonella phage LPST10 | Salmonella phage SeSz-2 | Salmonella phage vB\_SenS\_ST1 | Salmonella virus KFS-SE2 | Salmonella virus VSt472 | vB\_Eco\_ZCEC08 |
| --- | --- | --- | --- | --- | --- | --- | --- | --- | --- | --- | --- | --- |
| cluster22 | hypothetical protein | gene\_47 | HC6\_0048 | ASO78B\_048 | KGB49\_gp83 | BOW75\_gp57 | KGB45\_gp27 | KGB43\_gp67 | FADCJLBI\_00044 | KGB41\_gp82 | KGB44\_gp44 | gene\_46 |
| cluster23 | HK97 gp10 family protein | gene\_48 | HC6\_0049 | ASO78B\_049 | KGB49\_gp85 | BOW75\_gp58 | KGB45\_gp26 | KGB43\_gp68 | FADCJLBI\_00045 | KGB41\_gp83 | KGB44\_gp45 | gene\_47 |
| cluster40 | tail completion protein | gene\_49 | HC6\_0050 | ASO78B\_050 | KGB49\_gp88 | BOW75\_gp59 | KGB45\_gp24 | | FADCJLBI\_00046 | KGB41\_gp84 | KGB44\_gp47 | gene\_48 |
| cluster24 | Phage fibritin (wac) protein | gene\_50 | HC6\_0052 | ASO78B\_051 | KGB49\_gp12 | BOW75\_gp61 | KGB45\_gp22 | KGB43\_gp70 | FADCJLBI\_00047 | KGB41\_gp70 | KGB44\_gp49 | gene\_49 |
| cluster25 | DUF551 domain-containing protein | gene\_51 | HC6\_0053 | ASO78B\_052 | KGB49\_gp11 | BOW75\_gp62 | KGB45\_gp21 | KGB43\_gp71 | FADCJLBI\_00048 | KGB41\_gp69 | KGB44\_gp50 | gene\_50 |
| cluster41 | superinfection immunity protein | gene\_53 | HC6\_0055 | ASO78B\_055 | KGB49\_gp09 | BOW75\_gp64 | | KGB43\_gp73 | FADCJLBI\_00050 | KGB41\_gp66 | KGB44\_gp53 | gene\_52 |
| cluster26 | putative tail protein | gene\_54 | HC6\_0056 | ASO78B\_056 | KGB49\_gp08 | BOW75\_gp65 | KGB45\_gp17 | KGB43\_gp75 | FADCJLBI\_00052 | KGB41\_gp64 | KGB44\_gp54 | gene\_53 |

## Slide 23
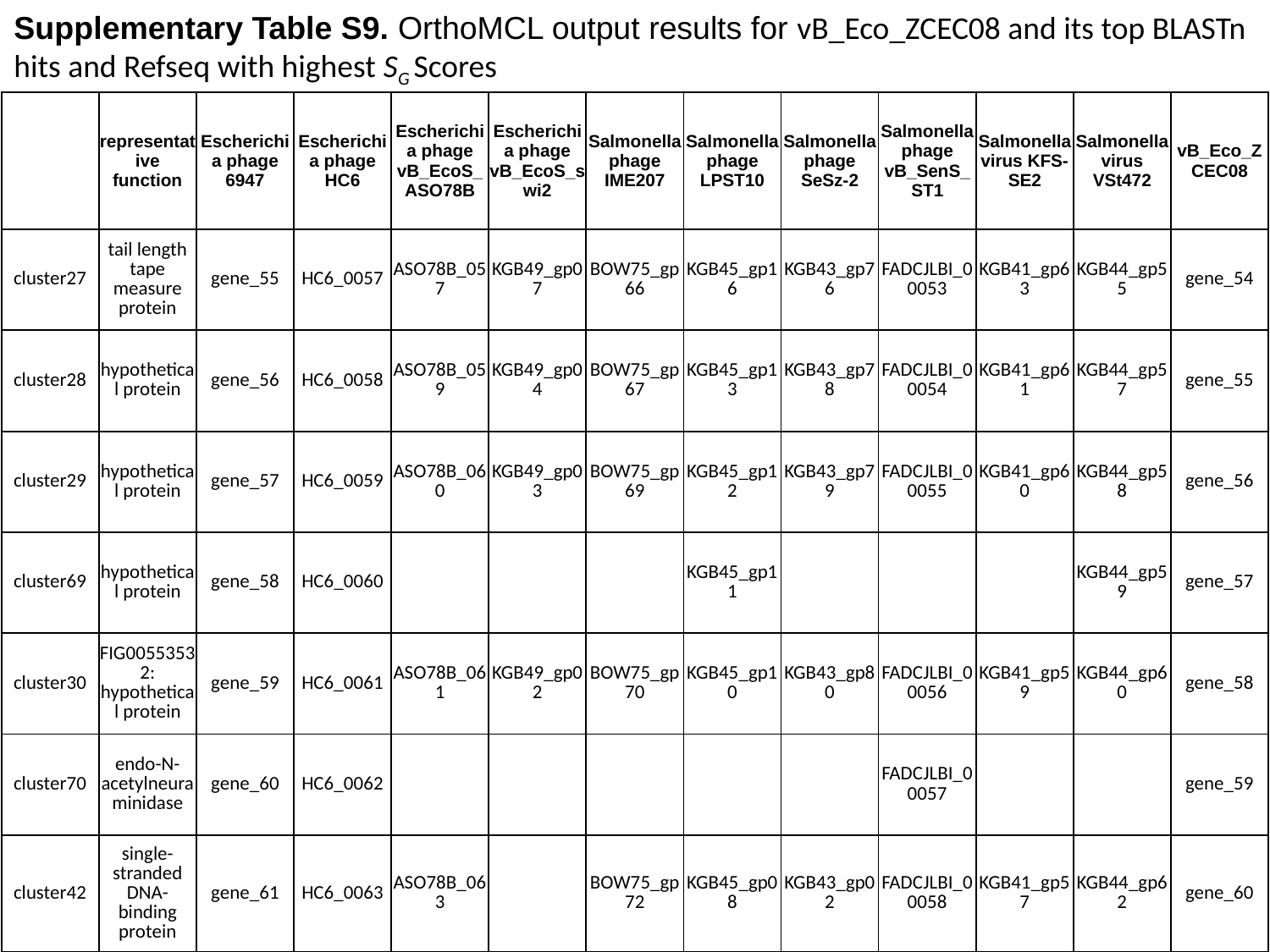

Supplementary Table S9. OrthoMCL output results for vB_Eco_ZCEC08 and its top BLASTn hits and Refseq with highest SG Scores
| | representative function | Escherichia phage 6947 | Escherichia phage HC6 | Escherichia phage vB\_EcoS\_ASO78B | Escherichia phage vB\_EcoS\_swi2 | Salmonella phage IME207 | Salmonella phage LPST10 | Salmonella phage SeSz-2 | Salmonella phage vB\_SenS\_ST1 | Salmonella virus KFS-SE2 | Salmonella virus VSt472 | vB\_Eco\_ZCEC08 |
| --- | --- | --- | --- | --- | --- | --- | --- | --- | --- | --- | --- | --- |
| cluster27 | tail length tape measure protein | gene\_55 | HC6\_0057 | ASO78B\_057 | KGB49\_gp07 | BOW75\_gp66 | KGB45\_gp16 | KGB43\_gp76 | FADCJLBI\_00053 | KGB41\_gp63 | KGB44\_gp55 | gene\_54 |
| cluster28 | hypothetical protein | gene\_56 | HC6\_0058 | ASO78B\_059 | KGB49\_gp04 | BOW75\_gp67 | KGB45\_gp13 | KGB43\_gp78 | FADCJLBI\_00054 | KGB41\_gp61 | KGB44\_gp57 | gene\_55 |
| cluster29 | hypothetical protein | gene\_57 | HC6\_0059 | ASO78B\_060 | KGB49\_gp03 | BOW75\_gp69 | KGB45\_gp12 | KGB43\_gp79 | FADCJLBI\_00055 | KGB41\_gp60 | KGB44\_gp58 | gene\_56 |
| cluster69 | hypothetical protein | gene\_58 | HC6\_0060 | | | | KGB45\_gp11 | | | | KGB44\_gp59 | gene\_57 |
| cluster30 | FIG00553532: hypothetical protein | gene\_59 | HC6\_0061 | ASO78B\_061 | KGB49\_gp02 | BOW75\_gp70 | KGB45\_gp10 | KGB43\_gp80 | FADCJLBI\_00056 | KGB41\_gp59 | KGB44\_gp60 | gene\_58 |
| cluster70 | endo-N-acetylneuraminidase | gene\_60 | HC6\_0062 | | | | | | FADCJLBI\_00057 | | | gene\_59 |
| cluster42 | single-stranded DNA-binding protein | gene\_61 | HC6\_0063 | ASO78B\_063 | | BOW75\_gp72 | KGB45\_gp08 | KGB43\_gp02 | FADCJLBI\_00058 | KGB41\_gp57 | KGB44\_gp62 | gene\_60 |

## Slide 24
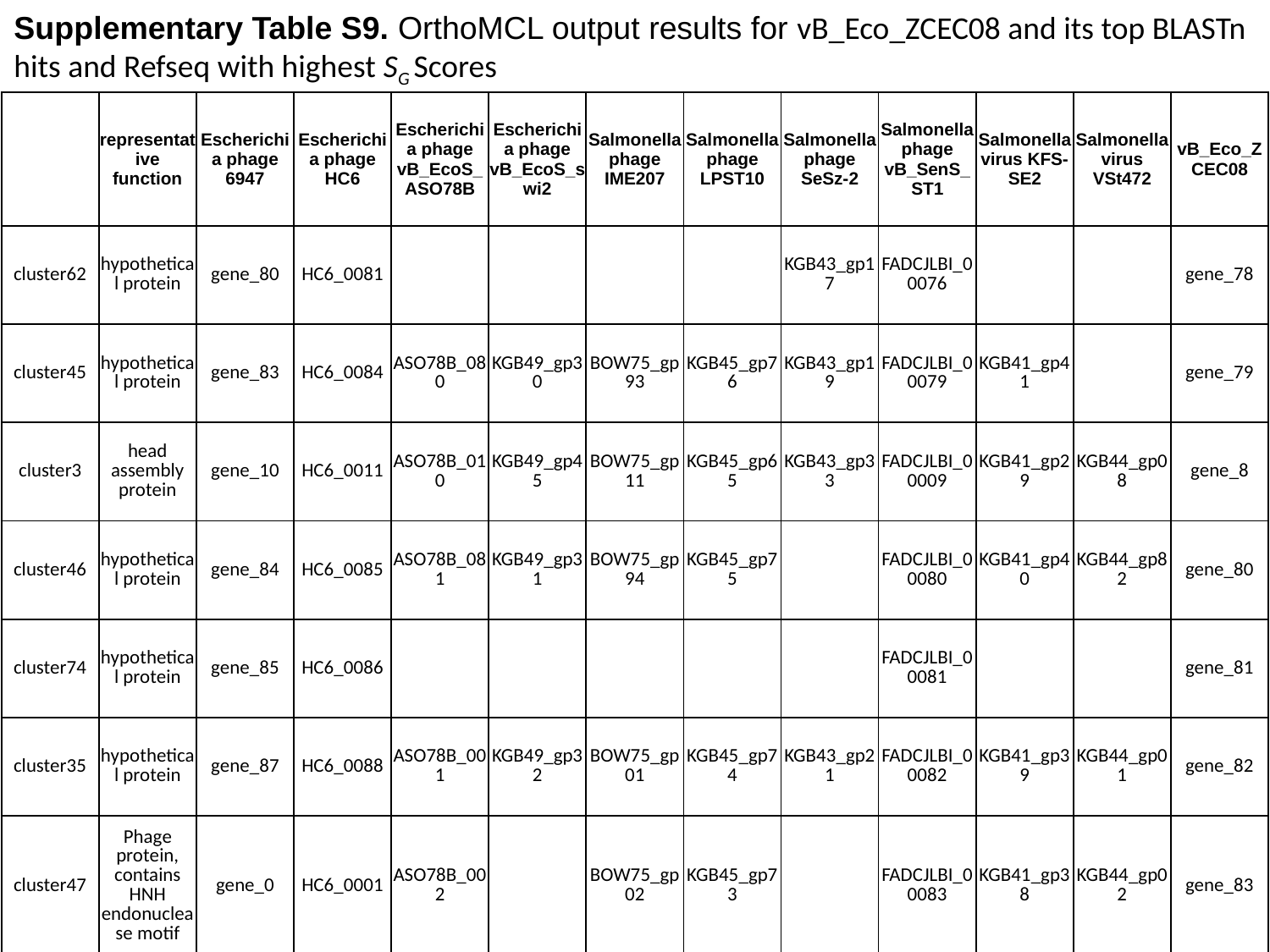

Supplementary Table S9. OrthoMCL output results for vB_Eco_ZCEC08 and its top BLASTn hits and Refseq with highest SG Scores
| | representative function | Escherichia phage 6947 | Escherichia phage HC6 | Escherichia phage vB\_EcoS\_ASO78B | Escherichia phage vB\_EcoS\_swi2 | Salmonella phage IME207 | Salmonella phage LPST10 | Salmonella phage SeSz-2 | Salmonella phage vB\_SenS\_ST1 | Salmonella virus KFS-SE2 | Salmonella virus VSt472 | vB\_Eco\_ZCEC08 |
| --- | --- | --- | --- | --- | --- | --- | --- | --- | --- | --- | --- | --- |
| cluster62 | hypothetical protein | gene\_80 | HC6\_0081 | | | | | KGB43\_gp17 | FADCJLBI\_00076 | | | gene\_78 |
| cluster45 | hypothetical protein | gene\_83 | HC6\_0084 | ASO78B\_080 | KGB49\_gp30 | BOW75\_gp93 | KGB45\_gp76 | KGB43\_gp19 | FADCJLBI\_00079 | KGB41\_gp41 | | gene\_79 |
| cluster3 | head assembly protein | gene\_10 | HC6\_0011 | ASO78B\_010 | KGB49\_gp45 | BOW75\_gp11 | KGB45\_gp65 | KGB43\_gp33 | FADCJLBI\_00009 | KGB41\_gp29 | KGB44\_gp08 | gene\_8 |
| cluster46 | hypothetical protein | gene\_84 | HC6\_0085 | ASO78B\_081 | KGB49\_gp31 | BOW75\_gp94 | KGB45\_gp75 | | FADCJLBI\_00080 | KGB41\_gp40 | KGB44\_gp82 | gene\_80 |
| cluster74 | hypothetical protein | gene\_85 | HC6\_0086 | | | | | | FADCJLBI\_00081 | | | gene\_81 |
| cluster35 | hypothetical protein | gene\_87 | HC6\_0088 | ASO78B\_001 | KGB49\_gp32 | BOW75\_gp01 | KGB45\_gp74 | KGB43\_gp21 | FADCJLBI\_00082 | KGB41\_gp39 | KGB44\_gp01 | gene\_82 |
| cluster47 | Phage protein, contains HNH endonuclease motif | gene\_0 | HC6\_0001 | ASO78B\_002 | | BOW75\_gp02 | KGB45\_gp73 | | FADCJLBI\_00083 | KGB41\_gp38 | KGB44\_gp02 | gene\_83 |

## Slide 25
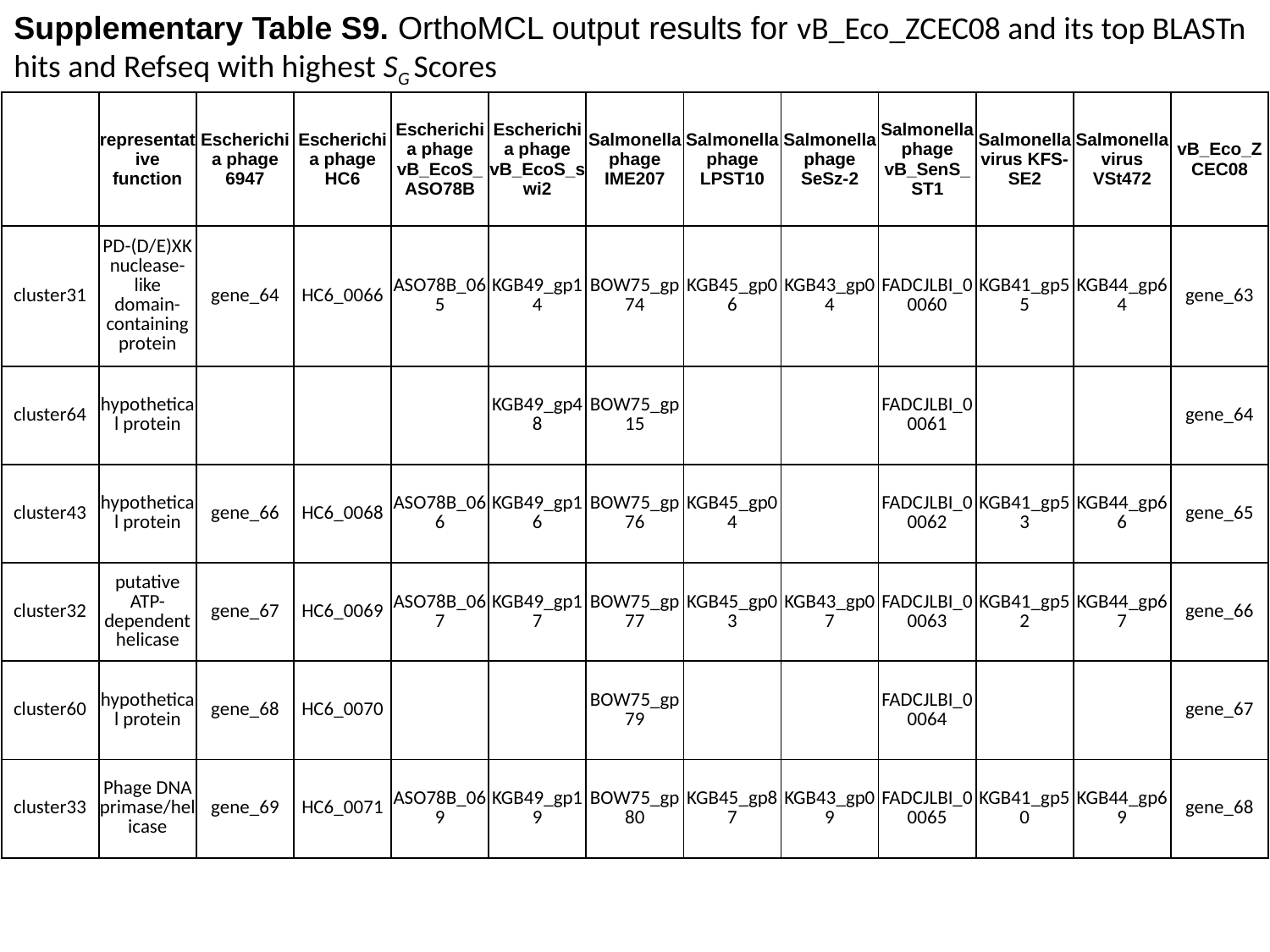

Supplementary Table S9. OrthoMCL output results for vB_Eco_ZCEC08 and its top BLASTn hits and Refseq with highest SG Scores
| | representative function | Escherichia phage 6947 | Escherichia phage HC6 | Escherichia phage vB\_EcoS\_ASO78B | Escherichia phage vB\_EcoS\_swi2 | Salmonella phage IME207 | Salmonella phage LPST10 | Salmonella phage SeSz-2 | Salmonella phage vB\_SenS\_ST1 | Salmonella virus KFS-SE2 | Salmonella virus VSt472 | vB\_Eco\_ZCEC08 |
| --- | --- | --- | --- | --- | --- | --- | --- | --- | --- | --- | --- | --- |
| cluster31 | PD-(D/E)XK nuclease-like domain-containing protein | gene\_64 | HC6\_0066 | ASO78B\_065 | KGB49\_gp14 | BOW75\_gp74 | KGB45\_gp06 | KGB43\_gp04 | FADCJLBI\_00060 | KGB41\_gp55 | KGB44\_gp64 | gene\_63 |
| cluster64 | hypothetical protein | | | | KGB49\_gp48 | BOW75\_gp15 | | | FADCJLBI\_00061 | | | gene\_64 |
| cluster43 | hypothetical protein | gene\_66 | HC6\_0068 | ASO78B\_066 | KGB49\_gp16 | BOW75\_gp76 | KGB45\_gp04 | | FADCJLBI\_00062 | KGB41\_gp53 | KGB44\_gp66 | gene\_65 |
| cluster32 | putative ATP-dependent helicase | gene\_67 | HC6\_0069 | ASO78B\_067 | KGB49\_gp17 | BOW75\_gp77 | KGB45\_gp03 | KGB43\_gp07 | FADCJLBI\_00063 | KGB41\_gp52 | KGB44\_gp67 | gene\_66 |
| cluster60 | hypothetical protein | gene\_68 | HC6\_0070 | | | BOW75\_gp79 | | | FADCJLBI\_00064 | | | gene\_67 |
| cluster33 | Phage DNA primase/helicase | gene\_69 | HC6\_0071 | ASO78B\_069 | KGB49\_gp19 | BOW75\_gp80 | KGB45\_gp87 | KGB43\_gp09 | FADCJLBI\_00065 | KGB41\_gp50 | KGB44\_gp69 | gene\_68 |

## Slide 26
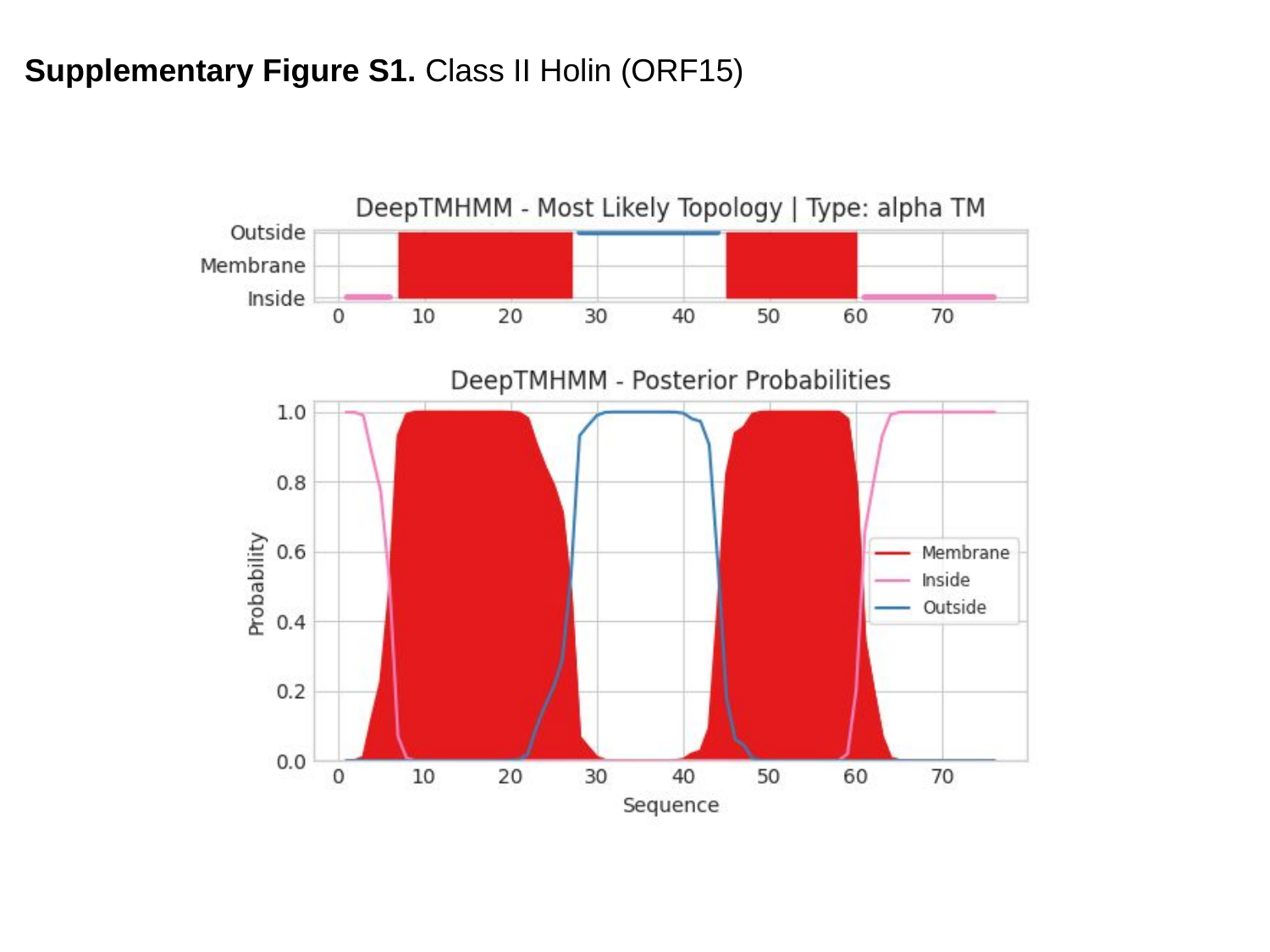

Supplementary Figure S1. Class II Holin (ORF15)
